# Assessment tools for neonatal resuscitation and their validity evidence: a scoping review

**DOI:** 10.1016/j.resplu.2026.101304

**Published:** 2026-03-25

**Authors:** Maud Steins, Lieke Raaijmakers, Marije Hogeveen, Mathijs Binkhorst

**Affiliations:** a Department of Pediatrics, Radboud University Medical Center Amalia Children’s Hospital, Geert Grooteplein Zuid 10, 6525 GA Nijmegen, the Netherlands; b Department of Pediatrics, Division of Neonatology, Radboud University Medical Center Amalia Children’s Hospital, Geert Grooteplein Zuid 10, 6525 GA Nijmegen, the Netherlands

**Keywords:** Neonatal resuscitation, Validation, Assessment, Reliability, Performance

## Abstract

**Objective:**

Competence in neonatal resuscitation is acquired and maintained through accredited life support courses and short in-situ booster training sessions. To ensure effective knowledge and skills integration into practice, valid tools for assessing performance are essential. The aim of this scoping review was to identify all available tools assessing overall performance neonatal resuscitation and evaluate their reported validity evidence.

**Methods:**

In January 2024 and July 2025, MEDLINE, Embase, and the Cochrane Library were searched to identify studies describing assessment tools for neonatal resuscitation and their associated validity evidence. All study types describing neonatal resuscitation assessment tools for simulation and clinical practice were included. Two reviewers independently screened titles and abstract followed by full-text screening. Data were extracted using predefined forms.

**Results:**

A total of 146 sources were included, identifying 82 unique assessment tools for neonatal resuscitation. The number of included items in these tools varied widely, ranging from 5 to 133 (median = 22, IQR = 15–36). Some form of validity evidence was reported for 73.2% of these tools. Most studies reported items from classical frameworks for validity evidence; however, only 30.0% reported more than two items of these frameworks. Contemporary frameworks for validity evidence, considered the preferred standard, were only (partly) applied in three studies.

**Conclusions:**

Multiple assessment tools for neonatal resuscitation have been reported, most lack proper validity evidence. There is a clear need for further research and development of a comprehensive, up-to-date, properly validated, and widely applicable assessment tool to support high-quality training, reliable assessment, and ultimately improve patient outcomes.

## Highlights

• Tools vary in content, structure, and intended use.
• All tools assess multiple competencies, but none covers all resuscitation skills.
• Most tools lack proper validity evidence.
• Contemporary validity frameworks are preferred, but rarely used.
• An up-to-date, validated tool is needed, applicable across multiple settings.

## Introduction

Neonatal resuscitation is an essential competence for all healthcare professionals (HCP) involved in delivery area management. It comprises both the support of physiological transition and emergency care for infants who are compromised immediately after birth. This includes interventions for umbilical cord management, thermal control, airway management, positive pressure ventilation (PPV), chest compressions (CC) and medication. Interventions are necessary in approximately 11% of newborns. Most infants respond to initial steps such as drying, stimulation, and airway maneuvers (e.g., oxygen supplementation 8%, continuous positive airway pressure 7%, suctioning 6%, and non-invasive ventilation 4%), while further steps, such as intubation (1%), CC (0.1%), and adrenaline (0.1%) are rare.^1^

The two most commonly used algorithms for neonatal resuscitation in high-resource settings are developed by the European Resuscitation Council (ERC) and the American Heart Association (AHA) together with the American Academy of Pediatrics (AAP).^1^, ^2^ Competency in the accompanying knowledge, technical, and non-technical skills can be acquired and maintained through participation in accredited life support courses, such as the Newborn Life Support (NLS) course (ERC) and the Neonatal Resuscitation Program (NRP) (AHA / AAP). These courses typically end with a simulation-based practical exam or Objective Structured Clinical Examination (OSCE) to assess algorithm adherence and appropriate skill performance. Short in-situ booster training aids in the retention of knowledge and skills and similarly requires some form of assessment.

In low-resource settings, the Helping Babies Breathe (HBB) program provides the most widely used structured approach to neonatal resuscitation. Designed specifically for environments with limited resources, it focuses on thermoregulation, stimulation, bag-and-mask ventilation (BMV), and ongoing evaluation.^3^ A multiple-choice questionnaire, BMV checklist and two OSCEs are used to assess HBB competence.^4^

Neonatal resuscitation training is associated with improved patient outcomes and reductions in neonatal morbidity and mortality.^5^, ^6^, ^7^, ^8^ As mentioned, assessment tools are essential for evaluating performance, with applications across qualitative, quantitative, formative, and summative assessments.^9^ These tools support feedback for learning, help identify learning needs, and enable objective comparison of skills across individuals, instructional methods, and time points, in clinical, simulation and research settings. In addition, they also serve as benchmarks for skills levels, supporting quality assurance in resuscitation performance. Formal assessment is often required for successful course completion and professional registration. Finally, assessment can act as learning motivator and stimulate retention through test-enhanced learning.^10^

The validity of assessment (tools) is paramount, as validity evidence is key to evaluate “the appropriateness of the interpretations, uses, and decisions based on assessment results”.^11^ In healthcare education, valid assessment tools ensure that knowledge and skills acquired during training can be transferred effectively to clinical practice, thereby contributing to improved patient care.^12^ Several frameworks have been created to classify types of validity evidence. Classical frameworks include face, content, criterion, and construct validity, while more contemporary approaches include the frameworks proposed by Messick and Kane.^11^, ^13^, ^14^, ^15^, ^16^ Contemporary frameworks for validity provide a more comprehensive and structured approach to evaluating assessment tools, explicitly linking evidence to intended interpretations and uses. In contrast to classical frameworks, they integrate multiple sources of evidence. Messick’s framework includes content, internal structure, relationships with other variables. response processes, and consequences. Kane’s framework includes scoring, generalization, extrapolation and implications (or decisions).^11^, ^15^

A preliminary search in MEDLINE revealed no existing reviews on this topic. With this scoping review, we therefore endeavored to systematically identify all published assessment tools and evaluate the extent and nature of their reported validity evidence. In doing so, we aimed to summarize which items of neonatal resuscitation guidelines are included in these tools and to identify gaps in validity evidence. This will provide a foundation for the development of a comprehensive, up-to-date, and adequately validated assessment tool.

## Methods

This scoping review was conducted in accordance with the Joanna Briggs Institute (JBI) methodology for scoping reviews and adhered to the Preferred Reporting Items for Systematic Reviews and Meta-Analyses extension for Scoping Reviews (PRISMA-ScR).^17^, ^18^ The completed PRISMA-ScR checklist is attached in Appendix A (supplementary material). An a priori protocol was developed and registered in the Open Science Framework database (https://osf.io/).^19^

### Eligibility criteria

Inclusion: studies containing assessment tools for neonatal resuscitation that addressed basically all key components of the resuscitation algorithm were considered. Studies performed in both simulation and clinical settings were eligible. Publications in English, Dutch, German, French, Spanish, and Italian were eligible, with no restrictions regarding study design or publication date. Gray literature was not included, with the exception of obtaining the assessment tools with the accompanying validity evidence used in accredited NLS, NRP and HBB courses.

Exclusion: studies in which tools were used to assess knowledge or isolated skills were excluded. Publications were also excluded if the full-text was not available or if the assessment tool could not be retrieved.

### Search strategy

An initial limited search in MEDLINE (PubMed) and the Cochrane Library was performed to identify relevant key words and index terms. A comprehensive search strategy was drafted, including all identified key items, and adapted for all databases, with the assistance of an experienced literature specialist from the medical library of the Radboud University Medical Center, Nijmegen, the Netherlands. The Peer Review of Electronic Search Strategies (PRESS) checklist was used to optimize the search strategy (Appendix B, supplementary material).^20^ The final search was performed in MEDLINE (PubMed), Embase (OVID), and the Cochrane Library on January 20th, 2024. An updated literature search was performed on July 11th, 2025, to ensure all newly published, relevant studies were included. The full search strategies for each database are provided in Appendix C (supplementary material). Backward reference searching was not performed.

### Assessment tools used in accredited life support courses

In addition to the literature search, assessment tools, including their validity evidence, used in accredited ERC, AHA/AAP, and HBB courses were obtained by accessing their online publications and/or contacting the representatives of the ERC and NRP. For these tools, only the versions published after the year 2000 were included to capture the more recent version

### Source of evidence selection

All retrieved records were imported and deduplicated in EndNote X9 (Clarivate, Philadelphia, USA). Records were subsequently imported into Rayyan (https://rayyan.qcri.org, Oxford, UK).^21^ A pilot screening was conducted to calibrate reviewer agreement. Two reviewers (MS and LR) independently screened all titles and abstracts using the predefined inclusion and exclusion criteria. Records were classified into two categories: “clearly not eligible” and “potentially eligible”. Potentially eligible papers were independently screened full-text by the same two reviewers and classified into “not eligible” and “eligible”. Disagreements at any stage between the reviewers were resolved through discussion within the research team. All records classified as “eligible”, were included in this review.

### Data extraction, data analysis, and presentation

Two reviewers (MS and LR) independently extracted data from the included studies using three predesigned data extraction tables developed by the research team to address the review questions. The first table included (1) general study characteristics (e.g. first author, title, year of publication, country) and (2) assessment tool characteristics (e.g. associated guideline, development method, intended use). The second table listed the individual items incorporated in each assessment tool, based on the steps of the ERC, AHA/AAP, or HBB resuscitation algorithms. The third table summarized the available validity evidence for each assessment instrument and the framework (classical or contemporary) used to collect that validity evidence.^11^, ^13^, ^14^, ^15^, ^16^

Disagreements during data extraction were resolved through discussion or adjudication within the research team. Authors of the original papers were contacted to request missing or additional data, if required.

Microsoft Excel (Microsoft Corporation, Redmond, WA, USA) was used to summarize the information reported in Tables 1–3 and to perform descriptive statistics.

## Results

### Search results

**Fig. 1:**
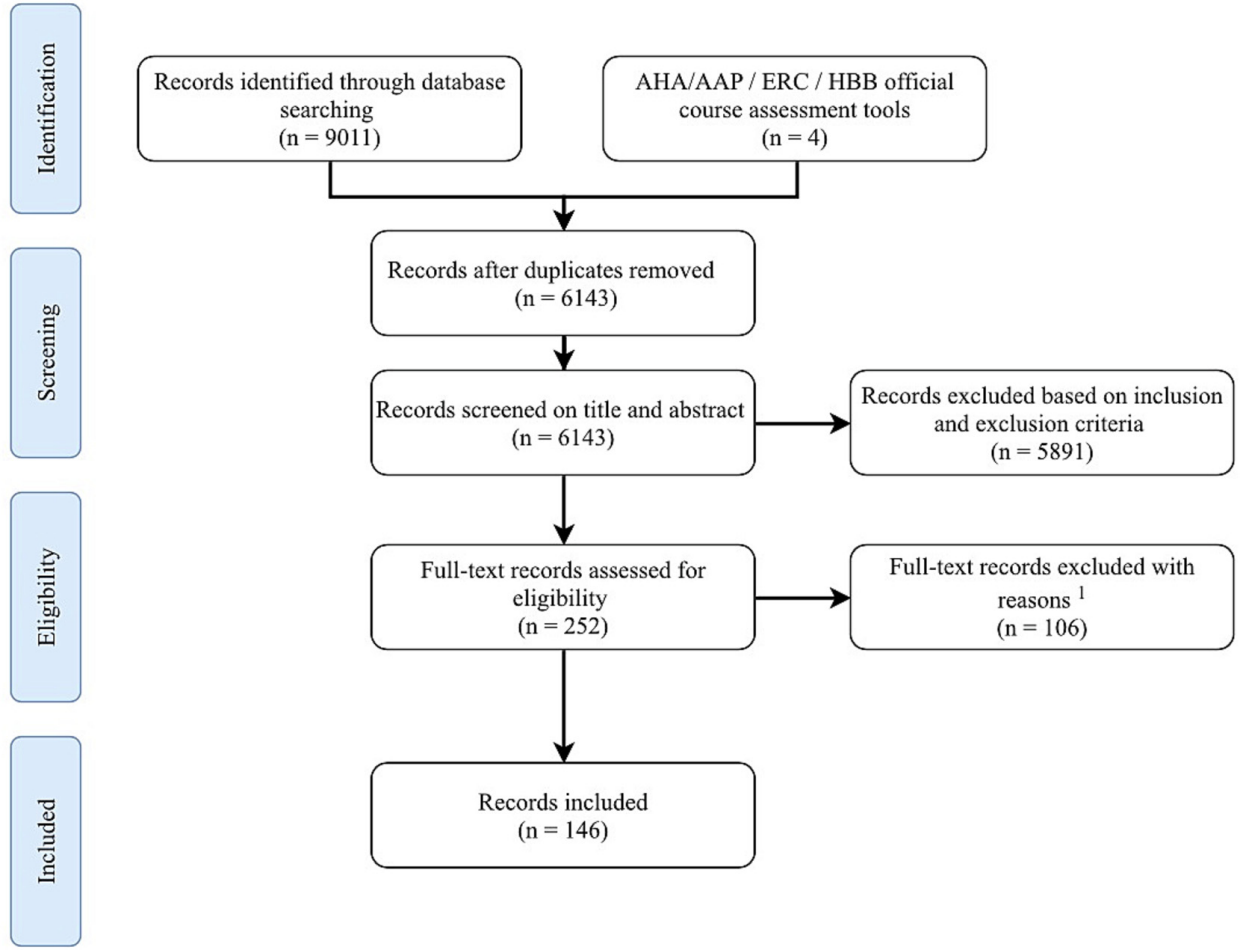
**Flowchart of the in- and exclusion process**. ^1^Reasons for exclusion are mentioned in Appendix D. *Abbreviations used in this figure*: AAP: American Academy of Pediatrics. AHA: American Heart Association. ERC: European Resuscitation Council. HBB: Helping Babies Breathe.

The search identified 9011 studies. In addition, four assessment tools used in ERC, AHA/AAP, and HBB accredited courses were gathered. A flow diagram summarizing the selection process is presented in Fig. 1. The predominant reason for exclusion was the unavailability of the full version of the article or assessment tool. Reasons for exclusion are further specified in Appendix D (supplementary material). A total of 146 sources were included, consisting of 142 publications and four assessment tools used in ERC, AHA/AAP, and HBB accredited courses (references of all included studies are listed in Appendix E, supplementary material).

### Characteristics of included studies

Across all included sources, we identified 82 unique assessment tools. These consisted of newly developed tools and substantially revised versions of existing instruments. The remaining 64 sources described previously published, unmodified assessment tools and therefore did not contribute additional information. These were excluded from further analyses, except when they provided supplementary validity evidence for the original assessment tools. Table 1 (supplementary material) shows the study characteristics and tool characteristics of all unique assessment tools. Most studies (63/82, 76.8%) were published within the last ten years. The majority were conducted in North-America and Europe (49/82, 59.8%).

### Review findings

#### Unique assessment tools

The 82 unique assessment tools included structured observation checklists and scoring instruments assessing both technical skills (e.g., ventilation, airway management, chest compressions) and non-technical skills (e.g., teamwork, decision-making). Tools varied widely in length (5–133 items, median 22, IQR 15–36). None fully incorporated all steps from AHA/AAP or ERC algorithms.

Assessment tools were mainly based on the algorithms of the AHA/AAP (43/82, 52.4%), ERC (10/82, 12.2%) and HBB (7/82, 8.5%). Eighteen tools (18/82, 22.0%) were developed for use in conjunction with other algorithms, such as those presented in national guidelines. For four assessment instruments it was unclear on which algorithm they were based.

Tools were predominantly developed for summative evaluation (69/82, 84.1%). Seven tools (7/82, 8.5%) were developed for both formative and summative evaluation, but none exclusively for formative evaluation. The remaining tools were used for other purposes (3/82, 3.7%) or their purpose was unknown (3/82, 3.7%). Tools were used for real-time assessment (24/82, 29.3%), remote video-based assessment (41/82, 50.0%) or both (*n* = 6/82, 7.3%); one study used the tool in the context of a game simulation (1.2%) and for ten tools (10/82, 12.2%) the assessment method was not mentioned. One sixth (13/82, 15.9%) of the tools were used for the clinical setting, compared to more than half (43/82, 52.4%) for simulated settings. For the other tools (26/82, 31.7%) the information about the setting was missing. Most studies (56/82, 68.3%) used a newly developed assessment tool instead of a modified version of an existing tool (24/82, 29.3%). Two tools (2/82, 2.4%) did not report the mode of tool development.

Almost half (40/82, 48.8%) of the assessment tools did not include scoring instructions. The majority (61/82, 74.4%) of the tools featured a dichotomous or trichotomous scoring system. With regard to structure and feasibility, it was noted that 59.8% (49/82) of the assessment tools were limited to one page and 53.7% (44/82) included subheadings.

A wide variation was observed in the number of items included in the assessment tools, ranging from 5 to 133 (median = 22 IQR = 15–36). The content of the assessment tools also varied substantially. None of the included assessment tools incorporated all of the items/steps mentioned in the AHA/AAP or ERC algorithms (Table 2, supplementary material). The most complete tools included 38–40 items (Table 2, supplementary material).^22^, ^23^, ^24^ Frequently included items were: “Dry the baby or place undried in a plastic bag” (73/82, 89.0%); “Start of ventilation breaths” (69/82, 84.1%) (AHA/AAP, HBB); and “Adequate airway opening manoeuvre” (67/82, 81.7%). Items that were omitted in all assessment tools were: “Titrate inspiration pressure” and “Debrief with team”. The accurate settings of the positive end expiratory pressure (PEEP) and/or peak inspiratory pressure (PIP) levels were only scored in one assessment tool, as were the items “Consider glucose bolus”, “Decision on discontinuation of resuscitation” and “Ensure adequate documentation”.

#### Validity evidence

**Fig. 2:**
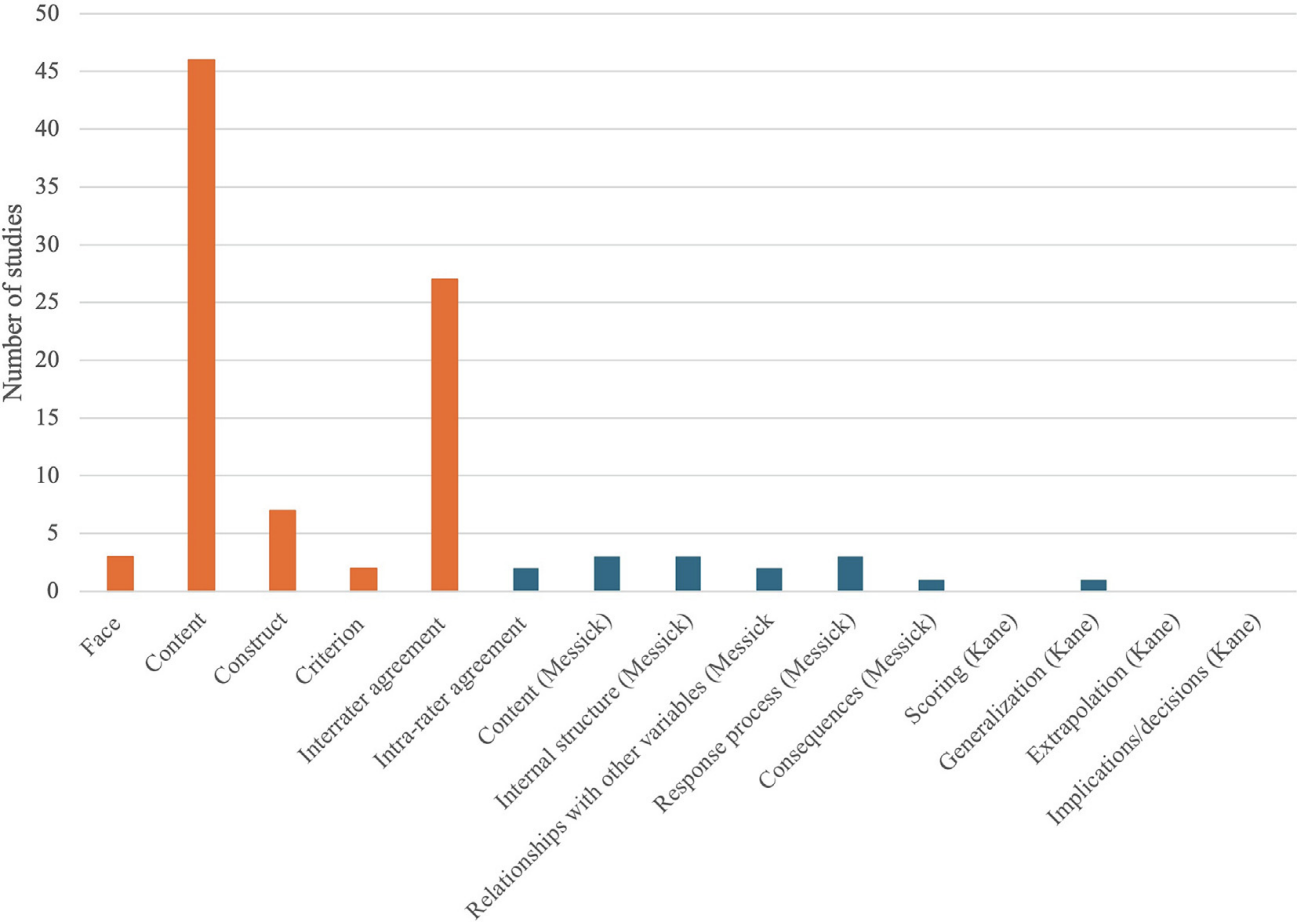
**Number of studies reporting various types of validity evidence**. *X*-axis: types of validity evidence; orange bars represent elements of classical frameworks, blue bars represent elements of contemporary frameworks. *Y*-axis: the number of studies reporting each type of validity evidence. (For interpretation of the references to color in this figure legend, the reader is referred to the web version of this article.)

Only seven tools were part of a formal validation study (7/82, 8.5%). However, some form of validity evidence was reported for 73.2% (60/82). Fig. 2 shows the number of studies reporting different types of validity evidence across classical and contemporary frameworks.

For all of these tools, the classical framework was used. Most commonly reported types of validity evidence, used in the classical framework, included content validity (46/60, 76.7%) and interrater agreement (27/60, 45.0%) (Table 3, supplementary material). Less frequently reported validity evidence were face (3/60, 5.0%), construct (7/60, 11.7%), and criterion validity (2/60, 3.3%) as well as intra-rater agreement (2/60, 3.3%). For only 18 (18/60, 30.0%) tools, more than two items of the classical framework for validity evidence were reported.

Three of these sixty assessment tools were validated using Messick’s framework.^4^, ^25^, ^26^ Yet, for only one tool, all sources of validity evidence as outlined in this framework were covered.^25^ In one of these three studies, an element of Kane’s framework, i.e. generalization, was applied in addition to Messick’s framework.^26^

Evidence related to internal structure, as defined by Messick, was reported for these three tools, whereas Cronbach’s alpha, an indicator of internal consistency, was documented for eleven tools (11/82, 13.4%) (Table 3, supplementary material).

## Discussion

To our knowledge, this is the first review identifying all published assessment tools for neonatal resuscitation and their respective validity evidence. We identified 82 unique assessment tools for neonatal resuscitation. These tools varied considerably in structure, content, and intended use. Most lacked robust validity evidence. Only a minority of the corresponding studies reported more than two elements of classical validity frameworks, and contemporary approaches such as those of Messick or Kane were rarely applied. These findings raise important concerns regarding the appropriateness and reliability of current assessment practices during simulated and actual neonatal resuscitations.

Interestingly, the assessment tools showed considerable heterogeneity in tool content, the number of items, and scoring systems. Some tools contained as little as 5 items, while others comprised 133 items. Various tools omitted critical steps such as chest compressions or preparation for resuscitation. Moreover, none of the included tools fully captured all steps as incorporated in the algorithms of international neonatal resuscitation guidelines. This variation may reflect the fact that in most articles the development of the assessment tool was not the primary objective of the study. Therefore, tools may have been tailored to study-specific aims, instead of being fully aligned with current clinical guidelines. This approach risks under-assessing essential competencies or skills, potentially compromising training outcomes and clinical safety. Assessment tools can highlight essential steps in the neonatal resuscitation algorithm (e.g. airway maneuvers and ventilatory support). This should be based on evidence on competencies most contributory to successful neonatal resuscitation, and may benefit from a Delphi procedure to uncover which skills are deemed crucial. Based on this, a differentiated scoring system can be designed in which essential skills or tasks are allocated the highest possible scores.

For most tools, the collected validity evidence was limited. When reported, it predominantly reflected classical frameworks, corroborating the validity argument with concepts as content validity and interrater reliability. These sources of validity evidence are relatively easy to acquire and therefore considered less rigorous by contemporary standards (11). Contemporary frameworks, such as those constructed by Kane and Messick, offer a more comprehensive approach by also considering the relationship with other variables, response processes, and consequences of test use. However, these were rarely applied: only three tools were validated using contemporary frameworks.^4^, ^25^, ^26^ This highlights that the advantages of contemporary frameworks, providing more comprehensive assessment and thereby supporting training and ultimately patient outcomes, are currently underutilized.

Two recent studies by Bibl et al. (2023) and Meggiolaro et al. (2025) used a Delphi process to create a comprehensive and expert-based assessment tool.^23^, ^27^ Despite this rigorous consensus method, both studies still relied on a classical framework for validity evidence, while contemporary frameworks – considered the preferred standard – were not applied.^11^, ^13^, ^14^, ^15^, ^16^ Moreover, even within the classical framework, validity evidence was assembled to a limited extent, as solely construct validity, content validity, and interrater reliability were reported.

The implications of the heterogeneity in the published assessment tools and the lack of robust validity evidence are substantial. Assessment tools serve multiple purposes: to evaluate training effectiveness, to guide feedback, to assess performance, to provide the basis for high-stakes decisions such as certification or re-licensing, to support quality assurance, and to objectively compare skills for research. Beyond summative evaluation, the formative function of these tools is equally crucial. Valid and reliable assessment procedures employing itemized instruments enable structured, personalized, and task-specific feedback that supports individual learning. As such, they serve not only as gatekeepers of competence, but also as scientific instruments for quality assurance and educational refinement.

Any HCP involved in delivery area management must be competent in neonatal resuscitation. Training, in most cases followed by an assessment, is the key to gain and maintain competence in neonatal resuscitation. Without validity evidence, there is no guarantee that assessment tools measure what they claim to assess. It remains unclear whether the existing tools can accurately distinguish between levels of competence, track learning progress, or predict clinical performance. These are essential determinants for quality of care and patient outcomes. An analogy with aviation highlights this issue clearly: a pilot may be trained, but without validated assessment, their competence cannot be assured – nor would they be allowed to fly.

This implies that there is a clear need for the development of a standardized, comprehensive, and adequately validated assessment tool that aligns with the current neonatal resuscitation algorithms. This tool should be suitable for both summative and formative assessment, across clinical and training settings. It should include all essential algorithm steps, be supported by robust validity evidence using contemporary frameworks, and allow for consistent application across training programs. In addition, it would be advisable to include more detailed scoring instructions, which could enhance interrater agreement, strengthen the response process, and support the assessment of non-technical skills that are often more difficult to evaluate. At the same time, an appropriate balance should be maintained between validity and feasibility.

A single universal tool may not be possible across the globe, due to the existence of different neonatal resuscitation algorithms, and may not fully accommodate contextual differences. However, the adoption of a shared core validity framework for neonatal resuscitation assessment tools would promote comparability, quality assurance, and global benchmarking. Moreover, as Cook et al. suggest, it may be more efficient not to develop entirely new tools, but rather to adapt existing instruments by updating, modifying, and supplementing them with additional validity evidence if needed.^11^

### Strengths and limitations

This scoping review’s main strength lies in its comprehensive and methodologically rigorous approach. We conducted a broad search across multiple databases using a peer-reviewed strategy, including all study types, and performed independent screening and data extraction. By limiting inclusion to published tools covering complete resuscitation algorithms – instead of separate skills – we focused on instruments suitable for high-stakes assessments. However, this may have excluded tools intended for narrower formative purposes. Additionally, gray literature and unpublished tools were excluded unless endorsed by major course organizations. It is possible that there are resuscitation courses using different, unpublished assessment tools that were not captured in this study. However, it is unlikely that these unpublished tools have been subjected to thorough validation processes.

## Conclusion

This scoping review provides the first comprehensive overview of existing neonatal resuscitation assessment tools and their associated validity evidence. Despite the abundance of tools, few have been rigorously validated, and none fully aligns with current clinical guidelines. Consequently, it remains unclear whether existing tools accurately measure neonatal resuscitation performance. This underscores the need for the development of a comprehensive, up-to-date, and adequately validated assessment tool for neonatal resuscitation.

## Source of funding

This research did not receive any specific grant from funding agencies in the public, commercial, or not-for-profit sectors.

## Consent

Approval by the Institutional Review Board of the Radboud University Medical Center was not necessary for this study, since human subjects were not exposed to medical activities.

## Declaration of generative AI and AI-assisted technologies in the manuscript preparation process

This manuscript was prepared without the use of generative AI or AI-assisted technologies.

## Review registration

Open Science Framework: https://doi.org/10.31219/osf.io/wsrbq.

## CRediT authorship contribution statement

**Maud Steins:** Writing – review & editing, Writing – original draft, Methodology, Investigation, Formal analysis, Data curation, Conceptualization. **Lieke Raaijmakers:** Writing – review & editing, Writing – original draft, Methodology, Investigation, Formal analysis, Data curation, Conceptualization. **Marije Hogeveen:** Writing – review & editing, Supervision, Methodology, Conceptualization. **Mathijs Binkhorst:** Writing – review & editing, Supervision, Methodology, Conceptualization.

## Declaration of competing interest

The authors declare the following financial interests/personal relationships which may be considered as potential competing interests: Marije Hogeveen and Mathijs Binkhorst are chair and member, respectively, of the Science and Education Committee (SEC) for Neonatal Life Support (NLS) of the European Resuscitation Council (ERC). Marije Hogeveen and Mathijs Binkhorst were chair and member of the NLS writing group for the 2025 ERC NLS guideline. Marije Hogeveen is national course director and instructor of the Newborn (Advanced) Life Support course, member of the board of the Dutch Foundation for the Emergency Medical Care of Children, and member of the scientific board of the Dutch Resuscitation Council. The other authors declare no conflicts of interest. If there are other authors, they declare that they have no known competing financial interests or personal relationships that could have appeared to influence the work reported in this paper.

## Data Availability

The data that support the findings of this study are available from the corresponding author on reasonable request. Data are stored in a confidential and secure folder at the Radboud University Medical Center Amalia Children’s Hospital.
