# Supplementary material for: Assessment tools for neonatal resuscitation and their validity evidence: a scoping review
Source: Resusc Plus. 2026 Mar 25;29:101304. doi: 10.1016/j.resplu.2026.101304 (PMC13090731; doi:10.1016/j.resplu.2026.101304)
Supplement: Supplementary Data 1 [file mmc1.docx]

**Table of content**

1. Appendix A: PRISMA-ScR checklist pages 2 - 3
2. Appendix B: PRESS 2015 Guideline Evidence-Based pages 4 - 5
   Checklist
3. Appendix C: Search strategy pages 6 - 7
4. Appendix D: Reasons for exclusion of full text pages 8 - 14
   articles
5. Appendix E: References of all included studies pages 15 - 21
6. Table 1: Study and assessment tool characteristics pages 22 - 25
7. Table 2: Overview of assessment tool items mapped pages 26 - 35
   to the ERC and AHA resuscitation algorithms
8. Table 3: Summary of the validity evidence of all pages 36 - 37
   included studies

**Appendix A: PRISMA-ScR checklist**

Preferred Reporting Items for Systematic reviews and Meta-Analyses extension for Scoping Reviews (PRISMA-ScR) Checklist

| SECTION | ITEM | PRISMA-ScR CHECKLIST ITEM | REPORTED ON PAGE # |
| --- | --- | --- | --- |
| **TITLE** | | | |
| Title | 1 | Identify the report as a scoping review. | 3 |
| **ABSTRACT** | | | |
| Structured summary | 2 | Provide a structured summary that includes (as applicable): background, objectives, eligibility criteria, sources of evidence, charting methods, results, and conclusions that relate to the review questions and objectives. | 6 |
| **INTRODUCTION** | | | |
| Rationale | 3 | Describe the rationale for the review in the context of what is already known. Explain why the review questions/objectives lend themselves to a scoping review approach. | .8-10 |
| Objectives | 4 | Provide an explicit statement of the questions and objectives being addressed with reference to their key elements (e.g., population or participants, concepts, and context) or other relevant key elements used to conceptualize the review questions and/or objectives. | 9-10 |
| **METHODS** | | | |
| Protocol and registration | 5 | Indicate whether a review protocol exists; state if and where it can be accessed (e.g., a Web address); and if available, provide registration information, including the registration number. | 11 |
| Eligibility criteria | 6 | Specify characteristics of the sources of evidence used as eligibility criteria (e.g., years considered, language, and publication status), and provide a rationale. | 11 |
| Information sources* | 7 | Describe all information sources in the search (e.g., databases with dates of coverage and contact with authors to identify additional sources), as well as the date the most recent search was executed. | 12 |
| Search | 8 | Present the full electronic search strategy for at least 1 database, including any limits used, such that it could be repeated. | Appendix III |
| Selection of sources of evidence† | 9 | State the process for selecting sources of evidence (i.e., screening and eligibility) included in the scoping review. | 12 |
| Data charting process‡ | 10 | Describe the methods of charting data from the included sources of evidence (e.g., calibrated forms or forms that have been tested by the team before their use, and whether data charting was done independently or in duplicate) and any processes for obtaining and confirming data from investigators. | 12-13 |
| Data items | 11 | List and define all variables for which data were sought and any assumptions and simplifications made. | 13 |
| Critical appraisal of individual sources of evidence§ | 12 | If done, provide a rationale for conducting a critical appraisal of included sources of evidence; describe the methods used and how this information was used in any data synthesis (if appropriate). | Not applicable |
| Synthesis of results | 13 | Describe the methods of handling and summarizing the data that were charted. | 13 |
| **RESULTS** | | | |
| Selection of sources of evidence | 14 | Give numbers of sources of evidence screened, assessed for eligibility, and included in the review, with reasons for exclusions at each stage, ideally using a flow diagram. | Figure I  Appendix IV |
| Characteristics of sources of evidence | 15 | For each source of evidence, present characteristics for which data were charted and provide the citations. | 14 |
| Critical appraisal within sources of evidence | 16 | If done, present data on critical appraisal of included sources of evidence (see item 12). | Not applicable |
| Results of individual sources of evidence | 17 | For each included source of evidence, present the relevant data that were charted that relate to the review questions and objectives. | 14-16 |
| Synthesis of results | 18 | Summarize and/or present the charting results as they relate to the review questions and objectives. | 14-16 |
|  | | | |
| Summary of evidence | 19 | Summarize the main results (including an overview of concepts, themes, and types of evidence available), link to the review questions and objectives, and consider the relevance to key groups. | 17-19 |
| Limitations | 20 | Discuss the limitations of the scoping review process. | 19-20 |
| Conclusions | 21 | Provide a general interpretation of the results with respect to the review questions and objectives, as well as potential implications and/or next steps. | 20 |
| **FUNDING** | | | |
| Funding | 22 | Describe sources of funding for the included sources of evidence, as well as sources of funding for the scoping review. Describe the role of the funders of the scoping review. | 4 |

JBI = Joanna Briggs Institute; PRISMA-ScR = Preferred Reporting Items for Systematic reviews and Meta-Analyses extension for Scoping Reviews.

* Where *sources of evidence* (see second footnote) are compiled from, such as bibliographic databases, social media platforms, and Web sites.

† A more inclusive/heterogeneous term used to account for the different types of evidence or data sources (e.g., quantitative and/or qualitative research, expert opinion, and policy documents) that may be eligible in a scoping review as opposed to only studies. This is not to be confused with *information sources* (see first footnote).

‡ The frameworks by Arksey and O’Malley (6) and Levac and colleagues (7) and the JBI guidance (4, 5) refer to the process of data extraction in a scoping review as data charting*.*

§ The process of systematically examining research evidence to assess its validity, results, and relevance before using it to inform a decision. This term is used for items 12 and 16 instead of "risk of bias" (which is more applicable to systematic reviews of interventions) to include and acknowledge the various sources of evidence that may be used in a scoping review (e.g., quantitative and/or qualitative research, expert opinion, and policy document).

*From:* Tricco AC, Lillie E, Zarin W, O'Brien KK, Colquhoun H, Levac D, et al. PRISMA Extension for Scoping Reviews (PRISMAScR): Checklist and Explanation. Ann Intern Med. 2018;169:467–473. [doi: 10.7326/M18-0850](http://annals.org/aim/fullarticle/2700389/prisma-extension-scoping-reviews-prisma-scr-checklist-explanation).

**Appendix B: PRESS 2015 Guideline Evidence-Based Checklist**

Translation of the research question
1. Does the search strategy match the research question/PICO? *A PICO was not applicable for this research question. But we formulated the research question clearly and the search strategy matches this question.*
2. Are the search concepts clear? *Yes.*
3. Are there too many or too few PICO elements included? *No, for our search strategy we decided to use three different “main topics”/”search boxes” derived from our research question and combine these with AND. These topics include: neonates / neonatal intensive care, resuscitation and assessment.*3. Are the search concepts too narrow or too broad? *We believe both topic 1 “neonates / neonatal intensive care” and topic 2 “resuscitation” are neither too broad or too narrow as these topics comprise two general populations/concepts. All added search terms are relevant for our research question. Topic 3 “assessment”, is broad, as it comprises a broad concept. We critically reviewed which terms were necessary to include and which could be excluded, by trying out different search terms and appraising the impact on the number of found articles. We also roughly screened the relevance of the “missed articles” by excluding the different search terms.*

4. Does the search retrieve too many or too few records? (Please show number of hits per line.)

*Number of hits per line in the PubMed database:*

| *#3* | *Search: "Clinical Competence"[Mesh] OR "Educational Measurement"[Mesh:NoExp] OR Assessment* [tiab] OR Clinical competence*[tiab] OR Clinical skill*[tiab] OR Educational Measurement*[tiab] OR Scoring* [tiab] OR Score* [tiab] OR Standardized scenario*[tiab] OR Clinical Exam*[tiab] OR OSCE [tiab]* | *2,714,297* |
| --- | --- | --- |
| *#2* | *Search: "Resuscitation"[Mesh] OR CPR[tiab] OR Neonatal advanced life support [tiab] OR Neonatal life support[tiab] OR Newborn advanced life support [tiab] OR Newborn life support[tiab] OR Resuscitat* [tiab] OR Reanimation [tiab] OR NRP[tiab] OR NLS [tiab]* | *165,680* |
| *#1* | *Search: "Infant, Newborn"[Mesh] OR "Intensive Care, Neonatal"[Mesh] OR "Intensive Care Units, Neonatal"[Mesh] OR newborn* [tiab] OR neonat* [tiab] OR prematur* [tiab] OR preterm* [tiab] OR Newly born [tiab] OR NICU[tiab] OR NICUs[tiab]* | *1,026,865* |

*“search boxes” #1 and #3 retrieve many records. However, by combining all boxes with AND, we believe we do not have too many or too few records (n = 3569).*

5. Are unconventional or complex strategies explained? *No, this search does not have unconventional or complex strategies.*

Boolean and proximity operators (these vary based on search service)
1. Are Boolean or proximity operators used correctly? *Yes.*2. Is the use of nesting with brackets appropriate and effective for the search? *Yes.*
3. If NOT is used, is this likely to result in any unintended exclusions? *NOT is not used.*
4. Could precision be improved by using proximity operators (eg, adjacent, near, within) or phrase searching instead of AND? *No.*
5. Is the width of proximity operators suitable (eg, might adj5 pick up more variants than adj2)? *Yes.*

Subject headings (database specific)
1. Are the subject headings relevant? *Yes.*
2. Are any relevant subject headings missing; for example, previous index terms? *No.*
3. Are any subject headings too broad or too narrow? *A few subject headings are broad, however we believe necessary as these comprise the important topics in our research question.*
4. Are subject headings exploded where necessary and vice versa? *Yes.*
5. Are major headings (‘‘starring’’ or restrict to focus) used? If so, is there adequate justification? *No.*
6. Are subheadings missing? *No.*
7. Are subheadings attached to subject headings? (Floating subheadings may be preferred.) *No.*
8. Are floating subheadings relevant and used appropriately? *They are not used.*
9. Are both subject headings and terms in free text (see the following) used for each concept? *Yes.*

Text word searching (free text)
1. Does the search include all spelling variants in free text (eg, UK vs. US spelling)? *Yes.*2. Does the search include all synonyms or antonyms (eg, opposites)? *Yes, for example we used neonate / newborn and preterm / premature.*
3. Does the search capture relevant truncation (ie, is truncation at the correct place)? *Yes.*
4. Is the truncation too broad or too narrow? *No.*
5. Are acronyms or abbreviations used appropriately? Do they capture irrelevant material? *Yes, for example we added NLS and NRP which are abbreviations. This could lead to capturing irrelevant material as these are also abbreviations for other (irrelevant) terms. However, combined with the other parts of the search strategy they don’t capture irrelevant material.*
6. Are the full terms also included? *Yes.*
7. Are the keywords specific enough or too broad? Are too many or too few keywords used? Are stop words used? *No.*
8. Have the appropriate fields been searched; for example, is the choice of the text word fields (.tw.) or all fields (.af.) appropriate? Are there any other fields to be included or excluded (database specific)? *Yes.*
9. Should any long strings be broken into several shorter search statements? *No.*

Spelling, syntax, and line numbers
1. Are there any spelling errors? *No.*
2. Are there any errors in system syntax; for example, the use of a truncation symbol from a different search interface? *No.*
3. Are there incorrect line combinations or orphan lines (ie, lines that are not referred to in the final summation that could indicate an error in an AND or OR statement)? *No.*

Limits and filters
1. Are all limits and filters used appropriately and are they relevant given the research question? *No limits or filters are used.*
2. Are all limits and filters used appropriately and are they relevant for the database? *No limits or filters are used.*
3. Are any potentially helpful limits or filters missing? Are the limits or filters too broad or too narrow? Can any limits or filters be added or taken away? *No limits or filters are used. For example, we decided not to use the filter “Publication Date” as we also wanted to retrieve information on outdated NLS/NRP guidelines. As we believe these could also capture relevant aspects.*
4. Are sources cited for the filters used? *No, as no filters are used.*

**Appendix C: Search strategy**

MEDLINE (PubMed) search strategy:

"Infant, Newborn"[Mesh] OR "Intensive Care, Neonatal"[Mesh] OR "Intensive Care Units, Neonatal"[Mesh] OR newborn* [tiab] OR neonat* [tiab] OR prematur* [tiab] OR preterm* [tiab] OR Newly born [tiab] OR NICU[tiab] OR NICUs[tiab]

AND

"Resuscitation"[Mesh] OR CPR[tiab] OR Neonatal advanced life support [tiab] OR

Neonatal life support[tiab] OR Newborn advanced life support [tiab] OR Newborn life support[tiab] OR Resuscitat* [tiab] OR Reanimation [tiab] OR NRP[tiab] OR NLS [tiab]

AND

"Clinical Competence"[Mesh] OR "Educational Measurement"[Mesh:NoExp] OR

Assessment* [tiab] OR Clinical competence*[tiab] OR Clinical skill*[tiab] OR

Educational Measurement*[tiab] OR Scoring* [tiab] OR Score* [tiab] OR

Standardized scenario*[tiab] OR Clinical Exam*[tiab] OR OSCE [tiab]

Cochrane search strategy:

[mh “Infant, Newborn”] OR [mh “Intensive Care, Neonatal”] OR [mh “Intensive Care Units, Neonatal”] OR (newborn* OR neonat* OR prematur* OR preterm* OR newly born OR NICU OR NICUs):ti,ab,kw

AND

[mh Resuscitation] OR (CPR OR Neonatal advanced life support OR Neonatal life support OR Newborn advanced life support OR Newborn life support OR Resuscitat* OR Reanimation OR NRP OR NLS):ti,ab,kw

AND

[mh “Clinical Competence”] OR [mh ^”Educational Measurement”] OR
(Assessment* OR Clinical competence* OR Clinical skill* OR Educational Measurement* OR Scoring* OR Score* OR Standardized scenario* OR Clinical Exam* OR OSCE):ti,ab,kw

Embase search strategy:

newborn/ OR exp newborn disease/ OR exp newborn care/ OR neonatal intensive care unit/ OR newborn.ti,ab,kf. OR Neonat*.ti,ab,kf. OR Premature*.ti,ab,kf. OR Preterm.ti,ab,kf. OR

Newly born.ti,ab,kf. OR NICU.ti,ab,kf. OR NICUs.ti,ab,kf.

AND

exp resuscitation/ OR neonatal life support.ab,kf,ti. OR Neonatal advanced life support.ab,kf,ti. OR Newborn advanced life support.ab,kf,ti. OR Newborn life support.ab,kf,ti. OR Resuscitat*.ti,ab,kf. OR Reanimation.ti,ab,kf. OR NRP.ab,kf,ti. OR NLS.ab,kf,ti.

AND

clinical competence/ OR clinical assessment tool/ OR assessment*.ab,kf,ti. OR

Clinical competence*.ab,kf,ti. OR Clinical skill*.ab,kf,ti. OR Educational Measurement*.ab,kf,ti. OR score*.ab,kf,ti. OR scoring*.ab,kf,ti. OR Standardized scenario*.ab,kf,ti. OR Clinical exam*.ab,kf,ti. OR OSCE.ab,kf,ti.

Added filter: conference abstracts were excluded.

**Appendix D: Reason for exclusion of full text articles.**

**First search (January 2024)**

|  | First authors (year) | Title | Reason for exclusion |
| --- | --- | --- | --- |
| 1 | Abou-Zamzam (2023) | Cross-sectional study in Madagascar demonstrates efficacy of virtual mentoring and flipped classroom modifications of neonatal resuscitation programme Helping Babies Breathe | Assessment tool not available |
| 2 | Agrawal (2020) | Competency based Training & Evaluation of Final Year MBBS Students on Neonatal Resuscitation | Assessment tool not available |
| 3 | Amin (2018) | Use of simulation-based skill acquisition instruction (SSAI) for manual mask ventilation (MMV) improves the ability of neonatal resuscitation program (NRP) providers to deliver and retain effective MMV skills | No full text |
| 4 | Arghavanian (2023) | Comparison of Structured Clinical Instruction Module and Video-Assisted Instructor -Led Training in Accelerating and Retention of Nursing and Midwifery Students’ Skill in Neonatal Resuscitation | Assessment tool not available |
| 5 | Bala (2023) | Effectiveness of Simulation Versus Video Instruction on Neonatal Resuscitation: A Randomized Control Trial | Assessment tool not available |
| 6 | Barré (2020) | Midwifery students’ retention of learning after  screen-based simulation training on neonatal  resuscitation: a pilot study | Assessment tool not available |
| 7 | Bhat (1993) | Undergraduate training in neonatal resuscitation -- a modified approach | No full text |
| 8 | Bhatia (1993) | Training of final year MBBS students in neonatal resuscitation | No full text |
| 9 | Bhatt (2023) | A Pilot Study on Testing Real Life Delivery Room Experience by Direct Observation and Supervised "Hands-on" Component in Imparting Neonatal Resuscitation Skill to Intern Doctor | Assessment tool not available |
| 10 | Bookman (2010) | Educational impact of a hospital-based neonatal resuscitation program in Ghana | Assessment tool not available |
| 11 | Bould (2009) | A cognitive aid for neonatal resuscitation: a randomized controlled trial | Duplicate |
| 12 | Briggs (2021) | Basic neonatal resuscitation: retention of knowledge  and skills of primary health care workers in Port  Harcourt, Rivers State, Southern Nigeria | Assessment tool not available |
| 13 | Bruno (2016) | Simulation as a tool for improving acquisition of neonatal resuscitation skills for obstetric residents | No assessment tool mentioned |
| 14 | Campbell (2014) | Impact of video-debriefing following simulated neonatal resuscitation in inter-professional teams | No full text |
| 15 | Cepeda Brito (2017) | Neonatal Resuscitation Program Rolling Refresher: Maintaining Chest Compression Proficiency Through the Use of Simulation-Based Education | No full text |
| 16 | Conroy (2014) | Neonatal resuscitation skills amongst healthcare workers in Bo district, Sierra Leone | No full text |
| 17 | Conroy (2015) | Skills retention 3 months after neonatal resuscitation training in a cohort of healthcare workers in Sierra Leone | No assessment tool mentioned |
| 18 | Curran (2004) | Evaluation of the Effect of a Computerized Training  Simulator (ANAKIN) on the Retention of Neonatal  Resuscitation Skills | Assessment tool not available |
| 19 | Curran (2005) | A comparison of face-to-face versus remote  assessment of neonatal resuscitation skills | Assessment tool not available |
| 20 | De Graft-Johnson (2017) | Cross-sectional observational assessment of quality of newborn care immediately after birth in health facilities across six sub-Saharan African countries | No assessment tool mentioned |
| 21 | Del Hierro (2017) | An improvement in medical undergraduate education in Ecuador throught hands-on training in perinatal skills | No assessment tool mentioned |
| 22 | Devi (2019) | Effect of Flipped Classroom Teaching on Improvement of Clinical Performance in Labour Room among Nursing Students | Assessment tool not available |
| 23 | Dinur (2021) | Evaluation of audio-voice guided application for neonatal resuscitation: a prospective, randomized, pilot study | No assessment tool mentioned |
| 24 | Dogne (2024) | Assessment of Skills of Health Care Professionals Posted in Labour Room and Maternity OTs Based on OSCE (Objective Structured Clinical Examination) Using LaQshya Guidelines | Wrong topic (not about neonatal resuscitation) |
| 25 | Ersdal (2013) | A one-day "Helping Babies Breathe" course improves simulated performance but not clinical management of neonates | No assessment tool mentioned |
| 26 | Evans (2018) | Onsite simulation training to improve care and save lives in Uganda: a cluster randomized trial | No full text |
| 27 | Felton (2023) | Using Simulation-Based Training to Improve Neonatal Resuscitation Clinical Competency, Confidence, and Comfort Level of NICU Caregivers | No full text |
| 28 | Fuerch (2015) | Impact of a novel decision support tool on adherence to Neonatal Resuscitation Program algorithm | Wrong concept |
| 29 | Gebreegziabher (2014) | Knowledge and skills of neonatal resuscitation of health professionals at a university teaching hospital of Northwest Ethiopia | No assessment tool mentioned |
| 30 | Ghorbandoost (2018) | Evaluating the effectiveness of neonatal resuscitation training course on nurses of Kowsar medical center in Qazvin University of medical sciences based on Kirkpatrick model | No full text |
| 31 | Gomez (2018) | Accelerating newborn survival in Ghana through a low-dose, high-frequency health worker training approach: a cluster randomized trial | Assessment tool not available |
| 32 | Gunay (2013) | Resuscitation skills of pediatric residents and effects of Neonatal Resuscitation Program training | Assessment tool not available |
| 33 | Hakimi (2021) | Investigating the effect of neonatal resuscitation  simulation using a competency‐based approach on  knowledge, skill, and self‐confidence of midwifery  students using objective structured clinical examination (OSCE) | Assessment tool not available |
| 34 | Harvey (2007) | Are skilled birth attendants really skilled? A measurement method, some disturbing results and a potential way forward | Assessment tool not available |
| 35 | Higgins (2020) | Cohort study of neonatal resuscitation skill retention in frontline healthcare facilities in Bihar, India, after PRONTO simulation training | No assessment tool mentioned |
| 36 | Hirst (2009) | Application of evidence-based teaching in maternal and child health in remote Vietnam | No assessment tool mentioned |
| 37 | Hosokawa (2011) | Impact of neonatal resuscitation training workshop in Mongolia | No full text |
| 38 | Jabir (2009) | Knowledge and practical performance gained by Iraqi residents after participation to a neonatal resuscitation program course | Assessment tool not available |
| 39 | Jain (2010) | Tele-education vs classroom training of neonatal resuscitation: a randomized trial | No assessment tool mentioned |
| 40 | Jeffery (1996) | Competency-based learning in neonatology | No assessment tool mentioned |
| 41 | Kaczorowski (1998) | Retention of neonatal resuscitation skills and knowledge: a randomized controlled trial | No full text |
| 42 | Kalaniti (2019) | Simulation-based team-leadership training for neonatal resuscitation: is learning by observation as effective as learning by participation? | No full text |
| 43 | Karakoc (2019) | The effects of simulation-based education on initial neonatal evaluation and care skills | Wrong topic |
| 44 | Kibwana (2016) | Preparing the Health Workforce in Ethiopia:  A Cross‐sectional Study of Competence of Anesthesia Graduating Students | Assessment tool not available |
| 45 | Kudreviciene (2019) | Initial neonatal resuscitation: skill retention after  the implementation of the novel 24/7 HybridLab®  learning system | Assessment tool not available |
| 46 | Law (2018) | Effect of monitor placement on situational awareness and visual attention in simulated neonatal resuscitations | No full text |
| 47 | Law (2020) | Effect of monitor positioning on visual attention and situation awareness during neonatal resuscitation: a randomised simulation study | Assessment tool not available |
| 48 | Leeman (2023) | Development and use of an infant resuscitation performance tool (Infa-RePT) to improve team performance | Wrong topic |
| 49 | Leonard (2010) | Expert modeling improves the retention of technical skills in neonatal resucitation training | No full text |
| 50 | Lim (2023) | Sustained Effect of Simulation-Based Resuscitation Education on Knowledge, Self-Confidence, and Performance Ability of Neonatal Intensive Care Unit Nurses | No full text |
| 51 | Lu (2020) | Unsupervised Machine Learning  Algorithms Examine Healthcare  Providers’ Perceptions and  Longitudinal Performance in a Digital  Neonatal Resuscitation Simulator | No assessment tool mentioned |
| 52 | Lund (2016) | Association Between the Safe Delivery App and Quality of Care and Perinatal Survival in Ethiopia  A Randomized Clinical Trial | Assessment tool not available |
| 53 | Manzar (2004) | Use of structured question format in neonatal resuscitation assessment | No full text |
| 54 | Martes Gomes (2023) | Use of electronic text messaging of NRP principles to pediatric trainees & impact on retention | No full text |
| 55 | Mathai (2015) | Comparison of training in neonatal resuscitation using self inflating bag and T-piece resuscitator | Wrong topic |
| 56 | Mavis (2021) | Training fellows in neonatal tele-resuscitation using a simulation-based mastery learning model | Wrong topic |
| 57 | McDermott (2001) | Two models of in-service training to improve midwifery skills: how well do they work? | No assessment tool available |
| 58 | Mildenberger (2017) | Neonatal resuscitation training for midwives in Uganda: Strengthening skill and knowledge retention | No assessment tool mentioned |
| 59 | Monebenimp (2012) | Competence of health care providers on care of newborns at birth in a level-1 health  facility in Yaoundé, Cameroon | Assessment tool not available |
| 60 | Mosley (2013) | A longitudinal cohort study to investigate the  retention of knowledge and skills following  attendance on the Newborn Life support course | Assessment tool not available |
| 61 | Nishimwe (2021) | The effect of an mLearning application on nurses' and midwives' knowledge and skills for the management of postpartum hemorrhage and neonatal resuscitation: pre-post intervention study | No assessment tool mentioned |
| 62 | Neupane (2022) | Simulation Based vs Conventional Training for Initial Steps in Delivery  Room Care of Preterm Neonates: An Open Label Randomized Trial | Assessment tool not available |
| 63 | Ortiz-Movilla (2022) | Combined application of various quality assessment  tools in neonatal resuscitation. | Wrong concept |
| 64 | Rao (2019) | Where there is no nurse: an observational study of large-scale mentoring of auxiliary nurses to  improve quality of care during childbirth at primary health centres in India | Assessment tool not available |
| 65 | Rubio Gurung (2014) | In Situ Simulation Training for Neonatal Resuscitation: An RCT | Assessment tool not available |
| 66 | Skidmore (2001) | Retention of skills in neonatal  resuscitation | Assessment tool not available |
| 67 | Surcouf (2012) | Enhancing residents’ neonatal resuscitation competency through unannounced simulation-based  training | Assessment tool not available |
| 68 | Szyld (2020) | Self-directed video versus instructor-based neonatal resuscitation training: a randomized controlled blinded non-inferiority multicenter international study | Duplicate |
| 69 | Szyld (2021) | Self-directed video versus instructor-based neonatal resuscitation training: a randomized controlled blinded non-inferiority multicenter international study | Duplicate |
| 70 | Tang (2016) | Improvement and retention of emergency obstetrics and neonatal care knowledge and skills in a hospital mentorship program in Lilongwe, Malawi | No assessment tool mentioned |
| 71 | Umoren (2021) | Ehbb: a randomized controlled trial of virtual reality for newborn resuscitation refresher training of healthcare workers in Nigeria and Kenya | No full text |
| 72 | Umoren (2020) | Pre-Course Preparation with eSim™ Computer-based Simulation Improves Neonatal Provider Performance on Standardized Simulations | No full text |
| 73 | Van Der Heide (2008) | Neonatal resuscitation skills of pediatric residents. [Dutch] | No full text |
| 74 | Woldman (2017) | MedNav and Neonatal Life Support Resuscitation Learning: a randomised control trial at 0 and 7-week follow-up | No full text |
| 75 | Yaregal (2022) | Simulation-Based Neonatal Resuscitation Education for UndergraduateAnesthesia Students:A Pre- andPost-Evaluation of  Knowledge and Clinical Skills | Assessment tool not available |
| 76 | Young (2022) | Assessing provider performance of intrapartum care using stimulated encounters and clinical vignettes: A comparison study from Tanzania | Assessment tool not available |
| 77 | Zanno (2022) | Simulation-Based Outreach Program Improves  Rural Hospitals’ Team Confidence in Neonatal  Resuscitation | Assessment tool not available |

**Second search (July 2025)**

| 1 | Ali | Development of educational modules for resuscitation of neonates in the delivery room with congenital heart disease (learn-chdstudy) | No full text |
| --- | --- | --- | --- |
| 2 | Bardelli (2024) | Effect of continuous workshop training of the helping babies breathe  program on the retention of midwives’ knowledge and skills: A clinical trial study | No assessment tool mentioned |
| 3 | Chan | NRP PROMPT: a RANDOMIZED CONTROLLED TRIAL OF A MOBILE APP FOR NEONATAL RESUSCITATION TRAINING | No full text |
| 4 | Chae (2024) | Effectiveness of simulation-based  interprofessional education on teamwork  and communication skills in neonatal  resuscitation | Assessment tool not available |
| 5 | Garcia-Flores (2024) | Evaluación de las competencias para la reanimación  neonatal en personal de salud de México | Assessment tool not available |
| 6 | Cutumisu (2024) | The Effects of a Digital Game Simulator versus a Traditional Intervention on Paramedics' Neonatal Resuscitation Performance | No assessment tool mentioned |
| 7 | Emmanuel (2025) | The impact of educational interventions on the competence of nurses and midwives in neonatal resuscitation in sub-Saharan Africa: A systematic review | No assessment tool mentioned |
| 8 | Felton (2024) | Using Simulation-Based Training to Improve Neonatal Resuscitation Clinical Competency, Confidence, and Comfort Level of NICU Caregivers | No full text |
| 9 | Gentle (2025) | Reducing adverse delivery outcomes through teleneonatology: a randomized simulation trial | No full text |
| 10 | Haynes (2024) | How Much Training Is Enough? Low-Dose, High-Frequency Simulation Training and Maintenance of Competence in Neonatal Resuscitation | No assessment tool mentioned |
| 11 | Isohoso (2025) | Preclinical usability evaluation of the Liveborn app: A mobile health application that provides feedback for neonatal resuscitation | No assessment tool mentioned |
| 12 | Kain (2024) | Qualitative Insights Into Enhancing Neonatal Resuscitation in Post-Pandemic Vietnam: A Stakeholder Perspective on the Helping Babies Breathe Program | No assesment tool mentioned |
| 13 | Lim (2024) | Sustained Effect of Simulation-Based Resuscitation Education on Knowledge, Self-Confidence, and Performance Ability of Neonatal Intensive Care Unit Nurses | No full text |
| 14 | Melendi (2025) | Development and Evaluation of a Rural Longitudinal Neonatal Resuscitation Program Telesimulation Program (MOOSE: Maine Ongoing Outreach Simulation Education) | No full text |
| 15 | Mihreti (2024) | Knowledge and skills of newborn resuscitation among health care professionals in East Africa. A systematic review and meta-analysis | No assessment tool mentioned |
| 16 | Mileder (2024) | Impact of in situ simulation training on quality of postnatal stabilization and resuscitation-a before-and-after, non-controlled quality improvement study | No assessment tool mentioned |
| 17 | Mrutu (2025) | Evaluation Of Neonatal Resuscitation Practice and Associated Factors Among Nurses Located In The Delivery Suites At Muhimbili National Hospital, Dar Es Salaam-Tanzania | No full text |
| 18 | Nelin (2024) | Does the Use of an Automated Resuscitation Recorder Improve Adherence to NRP Algorithms and Code Documentation? | Assessment tool not available |
| 19 | Owusu (2024) | Evaluating the Effectiveness of an Evidence-Based Practice in Neonatal Resuscitation among Birth Asphyxiated Newborns in a Developing Country | No assessment tool mentioned |
| 20 | Pactr | Training Midwives Regarding Newborn Resuscitation on Neonatal Outcomes: a Randomized Clinical Trial Study | No full text |
| 21 | Park (2025) | 360° Camera Versus Two-View Standard Video Camera Capture of Neonatal Resuscitation: Optimizing Recording for Observation of Resuscitation Steps | Wrong topic |
| 22 | Rozycki (2025) | Assessing newborn scoring with each resuscitation (ANSWER): Protocol for identifying and testing an Apgar score for the 21st century | Wrong topic |
| 23 | Snarr (2024) | Improving Neonatal Resuscitation Knowledge in Advanced Providers Through Simulation: A Quality Improvement Project | Wrong topic |
| 24 | Soghikian (2024) | Assessing Team Performance in a Longitudinal Neonatal Resuscitation Simulation Training Program: Comparing Validity Evidence to Select the Best Tool | Wrong topic |
| 25 | Teh (2024) | Evaluation of the retention of knowledge, skills and competency of post-neonatal resuscitation training among house officers | No assessment tool mentioned |
| 26 | Trinh (2025) | A pilot study of a virtual reality-based simulation platform for Neonatal Resuscitation Program training | No assessment tool mentioned |
| 27 | Umoren (2025) | Impact of virtual simulation vs. Video refresher training on NRP simulation performance: a randomized controlled trial | Assessment tool not available |
| 28 | Van Tetering (2021) | Evaluating the Instructional Design and Effect on Knowledge, Teamwork, and Skills of Technology-Enhanced Simulation-Based Training in Obstetrics in Uganda: stepped-Wedge Cluster Randomized Trial | Wrong topic |
| 29 | Vogel (2021) | Augmented reality simulation-based training for midwifery students and its impact on perceived knowledge, confidence and skills for managing critical incidents | No assessment tool mentioned |

**Appendix E: References of all included studies**

[1] Madar J, Roehr CC, Ainsworth S, Ersdal H, Morley C, Rüdiger M, et al. European Resuscitation Council Guidelines 2021: Newborn resuscitation and support of transition of infants at birth. Resuscitation. 2021;161:291-326. <https://doi.org/10.1016/j.resuscitation.2021.02.014>.

[2] Kamath-Rayne BD, Thukral A, Visick MK, Schoen E, Amick E, Deorari A, et al. Helping Babies Breathe, Second Edition: A model for strengthening educational programs to increase global newborn survival. Glob Health Sci Pract. 2018;6(3):402-15.

[3] Kattwinkel J, Aziz K, Colby C, Escobedo M.  Textbook of Neonatal Resuscitation.  6th ed. Elk Grove Village (IL): American Academy of Pediatrics; 2011.

[4] Arabi AM, Ibrahim SA, Ahmed SE, MacGinnea F, Hawkes G, Dempsey E, et al. Skills retention in Sudanese village midwives 1 year following Helping Babies Breathe training. Arch Dis Child. 2016;101:439-42. <https://doi.org/10.1136/archdischild-2015-309190>.

[5] Arlington L, Kairuki AK, Isangula KG, Meda RA, Thomas E, Temu A, et al. Implementation of "Helping Babies Breathe": A 3-Year Experience in Tanzania. Pediatrics. 2017;139. <https://doi.org/10.1542/peds.2016-2132>.

[6] Athanasopoulou SG, Cicero M, Sanseau E, Kou M, Auerbach M. Use of a SimBox, a Video-Augmented, Newborn Resuscitation Simulation for Prehospital Providers to Measure Clinical Performance and Educational Experience. Cureus. 2024;16:e57925. <https://doi.org/10.7759/cureus.57925>.

[7] American Academy of Pediatrics; Laerdal Global Health. Helping Babies Breathe: Implementation guide. Strengthening neonatal resuscitation in sustainable programs of essential newborn care. Elk Grove Village (IL): American Academy of Pediatrics; 2011.

[8] Goudar SS, Somannavar MS, Clark R, Lockyer JM, Revankar AP, Fidler HM, et al. Stillbirth and newborn mortality in India after helping babies breathe training. Pediatrics. 2013;131:e344-52. <https://doi.org/10.1542/peds.2012-2112>.

[9] Joho AA, Kibusi SM, Mwampagatwa I. Predictors of Helping Babies Breathe knowledge and skills among nurses in primary health settings in Dodoma region, Tanzania. BMC Pregnancy Childbirth. 2020;20:150. <https://doi.org/10.1186/s12884-020-2782-9>.

[10] Kc A, Wrammert J, Nelin V, Clark RB, Ewald U, Peterson S, et al. Evaluation of Helping Babies Breathe Quality Improvement Cycle (HBB-QIC) on retention of neonatal resuscitation skills six months after training in Nepal. BMC Pediatr. 2017;17:103. <https://doi.org/10.1186/s12887-017-0853-5>.

[11] Musafili A, Essén B, Baribwira C, Rukundo A, Persson L. Evaluating Helping Babies Breathe: training for healthcare workers at hospitals in Rwanda. Acta Paediatr. 2013;102:e34-8. <https://doi.org/10.1111/apa.12034>.

[12] Seto TL, Tabangin ME, Josyula S, Taylor KK, Vasquez JC, Kamath-Rayne BD. Educational outcomes of Helping Babies Breathe training at a community hospital in Honduras. Perspect Med Educ. 2015;4:225-32. <https://doi.org/10.1007/s40037-015-0214-8>.

[13] Singhal N, Lockyer J, Fidler H, Keenan W, Little G, Bucher S, et al. Helping Babies Breathe: global neonatal resuscitation program development and formative educational evaluation. Resuscitation. 2012;83:90-6. <https://doi.org/10.1016/j.resuscitation.2011.07.010>.

[14] Tabangin ME, Josyula S, Taylor KK, Vasquez JC, Kamath-Rayne BD. Resuscitation skills after Helping Babies Breathe training: a comparison of varying practice frequency and impact on retention of skills in different types of providers. Int Health. 2018;10:163-71. <https://doi.org/10.1093/inthealth/ihy017>.

[15] Umunyana J, Sayinzoga F, Ricca J, Favero R, Manariyo M, Kayinamura A, et al. A practice improvement package at scale to improve management of birth asphyxia in Rwanda: a before-after mixed methods evaluation. BMC Pregnancy Childbirth. 2020;20:583. <https://doi.org/10.1186/s12884-020-03181-7>.

[16] Wilson GM, Ame AM, Khatib MM, Rende EK, Hartman AM, Blood-Siegfried J. Helping Babies Breathe implementation in Zanzibar, Tanzania. Int J Nurs Pract. 2017;23. <https://doi.org/10.1111/ijn.12561>.

[17] Wilson GM, Ame AM, Khatib MM, Khalfan BS, Thompson J, Blood-Siegfried J. Helping Babies Breathe (2nd edition) implementation on a shoestring budget in Zanzibar, Tanzania. Matern Health Neonatol Perinatol. 2020;6:3. <https://doi.org/10.1186/s40748-020-00117-z>.

[18] Mistry SC, Lin R, Mumphansha H, Kettley LC, Pearson JA, Akrimi S, et al. Newborn Resuscitation Skills in Health Care Providers at a Zambian Tertiary Center, and Comparison to World Health Organization Standards. Anesth Analg. 2018;127:217-23. <https://doi.org/10.1213/ane.0000000000003337>.

[19] Seto TL, Tabangin ME, Taylor KK, Josyula S, Vasquez JC, Kamath-Rayne BD. Breaking Down the Objective Structured Clinical Examination: An Evaluation of the Helping Babies Breathe OSCEs. Simul Healthc. 2017;12:226-32. <https://doi.org/10.1097/sih.0000000000000224>.

[20] Bameri F, Ghaderi R, Aboubakri O, Heydarikhayat N. Effect of continuous workshop training of the helping babies breathe program on the retention of midwives' knowledge and skills: A clinical trial study. Nurse Educ Pract. 2024;78:104020. <https://doi.org/10.1016/j.nepr.2024.104020>.

[21] Chan NH, Merali HS, Mistry N, Kealey R, Campbell DM, Morris SK, et al. Utilization of a novel mobile application, "HBB Prompt", to reduce Helping Babies Breathe skills decay. PLOS Glob Public Health. 2023;3:e0000705. <https://doi.org/10.1371/journal.pgph.0000705>.

[22] Chaulagain DR, K CA, Wrammert J, Brunell O, Basnet O, Malqvist M. Effect of a scaled-up quality improvement intervention on health workers' competence on neonatal resuscitation in simulated settings in public hospitals: A pre-post study in Nepal. PLoS One. 2021;16:e0250762. <https://doi.org/10.1371/journal.pone.0250762>.

[23] Cordova E, Al-Rousan T, Castillo-Angeles M, Aftab S, Nelson BD. Effect of low-cost interventions on the retention of knowledge and skills following Helping Babies Breathe training. Int J Gynaecol Obstet. 2018;142:248-54. <https://doi.org/10.1002/ijgo.12512>.

[24] Data S, Mirette D, Cherop M, Bajunirwe F, Kyakwera C, Robinson T, et al. Peer Learning and Mentorship for Neonatal Management Skills: A Cluster-Randomized Trial. Pediatrics. 2022;150. <https://doi.org/10.1542/peds.2021-054471>.

[25] Draiko CV, Yamarat K, Panza A, Draleru J. Knowledge, skills and competency retention among health workers one year after completing helping babies breathe training in South Sudan. Pan Afr Med J. 2019;33:175. <https://doi.org/10.11604/pamj.2019.33.175.17560>.

[26] Vunni Draiko C, Yamarat K, Panza A, Draleru J. Evaluation of retention of knowledge, skill and competency of health workers one year after completion of the Helping Babies Breathe training program in South Sudan [version 2; peer review: 2 approved with reservations]. F1000Research. 2019;8. <https://doi.org/10.12688/f1000research.17560.2>.

[27] Drake M, Bishanga DR, Temu A, Njozi M, Thomas E, Mponzi V, et al. Structured on-the-job training to improve retention of newborn resuscitation skills: a national cohort Helping Babies Breathe study in Tanzania. BMC Pediatr. 2019;19:51. <https://doi.org/10.1186/s12887-019-1419-5>.

[28] Eblovi D, Kelly P, Afua G, Agyapong S, Dante S, Pellerite M. Retention and use of newborn resuscitation skills following a series of helping babies breathe trainings for midwives in rural Ghana. Glob Health Action. 2017;10:1387985. <https://doi.org/10.1080/16549716.2017.1387985>.

[29] Ezenwa BN, Umoren R, Fajolu IB, Hippe DS, Bucher S, Purkayastha S, et al. Using Mobile Virtual Reality Simulation to Prepare for In-Person Helping Babies Breathe Training: Secondary Analysis of a Randomized Controlled Trial (the eHBB/mHBS Trial). JMIR Med Educ. 2022;8:e37297. <https://doi.org/10.2196/37297>.

[30] Heard Stittum AJ, Edwards EM, Abayneh M, Gebremedhin AD, Horn D, Berkelhamer SK, et al. Impact of an Educational Clinical Video Combined with Standard Helping Babies Breathe Training on Acquisition and Retention of Knowledge and Skills among Ethiopian Midwives. Children (Basel). 2023;10. <https://doi.org/10.3390/children10111782>.

[31] Hosseini SR, Naghdi R, Atarodi-Kashani Z, Sharifzadeh G, Bameri F. Impact of Continued Social Network-based Learning Based on Midwives Knowledge and Implementation of the Helping Babies Breathe Program. Iran J Nurs Midwifery Res. 2023;28:509-13. <https://doi.org/10.4103/ijnmr.ijnmr_46_22>.

[32] Kalabamu FS, Daudi V, Moshiro R, Kamala B, Mdoe P, Bishanga D, et al. Neonatal resuscitation skills acquisition among healthcare providers after Helping Babies Breathe simulation training using improved tools across two regions in Tanzania. Adv Simul (Lond). 2025;10:6. <https://doi.org/10.1186/s41077-025-00338-2>.

[33] Kfoury P, Maalouf F, Nasser F, Gulgulian T, Charafeddine L. In-Person Versus Online Training in Simulations of Helping Babies Breathe: A Randomized Controlled Trial. Cureus. 2024;16:e64677. <https://doi.org/10.7759/cureus.64677>.

[34] Jones-Bamman C, Niermeyer S, McConnell K, Thomas JF, Olson C. Teaching Helping Babies Breathe via Telehealth: A New Application in Rural Guatemala. Biomed Hub. 2019;4:1-6. <https://doi.org/10.1159/000502934>.

[35] Mediratta RP, Clary MK, Liang JW, Daniels K, Muhe LM, Lee HC, et al. Remote versus in-person pre-service neonatal resuscitation training: A noninferiority randomized controlled trial in Ethiopia. Resuscitation. 2025;209:110556. <https://doi.org/10.1016/j.resuscitation.2025.110556>.

[36] Mubeen K, Baig M, Abbas S, Adnan F, Lakhani A, Bhamani SS, et al. Helping babies breathe: assessing the effectiveness of simulation-based high-frequency recurring training in a community-based setting of Pakistan. BMC Pediatr. 2021;21:555. <https://doi.org/10.1186/s12887-021-03014-2>.

[37] Munyaw Y, Gidabayda J, Yeconia A, Guga G, Mduma E, Mdoe P. Beyond research: improved perinatal care through scale-up of a Moyo fetal heart rate monitor coupled with simulation training in northern Tanzania for helping babies breathe. BMC Pediatr. 2022;22:191. <https://doi.org/10.1186/s12887-022-03249-7>.

[38] Sendo EG, Aga F, Gebrewold LA. Retention of knowledge and skills among midwives one year after completing helping babies breathe pre-service training in Ethiopia: a non-randomized quasi-experimental study. Matern Health Neonatol Perinatol. 2025;11:15. <https://doi.org/10.1186/s40748-025-00201-2>.

[39] Sihota D, Lee Him R, Dominguez G, Harrison L, Vaivada T, Bhutta ZA. Effectiveness of Neonatal Resuscitation Training Programs, Implementation, and Scale-Up in Low- and Middle-Income Countries. Neonatology. 2025;122:52-83. <https://doi.org/10.1159/000542539>.

[40] Umoren R, Bucher S, Hippe DS, Ezenwa BN, Fajolu IB, Okwako FM, et al. eHBB: a randomised controlled trial of virtual reality or video for neonatal resuscitation refresher training in healthcare workers in resource-scarce settings. BMJ Open. 2021;11:e048506. <https://doi.org/10.1136/bmjopen-2020-048506>.

[41] Zujkowski M, Ehrlich S, Liu C, Sun Q, Zackoff M, Brady JM, et al. A Needs Assessment of Labor and Delivery Nurses Performing NRP in the Delivery Room. Matern Child Health J. 2025;29:23-30. <https://doi.org/10.1007/s10995-024-04030-1>.

[42] Hatamleh R, Abujilban S, Abuhammad S, Ariag DMA, Joseph RA. Effect of the Helping Babies Breathe Training Program: An Experimental Study on Jordanian Midwives' Knowledge and Skills. J Perinat Neonatal Nurs. 2021;35:E11-e7. <https://doi.org/10.1097/jpn.0000000000000485>.

[43] Odongkara B, Tylleskär T, Pejovic N, Achora V, Mukunya D, Ndeezi G, et al. Adding video-debriefing to Helping-Babies-Breathe training enhanced retention of neonatal resuscitation knowledge and skills among health workers in Uganda: a cluster randomized trial. Glob Health Action. 2020;13:1743496. <https://doi.org/10.1080/16549716.2020.1743496>.

[44] Barbato AL, Wetzel EA, Li W, Bo N, Mayer L, Byrne BJ. Simulation Education for Preterm Infant Delivery Room Management at Community Hospitals. Pediatrics. 2020;146. <https://doi.org/10.1542/peds.2019-3688>.

[45] Barry JS, Gibbs MD, Rosenberg AA. A delivery room-focused education and deliberate practice can improve pediatric resident resuscitation training. J Perinatol. 2012;32:920-6. <https://doi.org/10.1038/jp.2012.27>.

[46] Bender J, Kennally K, Shields R, Overly F. Does simulation booster impact retention of resuscitation procedural skills and teamwork? J Perinatol. 2014;34:664-8. <https://doi.org/10.1038/jp.2014.72>.

[47] Bibl K, Eibensteiner F, Ritschl V, Steinbauer P, Berger A, Olischar M, et al. NeoCheck: A New Checklist to Assess Performance during Newborn Life Support-A Validation Study. Children (Basel). 2023;10. <https://doi.org/10.3390/children10061013>.

[48] Billner-Garcia RM, Spilker A. Development and Implementation of a Game-Based Neonatal Resuscitation Refresher Training: Effect on Registered Nurse Knowledge, Skills, Motivation, Engagement. J Nurses Prof Dev. 2024;40:24-8. <https://doi.org/10.1097/nnd.0000000000000953>.

[49] Binkhorst M, van de Wiel I, Draaisma JMT, van Heijst AFJ, Antonius T, Hogeveen M. Neonatal resuscitation guideline adherence: simulation study and framework for improvement. Eur J Pediatr. 2020;179:1813-22. <https://doi.org/10.1007/s00431-020-03693-6>.

[50] Brathwaite KP, Bryce F, Moyer LB, Engmann C, Twum-Danso NAY, Kamath-Rayne BD, et al. Evaluation of two newborn resuscitation training strategies in regional hospitals in Ghana. Resusc Plus. 2020;1-2:100001. <https://doi.org/10.1016/j.resplu.2020.100001>.

[51] Brogaard L, Hvidman L, Esberg G, Finer N, Hjorth-Hansen KR, Manser T, et al. Teamwork and Adherence to Guideline on Newborn Resuscitation-Video Review of Neonatal Interdisciplinary Teams. Front Pediatr. 2022;10:828297. <https://doi.org/10.3389/fped.2022.828297>.

[52] Caldelari M, Floris L, Marchand C, Schuler Barazzoni M. Maintaining the knowledge and neonatal resuscitation skills of student midwives 6 months after an educational program. Arch Pediatr. 2019;26:385-92. <https://doi.org/10.1016/j.arcped.2019.05.001>.

[53] Campbell DM, Barozzino T, Farrugia M, Sgro M. High-fidelity simulation in neonatal resuscitation. Paediatr Child Health. 2009;14:19-23. <https://doi.org/10.1093/pch/14.1.19>.

[54] Carbine DN, Finer NN, Knodel E, Rich W. Video recording as a means of evaluating neonatal resuscitation performance. Pediatrics. 2000;106:654-8. <https://doi.org/10.1542/peds.106.4.654>.

[55] Cavicchiolo ME, Cavallin F, Bertuola F, Pizzol D, Segafredo G, Wingi OM, et al. Effect of a Low-Dose/High-Frequency Training on Real-Life Neonatal Resuscitation in a Low-Resource Setting. Neonatology. 2018;114:294-302. <https://doi.org/10.1159/000490370>.

[56] Carlo WA, Wright LL, Chomba E, McClure EM, Carlo ME, Bann CM, et al. Educational impact of the neonatal resuscitation program in low-risk delivery centers in a developing country. J Pediatr. 2009;154:504-8.e5. <https://doi.org/10.1016/j.jpeds.2008.10.005>.

[57] Cavallin F, Calgaro S, Borellini M, Magnani M, Beltramini G, Seni AHA, et al. Midwives' Evaluation of a Neonatal Resuscitation in High- and Low-Resource Settings. Front Pediatr. 2021;9:644308. <https://doi.org/10.3389/fped.2021.644308>.

[58] Chan NH, Mistry N, Campbell DM. A Simulation-Based Pilot Study of a Mobile Application (NRP Prompt) as a Cognitive Aid for Neonatal Resuscitation Training. Simul Healthc. 2019;14:146-56. <https://doi.org/10.1097/sih.0000000000000353>.

[59] Benguigui L, Le Gouzouguec S, Balanca B, Ristovski M, Putet G, Butin M, et al. A Customizable Digital Cognitive Aid for Neonatal Resuscitation: A Simulation-Based Randomized Controlled Trial. Simul Healthc. 2024;19:302-8. <https://doi.org/10.1097/sih.0000000000000790>.

[60] Dhungana R, Chalise M, Clark RB. An assessment of immediate newborn care readiness and availability in Nepal. Glob Health Action. 2023;16:2289735. <https://doi.org/10.1080/16549716.2023.2289735>.

[61] Cordero L, Hart BJ, Hardin R, Mahan JD, Nankervis CA. Deliberate practice improves pediatric residents' skills and team behaviors during simulated neonatal resuscitation. Clin Pediatr (Phila). 2013;52:747-52. <https://doi.org/10.1177/0009922813488646>.

[62] Cordero L, Hart BJ, Hardin R, Mahan JD, Giannone PJ, Nankervis CA. Pediatrics residents' preparedness for neonatal resuscitation assessed using high-fidelity simulation. J Grad Med Educ. 2013;5:399-404. <https://doi.org/10.4300/jgme-d-12-00192.1>.

[63] Cronin C, Cheang S, Hlynka D, Adair E, Roberts S. Videoconferencing can be used to assess neonatal resuscitation skills. Med Educ. 2001;35:1013-23. <https://doi.org/10.1046/j.1365-2923.2001.01055.x>.

[64] Curran V, Fleet L, White S, Bessell C, Deshpandey A, Drover A, et al. A randomized controlled study of manikin simulator fidelity on neonatal resuscitation program learning outcomes. Adv Health Sci Educ Theory Pract. 2015;20:205-18. <https://doi.org/10.1007/s10459-014-9522-8>.

[65] Cusack J, Fawke J. Neonatal resuscitation: are your trainees performing as you think they are? A retrospective review of a structured resuscitation assessment for neonatal medical trainees over an 8-year period. Arch Dis Child Fetal Neonatal Ed. 2012;97:F246-8. <https://doi.org/10.1136/archdischild-2011-300548>.

[66] Das MK, Chaudhary C, Bisht SS, Maria A, Jain A, Kaushal SK, et al. Retention of Knowledge and Skill of Birth Attendants in Newborn Care and Resuscitation after 1 Year in Clinical Practice: An Experience from India. Journal of Clinical Neonatology. 2018;7:89-95. <https://doi.org/10.4103/jcn.JCN_9_18>.

[67] De Bernardo G, Sordino D, Cavallin F, Mardegan V, Doglioni N, Tataranno ML, et al. Performances of low level hospital health caregivers after a neonatal resuscitation course. Ital J Pediatr. 2016;42:100. <https://doi.org/10.1186/s13052-016-0313-0>.

[68] Delaney MM, Usmanova G, Nair TS, Neergheen VL, Miller K, Fishman E, et al. Does Quality Certification Work? An Assessment of Manyata, a Childbirth Quality Program in India's Private Sector. Glob Health Sci Pract. 2022;10. <https://doi.org/10.9745/ghsp-d-22-00093>.

[69] Ding X, Wang L, Msellem MI, Hu Y, Qiu J, Liu S, et al. Evaluation of a Neonatal Resuscitation Training Programme for Healthcare Professionals in Zanzibar, Tanzania: A Pre-post Intervention Study. Front Pediatr. 2021;9:693583. <https://doi.org/10.3389/fped.2021.693583>.

[70] Farhadi R, Azandehi BK, Amuei F, Ahmadi M, Zazoly AZ, Ghorbani AA. Enhancing residents' neonatal resuscitation competency through team-based simulation training: an intervention educational study. BMC Med Educ. 2023;23:743. <https://doi.org/10.1186/s12909-023-04704-4>.

[71] Ford BS, Hagadorn JI, Trzaski JM. Examining the Validity of Pediatric Resident Self-Assessment in the Delivery Room. Am J Perinatol. 2023;40:624-9. <https://doi.org/10.1055/s-0041-1730361>.

[72] Gamboa OA, Agudelo SI, Maldonado MJ, Leguizamón DC, Cala SM. Evaluation of two strategies for debriefing simulation in the development of skills for neonatal resuscitation: a randomized clinical trial. BMC Res Notes. 2018;11:739. <https://doi.org/10.1186/s13104-018-3831-6>.

[73] Gelbart B, Hiscock R, Barfield C. Assessment of neonatal resuscitation performance using video recording in a perinatal centre. J Paediatr Child Health. 2010;46:378-83. <https://doi.org/10.1111/j.1440-1754.2010.01747.x>.

[74] Ghoman SK, Cutumisu M, Schmölzer GM. Using the RETAIN Tabletop Simulator as a Summative Assessment Tool for Neonatal Resuscitation Healthcare Professionals: A Pilot Study. Front Pediatr. 2020;8:569776. <https://doi.org/10.3389/fped.2020.569776>.

[75] Greer JA, Lutgendorf MA, Ennen CS, Van Petten L, Modzik A, Salas D, et al. Obstetric Simulation Training and Teamwork: Immediate Impact on Knowledge, Teamwork, and Adherence to Hemorrhage Protocols. Simul Healthc. 2023;18:32-41. <https://doi.org/10.1097/sih.0000000000000641>.

[76] Gross IT, Whitfill T, Redmond B, Couturier K, Bhatnagar A, Joseph M, et al. Comparison of Two Telemedicine Delivery Modes for Neonatal Resuscitation Support: A Simulation-Based Randomized Trial. Neonatology. 2020;117:159-66. <https://doi.org/10.1159/000504853>.

[77] Holm-Hansen CC, Poulsen A, Skytte TB, Stensgaard CN, Bech CM, Lopes MN, et al. Video recording as an objective assessment tool of health worker performance in neonatal resuscitation at a district hospital in Pemba, Tanzania: a feasibility study. BMJ Open. 2022;12:e060642. <https://doi.org/10.1136/bmjopen-2021-060642>.

[78] Holm-Hansen CC, Lund S, Skytte TB, Molenaar J, Steensgaard CN, Mohd UA, et al. Neonatal mortality and video assessment of resuscitation in four district hospitals in Pemba, Tanzania. Pediatr Res. 2024;95:712-21. <https://doi.org/10.1038/s41390-023-02824-7>.

[79] Horiuchi S, Rattana S, Saysanasongkham B, Kounnavongsa O, Kubota S, Inoue M, et al. Effectiveness of self-managed continuous monitoring for maintaining high-quality early essential newborn care compared to supervision visit in Lao PDR: a cluster randomised controlled trial. BMC Health Serv Res. 2021;21:460. <https://doi.org/10.1186/s12913-021-06481-6>.

[80] Hundscheid T, Bruinenberg J, Dudink J, de Jonge R, Hogeveen M. Performing newborn life support in advance of neonatal advanced life support course-back to basics? Eur J Pediatr. 2021;180:1647-51. <https://doi.org/10.1007/s00431-020-03917-9>.

[81] Johnson C, Shen E, Winn K, Digiacobbe G, Akinola M. Neonatal Resuscitation: A Blended Learning Curriculum for Medical and Physician Assistant Students. MedEdPORTAL. 2020;16:10921. <https://doi.org/10.15766/mep_2374-8265.10921>.

[82] Kamath-Rayne BD, Tabangin ME, Taylor RG, Geis GL. Retention of Basic Neonatal Resuscitation Skills and Bag-Mask Ventilation in Pediatric Residents Using Just-in-Place Simulation of Varying Frequency and Intensity: A Pilot Randomized Controlled Study. Hosp Pediatr. 2019;9:681-9. <https://doi.org/10.1542/hpeds.2018-0219>.

[83] Kamau PT, Koech M, Hecht SM, McHenry MS, Songok J. Assessment of neonatal resuscitation skills among healthcare workers in Uasin Gishu County, Kenya. SAGE Open Med. 2022;10:20503121221119296. <https://doi.org/10.1177/20503121221119296>.

[84] Kannan Loganathan P, Garg A, McNicol R, Wall C, Pointon M, McMeekin P, et al. Assessment of Visual Attention in Teams with or without Dedicated Team Leaders: A Neonatal Simulation-Based Pilot Randomised Cross-Over Trial Utilising Low-Cost Eye-Tracking Technology. Children (Basel). 2024;11. <https://doi.org/10.3390/children11081023>.

[85] Kane SK, Lorant DE. Creation and Validation of Tool to Assess Resident Competence in Neonatal Resuscitation. Acad Pediatr. 2019;19:394-8. <https://doi.org/10.1016/j.acap.2018.09.004>.

[86] Khriesat W, Kassab M, Hamadneh S, Mohammad K, Hamadneh J, Khader YS. Infant Resuscitation Practices of Midwives in a Developing Country. Adv Neonatal Care. 2017;17:400-6. <https://doi.org/10.1097/anc.0000000000000418>.

[87] Kim YM, Ansari N, Kols A, Tappis H, Currie S, Zainullah P, et al. Assessing the capacity for newborn resuscitation and factors associated with providers' knowledge and skills: a cross-sectional study in Afghanistan. BMC Pediatr. 2013;13:140. <https://doi.org/10.1186/1471-2431-13-140>.

[88] Lizotte MH, Janvier A, Latraverse V, Lachance C, Walker CD, Barrington KJ, et al. The Impact of Neonatal Simulations on Trainees' Stress and Performance: A Parallel-Group Randomized Trial. Pediatr Crit Care Med. 2017;18:434-41. <https://doi.org/10.1097/pcc.0000000000001119>.

[89] Lockyer J, Singhal N, Fidler H, Weiner G, Aziz K, Curran V. The development and testing of a performance checklist to assess neonatal resuscitation megacode skill. Pediatrics. 2006;118:e1739-44. <https://doi.org/10.1542/peds.2006-0537>.

[90] Bang A, Patel A, Bellad R, Gisore P, Goudar SS, Esamai F, et al. Helping Babies Breathe (HBB) training: What happens to knowledge and skills over time? BMC Pregnancy Childbirth. 2016;16:364. <https://doi.org/10.1186/s12884-016-1141-3>.

[91] Charafeddine L, Badran M, Nakad P, Ammar W, Yunis K. Strategic assessment of implementation of neonatal resuscitation training at a national level. Pediatr Int. 2016;58:595-600. <https://doi.org/10.1111/ped.12868>.

[92] Lizotte MH, Latraverse V, Moussa A, Lachance C, Barrington K, Janvier A. Trainee Perspectives on Manikin Death During Mock Codes. Pediatrics. 2015;136:e93-8. <https://doi.org/10.1542/peds.2014-3910>.

[93] Nimbalkar A, Patel D, Kungwani A, Phatak A, Vasa R, Nimbalkar S. Randomized control trial of high fidelity vs low fidelity simulation for training undergraduate students in neonatal resuscitation. BMC Res Notes. 2015;8:636. <https://doi.org/10.1186/s13104-015-1623-9>.

[94] Patel J, Posencheg M, Ades A. Proficiency and retention of neonatal resuscitation skills by pediatric residents. Pediatrics. 2012;130:515-21. <https://doi.org/10.1542/peds.2012-0149>.

[95] Weiner GM, Menghini K, Zaichkin J, Caid AE, Jacoby CJ, Simon WM. Self-directed versus traditional classroom training for neonatal resuscitation. Pediatrics. 2011;127:713-9. <https://doi.org/10.1542/peds.2010-2829>.

[96] Bould MD, Hayter MA, Campbell DM, Chandra DB, Joo HS, Naik VN. Cognitive aid for neonatal resuscitation: a prospective single-blinded randomized controlled trial. Br J Anaesth. 2009;103:570-5. <https://doi.org/10.1093/bja/aep221>.

[97] Katakam LI, Trickey AW, Thomas EJ. Speaking up and sharing information improves trainee neonatal resuscitations. J Patient Saf. 2012;8:202-9. <https://doi.org/10.1097/PTS.0b013e3182699b4f>.

[98] Magee MJ, Farkouh-Karoleski C, Rosen TS. Improvement of Immediate Performance in Neonatal Resuscitation Through Rapid Cycle Deliberate Practice Training. J Grad Med Educ. 2018;10:192-7. <https://doi.org/10.4300/jgme-d-17-00467.1>.

[99] Maya-Enero S, Botet-Mussons F, Figueras-Aloy J, Izquierdo-Renau M, Thió M, Iriondo-Sanz M. Adherence to the neonatal resuscitation algorithm for preterm infants in a tertiary hospital in Spain. BMC Pediatr. 2018;18:319. <https://doi.org/10.1186/s12887-018-1288-3>.

[100] McCaw JM, Yelton SEG, Tackett SA, Rapal R, Gamalinda AN, Arellano-Reyles A, et al. Effect of repeat refresher courses on neonatal resuscitation skill decay: an experimental comparative study of in-person and video-based simulation training. Adv Simul (Lond). 2023;8:7. <https://doi.org/10.1186/s41077-023-00244-5>.

[101] Meggiolaro L, Weiner G, Staffler A, Lupi F, Canesso T, Trevisanuto D. NeoScore: a new tool to assess technical and non-technical skills during neonatal resuscitation. J Perinatol. 2025. <https://doi.org/10.1038/s41372-025-02210-7>.

[102] Mersha A, Shibiru S, Gultie T, Degefa N, Bante A. Training and well-equipped facility increases the odds of skills of health professionals on helping babies breathe in public hospitals of Southern Ethiopia: cross-sectional study. BMC Health Serv Res. 2019;19:946. <https://doi.org/10.1186/s12913-019-4772-z>.

[103] Mileder LP, Bereiter M, Schwaberger B, Wegscheider T. Telesimulation for the Training of Medical Students in Neonatal Resuscitation. Children (Basel). 2023;10. <https://doi.org/10.3390/children10091502>.

[104] Nadler I, Sanderson PM, Van Dyken CR, Davis PG, Liley HG. Presenting video recordings of newborn resuscitations in debriefings for teamwork training. BMJ Qual Saf. 2011;20:163-9. <https://doi.org/10.1136/bmjqs.2010.043547>.

[105] Nickerson JE, Webb T, Boehm L, Neher H, Wong L, LaMonica J, et al. Difficult Delivery and Neonatal Resuscitation: A Novel Simulation for Emergency Medicine Residents. West J Emerg Med. 2019;21:102-7. <https://doi.org/10.5811/westjem.2019.10.43913>.

[106] Nvonako H, Ojee E, Masika M, Sandie A, Wamalwa D, Wasunna A. Effect of in-hospital training in newborn resuscitation on the competence of health-care workers in resuscitating newborn infants at birth at Mboppi Baptist Hospital, Douala, Cameroon. Pan Afr Med J. 2022;42:169. <https://doi.org/10.11604/pamj.2022.42.169.32816>.

[107] Paliatsiou S, Xanthos T, Wyllie J, Volaki P, Sokou R, Bikouli D, et al. Theoretical knowledge and skill retention 3 and 6 months after a European Newborn Life Support provider course. Am J Emerg Med. 2021;49:83-8. <https://doi.org/10.1016/j.ajem.2021.05.048>.

[108] Reisman J, Martineau N, Kairuki A, Mponzi V, Meda AR, Isangula KG, et al. Validation of a novel tool for assessing newborn resuscitation skills among birth attendants trained by the Helping Babies Breathe program. Int J Gynaecol Obstet. 2015;131:196-200. <https://doi.org/10.1016/j.ijgo.2015.05.019>.

[109] Chinbuah MA, Taylor M, Serpa M, Mazia G, Cofie PK, Kwarah W, et al. Scaling up Ghana's national newborn care initiative: integrating 'helping babies breathe' (HBB), 'essential care for every baby' (ECEB), and newborn 'infection prevention' (IP) trainings. BMC Health Serv Res. 2020;20:739. <https://doi.org/10.1186/s12913-020-05225-2>.

[110] Roitsch CM, Patricia KE, Hagan JL, Arnold JL, Sundgren NC. Tablet-Based Decision Support Tool Improves Performance of Neonatal Resuscitation: A Randomized Trial in Simulation. Simul Healthc. 2020;15:243-50. <https://doi.org/10.1097/sih.0000000000000422>.

[111] Neveln NK, Khattab M, Hagan JL, Fortunov RM, Sundgren NC. A recorder/time coach decreases time errors during neonatal resuscitation: A randomized, simulation-based clinical trial. Resusc Plus. 2023;15:100411. <https://doi.org/10.1016/j.resplu.2023.100411>.

[112] Rovamo L, Mattila MM, Andersson S, Rosenberg P. Assessment of newborn resuscitation skills of physicians with a simulator manikin. Arch Dis Child Fetal Neonatal Ed. 2011;96:F383-9. <https://doi.org/10.1136/adc.2010.194043>.

[113] Rovamo LM, Mattila MM, Andersson S, Rosenberg PH. Testing of midwife neonatal resuscitation skills with a simulator manikin in a low-risk delivery unit. Pediatr Int. 2013;55:465-71. <https://doi.org/10.1111/ped.12083>.

[114] Sarvan S, Efe E. The effect of neonatal resuscitation training based on a serious game simulation method on nursing students' knowledge, skills, satisfaction and self-confidence levels: A randomized controlled trial. Nurse Educ Today. 2022;111:105298. <https://doi.org/10.1016/j.nedt.2022.105298>.

[115] Liaqat M, Hussain M, Afzal M, Altaf M, Khan S, Gilani SA, et al. Efficacy of pedagogical framework in neonatal resuscitation skill learning in a resource-limited setting: a randomized controlled trial. BMC Med Educ. 2021;21:436. <https://doi.org/10.1186/s12909-021-02846-x>.

[116] Sawyer T, Sierocka-Castaneda A, Chan D, Berg B, Lustik M, Thompson M. Deliberate practice using simulation improves neonatal resuscitation performance. Simul Healthc. 2011;6:327-36. <https://doi.org/10.1097/SIH.0b013e31822b1307>.

[117] Hadfield BR, Sawyer T, Moreira AG, Farner R, Vasquez MM. Rapid cycle deliberate practice improves resident performance during ELBW resuscitation. J Neonatal Perinatal Med. 2024;17:31-40. <https://doi.org/10.3233/npm-230102>.

[118] Sawyer T, Sierocka-Castaneda A, Chan D, Berg B, Lustik M, Thompson M. The effectiveness of video-assisted debriefing versus oral debriefing alone at improving neonatal resuscitation performance: a randomized trial. Simul Healthc. 2012;7:213-21. <https://doi.org/10.1097/SIH.0b013e3182578eae>.

[119] Sawyer T, Leonard D, Sierocka-Castaneda A, Chan D, Thompson M. Correlations between technical skills and behavioral skills in simulated neonatal resuscitations. J Perinatol. 2014;34:781-6. <https://doi.org/10.1038/jp.2014.93>.

[120] Litke-Wager C, Delaney H, Mu T, Sawyer T. Impact of Task-Oriented Role Assignment on Neonatal Resuscitation Performance: A Simulation-Based Randomized Controlled Trial. Am J Perinatol. 2021;38:914-21. <https://doi.org/10.1055/s-0039-3402751>.

[121] Matterson HH, Szyld D, Green BR, Howell HB, Pusic MV, Mally PV, et al. Neonatal resuscitation experience curves: simulation based mastery learning booster sessions and skill decay patterns among pediatric residents. J Perinat Med. 2018;46:934-41. <https://doi.org/10.1515/jpm-2017-0330>.

[122] Shikuku DN, Milimo B, Ayebare E, Gisore P, Nalwadda G. Quality of Care during Neonatal Resuscitation in Kakamega County General Hospital, Kenya: A Direct Observation Study. Biomed Res Int. 2017;2017:2152487. <https://doi.org/10.1155/2017/2152487>.

[123] Sinha A, Nimbalkar SM, Pujara RK, Patel PR, Shinde MK, Sethi S, et al. SimCapture app video performance assessment versus real-time instructor-based performance evaluation of undergraduates in neonatal resuscitation-an agreement study. J Trop Pediatr. 2024;70. <https://doi.org/10.1093/tropej/fmae033>.

[124] Sinha A, Nimbalkar S, Shah D, Patel P, Patel J, Nagadia Q, et al. NeoNatalie Versus NeoNatalie Live Simulation for Training Undergraduate Students in Neonatal Resuscitation-A Randomized Control Trial. Int J Pediatr. 2025;2025:3159205. <https://doi.org/10.1155/ijpe/3159205>.

[125] Sintayehu Y, Desalew A, Geda B, Tiruye G, Mezmur H, Shiferaw K, et al. Basic neonatal resuscitation skills of midwives and nurses in Eastern Ethiopia are not well retained: An observational study. PLoS One. 2020;15:e0236194. <https://doi.org/10.1371/journal.pone.0236194>.

[126] Skåre C, Calisch TE, Saeter E, Rajka T, Boldingh AM, Nakstad B, et al. Implementation and effectiveness of a video-based debriefing programme for neonatal resuscitation. Acta Anaesthesiol Scand. 2018;62:394-403. <https://doi.org/10.1111/aas.13050>.

[127] Skåre C, Boldingh AM, Kramer-Johansen J, Calisch TE, Nakstad B, Nadkarni V, et al. Video performance-debriefings and ventilation-refreshers improve quality of neonatal resuscitation. Resuscitation. 2018;132:140-6. <https://doi.org/10.1016/j.resuscitation.2018.07.013>.

[128] Sloane AJ, Kenaley KM, Favara MT. Assessment of temporal variations in adherence to NRP using video recording in the delivery room. Resusc Plus. 2021;8:100162. <https://doi.org/10.1016/j.resplu.2021.100162>.

[129] Szyld EG, Aguilar A, Lloret SP, Pardo A, Fabres J, Castro A, et al. Self-directed video versus instructor-based neonatal resuscitation training: a randomized controlled blinded non-inferiority multicenter international study. J Perinatol. 2021;41:1583-9. <https://doi.org/10.1038/s41372-021-00941-x>.

[130] Tosif S, Jatobatu A, Maepioh A, Gray A, Sobel H, Mannava P, et al. Healthcare worker knowledge and skills following coaching in WHO early essential newborn care program in the Solomon Islands: a prospective multi-site cohort study. BMC Pregnancy Childbirth. 2020;20:84. <https://doi.org/10.1186/s12884-020-2739-z>.

[131] Trevisanuto D, Bertuola F, Lanzoni P, Cavallin F, Matediana E, Manzungu OW, et al. Effect of a Neonatal Resuscitation Course on Healthcare Providers' Performances Assessed by Video Recording in a Low-Resource Setting. PLoS One. 2015;10:e0144443. <https://doi.org/10.1371/journal.pone.0144443>.

[132] Tsang KD, Ottow MK, van Heijst AFJ, Antonius TAJ. Electronic Decision Support in the Delivery Room Using Augmented Reality to Improve Newborn Life Support Guideline Adherence: A Randomized Controlled Pilot Study. Simul Healthc. 2022;17:293-8. <https://doi.org/10.1097/sih.0000000000000631>.

[133] Vail B, Spindler H, Morgan MC, Cohen SR, Christmas A, Sah P, et al. Care of the mother-infant dyad: a novel approach to conducting and evaluating neonatal resuscitation simulation training in Bihar, India. BMC Pregnancy Childbirth. 2017;17:252. <https://doi.org/10.1186/s12884-017-1434-1>.

[134] Vail B, Morgan MC, Spindler H, Christmas A, Cohen SR, Walker DM. The power of practice: simulation training improving the quality of neonatal resuscitation skills in Bihar, India. BMC Pediatr. 2018;18:291. <https://doi.org/10.1186/s12887-018-1254-0>.

[135] van der Heide PA, van Toledo-Eppinga L, van der Heide M, van der Lee JH. Assessment of neonatal resuscitation skills: a reliable and valid scoring system. Resuscitation. 2006;71:212-21. <https://doi.org/10.1016/j.resuscitation.2006.04.009>.

[136] Finan E, Bismilla Z, Whyte HE, Leblanc V, McNamara PJ. High-fidelity simulator technology may not be superior to traditional low-fidelity equipment for neonatal resuscitation training. J Perinatol. 2012;32:287-92. <https://doi.org/10.1038/jp.2011.96>.

[137] Louvel AC, Dopff C, Loron G, Michelet D. Impact of a 3-Month Recall Using High-Fidelity Simulation or Screen-Based Simulation on Learning Retention During Neonatal Resuscitation Training for Residents in Anesthesia and Intensive Care: Randomized Controlled Trial. JMIR Serious Games. 2025;13:e57057. <https://doi.org/10.2196/57057>.

[138] Michelet D, Barre J, Truchot J, Piot MA, Cabon P, Tesniere A. Effect of Computer Debriefing on Acquisition and Retention of Learning After Screen-Based Simulation of Neonatal Resuscitation: Randomized Controlled Trial. JMIR Serious Games. 2020;8:e18633. <https://doi.org/10.2196/18633>.

[139] Lee MO, Brown LL, Bender J, Machan JT, Overly FL. A medical simulation-based educational intervention for emergency medicine residents in neonatal resuscitation. Acad Emerg Med. 2012;19:577-85. <https://doi.org/10.1111/j.1553-2712.2012.01361.x>.

[140] Woods J, Gagliardi L, Nara S, Phally S, Varang O, Viphou N, et al. An innovative approach to in-service training of maternal health staff in Cambodian hospitals. Int J Gynaecol Obstet. 2015;129:178-83. <https://doi.org/10.1016/j.ijgo.2014.10.034>.

[141] Xu C, Zhang Q, Xue Y, Yang Y, Chen Y, Yan W, et al. Neonatal resuscitation workshop for trainees in standardized medical residency training-a pilot practice in Shenzhen, China. Front Pediatr. 2023;11:1237747. <https://doi.org/10.3389/fped.2023.1237747>.

[142] Yamada NK, Fuerch JH, Halamek LP. Impact of Standardized Communication Techniques on Errors during Simulated Neonatal Resuscitation. Am J Perinatol. 2016;33:385-92. <https://doi.org/10.1055/s-0035-1565997>.

[143] Yamada NK, Yaeger KA, Halamek LP. Analysis and classification of errors made by teams during neonatal resuscitation. Resuscitation. 2015;96:109-13. <https://doi.org/10.1016/j.resuscitation.2015.07.048>.

[144] Yaylaci S, Guven F. The Effectiveness of Different E-Learning Modalities in Enhancing Neonatal Cardiopulmonary Resuscitation: Principles, Knowledge, and Communication Skills of Undergraduate Paramedic Students. Prehosp Disaster Med. 2021;36:576-85. <https://doi.org/10.1017/s1049023x21000832>.

[145] Yeo CL, Ho SKY, Tagamolila VC, Arunachalam S, Bharadwaj SS, Poon WB, et al. Use of web-based game in neonatal resuscitation - is it effective? BMC Med Educ. 2020;20:170. <https://doi.org/10.1186/s12909-020-02078-5>.

[146] Yoosoof F, Liyanage I, de Silva R, Samaraweera S. Videos of demonstration versus text and image-based material for pre-skill conceptualisation in flipped newborn resuscitation training for medical students: a pilot study. BMC Med Educ. 2022;22:839. <https://doi.org/10.1186/s12909-022-03926-2>.

**Table 1: Study and assessment tool characteristics**

| **Basic information** | |  |  |  |  |  |  |  |  |  |  |  |  |  |  |  |  |  |  |
| --- | --- | --- | --- | --- | --- | --- | --- | --- | --- | --- | --- | --- | --- | --- | --- | --- | --- | --- | --- |
| **First author** | **Year of publication** | **Country** | **Referenced by** | **Extra validity evidence gained by** | **Algorithm** | **Which update of the algorithm** | **Mode of tool development** | **Number of items included in the tool** | **Number of scoring posibilities** | **Weighted scoring** | **Setting** | **Fidelity of the manikin** | **Goal of the assessment** | **Number of pages of the tool** | **Scoring instructions available** | **Part of a validation study** | **Assessment scenario available** | **Type of assessors** | **Subheadings included** |
|  | | |  | | **1 = NLS/ERC 2 = NRP/AHA 3 = HBB 4 = Other 5 = Not reported** | **1 = ERC 2021 2 = ERC 2015 3 = ERC 2010  4 = NRP 2021 5 = NRP 2015  6 = NRP 2010**  **7 = NRP 2000 8 = NRP 2006 9 = HBB 2nd ed.  10 = HBB 1st ed.  11 = Other 12 = Not reported** | **1 = Original 2 = Modified version of existing tool 3 = Other 4 = Not reported** |  | **1 = Dichotomous 2 = Trichotomous 3 = 4 scoring options  4 = 3-point Likert 5 = 5-point Likert 6 = 7-point Likert 7 = Variable  8 = Other 9 = Not reported** | **1 = Yes 2 = No 3 = Not reported** | **1 = Video  2 = Direct visual observation 3 = Other 4 = Not reported / not applicable** | **1 = High 2 = Medium 3 = Low 4 = Not reported / not applicable 5 = Real life** | **1 = Formative 2 = Summative 3 = Both 4= Other 5 = Not reported / not applicable** | **1 = 1 page 2 = more than 1 page** | **1 = Extensive 2 = A few 3 = No** | **1 = Yes 2 = No** | **1 = Yes 2 = No** | **1 = NLS/NRP/HBB- instructors 2 = Physicians  3 = Residents  4 = Physician assistants / nurse practitioners  5 = Nurses  6 = Paramedics 7 = Medical students 8 = Midwives 9 = Other 10 = Not reported** | **1 = Yes 2 = No** |
| **HBB 1st edition** | 2010 | N.A. | Goudar et al. 2012, Joho et al. 2020, Kc et al. 2017, Musafili et al. 2012, Seto et al. 2014, Singhal et al. 2012, Tabangin et al. 2018, Umunyaya et al. 2020, Wilson et al. 2017, Wilson et al. 2020 | Arabi et al. 2016, Mistry et al. 2018, Seto et al. 2017 | 3 | 10 | 1 | 31 | 1 | 1 | - | 3 | - | 2 | 1 | 2 | 1 | 10 | 2 |
| **HBB 2nd edition** | 2016 | N.A. | Bameri et al. 2024, Chan et al. 2023, Chaulagain et al. 2021, Cordova et al. 2018, Data et al. 2022, Draiko et al. 2019, Draiko et al. 2019, Drake et al. 2019, Eblovi et al. 2017, Ezenwa et al. 2022, Heard Stittum et al. 2023, Hosseini et al. 2023, Kalabamu et al 2025, Kfoury et al. 2024, Jones-Bamman et al. 2019, Mediratta et al. 2025, Mubeen et al. 2021, Munyaw et al. 2022, Sendo et al. 2025, Sihota et al. 2025, Umoren et al. 2021, Zujkowski et al. 2025 | Hatamleh et al. 2021, Odongkara et al. 2020 | 3 | 9 | 1 | 35 | 1 | 1 | - | 3 | - | 2 | 1 | 2 | 2 | 10 | 2 |
| **ERC guideline 2021** | 2021 | N.A. |  |  | 1 | 1 | 1 | 14 | 1 | 1 | 4 | 4 | 5 | 1 | 2 | 2 | 1 | 10 | 2 |
| **Athanasopoulou** | 2024 | USA |  |  | 2 | 4 | 2 | 5 | 9 | 3 | 4 | 3 | 2 | 1 | 3 | 2 | 1 | 10 | 2 |
| **Barbato** | 2020 | USA |  |  | 2 |  | 1 | 36 | 1 | 2 | 1 | 4 | 3 | 1 | 3 | 2 | 1 | 2 | 1 |
| **Barry** | 2012 | USA |  |  | 2 | 6 | 1 | 133 | 1 | 2 | 2 | 3 | 3 | 1 | 3 | 2 | 1 | 1 | 1 |
| **Bender** | 2014 | USA |  |  | 2 | 8 | 2 | 55 | 7 | 2 | 1 | 1 | 2 | 1 | 3 | 2 | 1 | 1 | 2 |
| **Bibl** | 2023 | Austria |  |  | 1 | 1 | 1 | 38 | 7 | 1 | 1 | 4 | 2 | 2 | 2 | 1 | 1 | 2 | 1 |
| **Billner-Garcia** | 2024 | USA |  |  | 2 | 4 | 2 | 22 | 1 | 2 | 4 | 4 | 2 | 2 | 3 | 2 | 1 | 4 | 1 |
| **Binkhorst** | 2020 | The Netherlands |  |  | 1 | 3 | 4 | 20 | 7 | 2 | 1 | 1 | 3 | 1 | 3 | 2 | 2 | 10 | 2 |
| **Brathwaite** | 2020 | Ghana |  |  | 2 | 6 | 2 | 24 | 1 | 3 | 1 | 4 | 2 | 1 | 3 | 2 | 2 | 1,2,5 | 1 |
| **Brogaard** | 2022 | Denmark |  |  | 1 | 1, 2 | 1 | 29 | 2 | 1 | 1 | 5 | 3 | 1 | 2 | 2 | 2 | 2 | 1 |
| **Caldelari** | 2019 | Switzerland |  |  | 4 | 11 | 2 | 48 | 1 | 2 | 1 | 3 | 2 | 1 | 3 | 2 | 1 | 2,7 | 2 |
| **Campbell** | 2009 | Canada |  |  | 2 | 8 | 1 | 17 | 2 | 2 | 1 | 1.2 | 2 | 1 | 3 | 2 | 1 | 2 | 1 |
| **Carbine** | 2000 | USA | Cavicchiolo et al. 2018 |  | 2 | 7 | 1 | 42 | 1 | 2 | 1 | 5 | 2 | 2 | 3 | 2 | 2 | 2,5,9 | 1 |
| **Carlo** | 2009 | Zambia |  |  | 2 | 7 | 2 | 29 | 1 | 2 | 2 | 4 | 2 | 1 | 3 | 2 | 2 | 10 | 1 |
| **Cavallin** | 2021 | Mozambique, Italy |  |  | 2 | 12 | 1 | 19 | 2 | 2 | 1 | 4 | 4 | 1 | 1 | 2 | 1 | 2 | 1 |
| **Chan** | 2019 | Canada | Benguigui et al. 2024 |  | 2 | 6 | 2 | 15 | 2 | 1 | 1 | 1 | 2 | 1 | 2 | 2 | 1 | 2,4 | 1 |
| **Clark** | 2022 | Nepal |  |  | 3 | 9 | 2 | 22 | 1 | 2 | 2 | 5 | 2 | 2 | 3 | 2 | 2 | 1 | 1 |
| **Cordero** | 2013 | USA |  |  | 2 | 6 | 1 | 20 | 1 | 2 | 1 | 1 | 2 | 1 | 3 | 2 | 1 | 2,4 | 1 |
| **Cordero** | 2014 | USA |  |  | 2 | 6 | 1 | 20 | 1 | 2 | 1 | 1 | 2 | 1 | 3 | 2 | 1 | 2,4 | 1 |
| **Cronin** | 2001 | Canada |  |  | 2 | 11 | 2 | 19 | 1 | 2 | 1 | 4 | 2 | 2 | 3 | 2 | 1 | 1 | 1 |
| **Curran** | 2014 | Canada |  |  | 2 | 6 | 2 | 17 | 2 | 2 | 4 | 1.3 | 2 | 1 | 2 | 2 | 2 | 1 | 2 |
| **Cusack** | 2012 | UK |  |  | 1 | 12 | 1 | 43 | 7 | 2 | 2 | 1.3 | 2 | 2 | 3 | 2 | 1 | 1 | 1 |
| **Das** | 2018 | India |  |  | 2 | 12 | 1 | 26 | 1 | 2 | 2 | 4 | 2 | 1 | 3 | 2 | 2 | 2 | 1 |
| **De Bernardo** | 2016 | Italy |  |  | 2 | 6 | 2 | 22 | 1 | 2 | 1.2 | 1 | 2 | 1 | 3 | 2 | 1 | 1,2,5 | 2 |
| **Delaney** | 2022 | India |  |  | 4 | 11 | 1 | 15 | 1 | 2 | 2 | 4 | 2 | 2 | 3 | 2 | 2 | 10 | 1 |
| **Ding** | 2021 | Tanzania |  |  | 2 | 5 | 1 | 25 | 2 | 2 | 2 | 4 | 2 | 1 | 2 | 2 | 2 | 1 | 1 |
| **Farhadi** | 2023 | Iran |  |  | 2 | 4 | 1 | 19 | 2 | 2 | 1 | 3 | 2 | 1 | 2 | 2 | 1 | 2 | 2 |
| **Ford** | 2023 | USA |  |  | 2 | 5 | 1 | 13 | 1 | 2 | 2 | 5 | 2 | 1 | 1 | 2 | 2 | 2,4,5 | 2 |
| **Gamboa** | 2018 | Colombia |  |  | 5 | 12 | 1 | 41 | 2 | 2 | 1 | 4 | 2 | 2 | 2 | 2 | 1 | 10 | 2 |
| **Gelbart** | 2010 | Australia |  |  | 4 | 11 | 2 | 46 | 2 | 2 | 1 | 5 | 2.4 | 2 | 2 | 2 | 2 | 2 | 1 |
| **Ghoman** | 2020 | Canada |  |  | 2 | 5 | 1 | 40 | 1 | 2 | 1 | 4 | 2 | 2 | 3 | 2 | 2 | 10 | 1 |
| **Greer** | 2023 | USA |  |  | 2 | 5 | 1 | 13 | 1 | 2 | 1.2 | 4 | 2 | 1 | 3 | 2 | 1 | 2,4,5 | 2 |
| **Gross** | 2020 | USA |  |  | 2 | 5 | 4 | 19 | 9 | 3 | 1 | 1 | 2 | 1 | 3 | 2 | 2 | 10 | 2 |
| **Holm-Hansen** | 2022 | Tanzania | Holm-Hansen et al. 2024 |  | 5 | 12 | 1 | 6 | 8 | 2 | 1 | 5 | 4 | 1 | 3 | 2 | 2 | 9 | 2 |
| **Horiuchi** | 2021 | Laos |  |  | 5 | 12 | 1 | 53 | 2 | 2 | 4 | 4 | 2 | 2 | 2 | 2 | 2 | 10 | 1 |
| **Hundscheid** | 2021 | The Netherlands |  |  | 1 | 2 | 1 | 18 | 1 | 2 | 2 | 4 | 2 | 2 | 3 | 2 | 1 | 1 | 2 |
| **Johnson** | 2020 | USA |  |  | 2 | 5 | 1 | 20 | 3 | 2 | 2 | 1 | 2 | 1 | 2 | 2 | 1 | 2,9 | 2 |
| **Kamath-Rayne** | 2019 | USA |  |  | 3 | 10 | 2 | 21 | 2 | 1 | 2 | 3 | 2 | 2 | 1 | 2 | 1 | 1 | 2 |
| **Kamau** | 2022 | Kenya |  |  | 2 | 6 | 1 | 8 | 2 | 1 | 2 | 4 | 2 | 2 | 2 | 2 | 1 | 2 | 2 |
| **Kannan Loganathan** | 2024 | UK |  |  | 1 | 4 | 2 | 41 | 3 | 2 | 2 | 3 | 2 | 2 | 2 | 2 | 1 | 1 | 1 |
| **Kane** | 2019 | USA |  |  | 2 | 5 | 1 | 10 | 1 | 2 | 1 | 1 | 2 | 1 | 3 | 1 | 1 | 2,4,9 | 2 |
| **Khriesat** | 2017 | Jordan |  |  | 4 | 11 | 1 | 14 | 8 | 2 | 2 | 4 | 2 | 1 | 2 | 2 | 2 | 1 | 2 |
| **Kim** | 2013 | Afghanistan |  |  | 5 | 12 | 1 | 39 | 9 | 2 | 2 | 4 | 2 | 2 | 3 | 2 | 2 | 2,8 | 1 |
| **Lizotte** | 2017 | Canada |  |  | 2 | 6 | 1 | 28 | 2 | 1 | 1 | 1 | 2 | 1 | 1 | 2 | 1 | 1,9 | 1 |
| **Lockyer** | 2006 | Canada, USA | Bang et al. 2016, Charafeddine et al. 2016, Lizotte et al. 2015, Nimbalkar et al. 2015, Patel et al. 2012, Weiner et al. 2006, NRP 6th edition megacode score | Bould et al. 2009, Katakam et al. 2012 | 2 | 7 | 1 | 20 | 2 | 1 | 1 | 4 | 2 | 1 | 2 | 1 | 2 | 1 | 2 |
| **Magee** | 2018 | USA |  |  | 2 | 5 | 2 | 60 | 2 | 2 | 1 | 1 | 2 | 2 | 3 | 2 | 1 | 2 | 2 |
| **Maya-Enero** | 2018 | Spain |  |  | 4 | 11 | 1 | 12 | 2 | 2 | 1 | 5 | 2 | 1 | 2 | 2 | 2 | 10 | 2 |
| **McCaw** | 2023 | Philippines |  |  | 3 | 9 | 2 | 21 | 1.8 | 1 | 1.2 | 3 | 2 | 2 | 1 | 2 | 1 | 1 | 2 |
| **Meggiolaro** | 2025 | Italy |  |  | 2 | 4 | 1 | 33 | 3 | 1 | 1 | 1 | 2 | 1 | 1 | 1 | 1 | 2 | 1 |
| **Mersha** | 2019 | Ethiopia |  |  | 3 | 9 | 2 | 39 | 1 | 2 | 4 | 4 | 2 | 1 | 3 | 2 | 2 | 10 | 1 |
| **Mileder** | 2023 | Austria |  |  | 1 | 1 | 1 | 5 | 2 | 2 | 1 | 3 | 2 | 1 | 2 | 2 | 1 | 2 | 2 |
| **Nadler** | 2011 | Australia |  |  | 2 | 8 | 1 | 5 | 9 | 2 | 1 | 5 | 2 | 1 | 3 | 2 | 2 | 2, 9 | 2 |
| **Nickerson** | 2019 | USA |  |  | 2 | 5 | 1 | 5 | 2 | 2 | 2 | 1 | 3 | 1 | 1 | 2 | 1 | 10 | 2 |
| **Nvonako** | 2022 | Cameroon |  |  | 4 | 11 | 1 | 11 | 1 | 1 | 2 | 4 | 2 | 1 | 3 | 2 | 1 | 1, 2, 9 | 2 |
| **Paliatsiou** | 2021 | Greece |  |  | 1 | 1 | 1 | 21 | 1 | 2 | 2 | 1 | 2 | 1 | 2 | 2 | 2 | 10 | 2 |
| **Reisman** | 2015 | Tanzania | Arlington et al. 2017, Chinbuah et al. 2020 |  | 3 | 10 | 1 | 13 | 1 | 1 | 2 | 3 | 2 | 2 | 1 | 1 | 1 | 1,2 | 1 |
| **Roitsch** | 2020 | USA | Neveln et al. 2023 |  | 2 | 5 | 1 | 34 | 3 | 2 | 1 | 1 | 2 | 2 | 2 | 1 | 1 | 2 | 1 |
| **Rovamo** | 2013 | Finland |  |  | 4 | 11 | 1 | 30 | 1 | 2 | 1, 2 | 1 | 2 | 1 | 2 | 2 | 1 | 1, 2, 9 | 2 |
| **Rovamo** | 2011 | Finland | Liaqat et al. 2021 |  | 4 | 11 | 1 | 30 | 1 | 2 | 1, 2 | 1 | 2 | 1 | 2 | 2 | 1 | 1, 2, 9 | 2 |
| **Sarvan** | 2022 | Turkey |  |  | 2 | 5 | 1 | 12 | 9 | 3 | 3 | 4 | 2 | 1 | 3 | 2 | 1 | 10 | 2 |
| **Sawyer** | 2011 | US | Hadfield et al. 2024, Saywer et al. 2012, Sawyer et al. 2014, Litke-Wager et al. 2021 | Matterson et al. 2018 | 2 | 6 | 2 | 30 | 1 | 2 | 1 | 1 | 3 | 2 | 2 | 2 | 1 | 2 | 1 |
| **Shikuku** | 2017 | Kenya |  |  | 4 | 11 | 1 | 20 | 1 | 2 | 2 | 5 | 2 | 1 | 3 | 2 | 2 | 5 | 1 |
| **Sinha** | 2024 | India | Sinha et al. 2025 |  | 4 | 11 | 1 | 14 | 2 | 1 | 1, 2 | 3 | 2 | 1 | 2 | 2 | 2 | 2 | 2 |
| **Sintayehu** | 2020 | Ethiopia |  |  | 4 | 11 | 1 | 38 | 2 | 2 | 4 | 4 | 2 | 2 | 2 | 2 | 2 | 9 | 1 |
| **Skâre** | 2018 | Norway | Skare et al. 2018 |  | 4 | 11 | 2 | 24 | 2 | 2 | 1 | 5 | 3 | 1 | 2 | 2 | 2 | 9 | 1 |
| **Sloane** | 2021 | USA |  |  | 2 | 5 | 2 | 53 | 2 | 2 | 1 | 5 | 2 | 2 | 3 | 2 | 2 | 9 | 1 |
| **Szyld** | 2021 | Argentina, USA |  |  | 2 | 5 | 1 | 13 | 2 | 2 | 1 | 4 | 2 | 2 | 2 | 2 | 1 | 1 | 2 |
| **Tosif** | 2020 | The Solomon Islands |  |  | 4 | 11 | 1 | 30 | 9 | 3 | 4 | 4 | 2 | 1 | 3 | 2 | 1 | 9 | 1 |
| **Trevisanuto** | 2015 | Mozambique |  |  | 2 | 6 | 2 | 19 | 2 | 2 | 1 | 5 | 2 | 1 | 2 | 2 | 2 | 2 | 1 |
| **Tsang** | 2022 | The Netherlands |  |  | 1 | 2 | 1 | 40 | 1 | 2 | 1 | 1 | 2 | 2 | 3 | 2 | 1 | 1, 3 | 1 |
| **Vail** | 2018 | India |  |  | 4 | 11 | 1 | 10 | 1, 7 | 2 | 1 | 3 | 2 | 1 | 3 | 2 | 1 | 10 | 1 |
| **Vail** | 2017 | India |  |  | 4 | 11 | 1 | 10 | 1, 7 | 2 | 1 | 3 | 2 | 1 | 3 | 2 | 1 | 5 | 2 |
| **Van der Heide** | 2006 | The Netherlands | Finan et al. 2011, Louvel et al. 2024, Michelet et al. 2020 | Lee et al. 2012 | 2 | 7 | 2 | 44 | 1 | 1 | 1 | 2 | 2 | 2 | 2 | 1 | 1 | 2, 3 | 1 |
| **Woods** | 2015 | Cambodia |  |  | 4 | 11 | 1 | 15 | 7 | 1 | 2 | 3 | 2 | 1 | 3 | 2 | 2 | 10 | 2 |
| **Xu** | 2023 | China |  |  | 2, 4 | 5, 11 | 2 | 36 | 2 | 1 | 2 | 1 | 2 | 2 | 2 | 2 | 2 | 10 | 1 |
| **Yamada** | 2016 | USA |  |  | 2 | 5 | 2 | 46 | 8 | 2 | 1 | 1 | 2 | 2 | 2 | 2 | 1 | 1 | 1 |
| **Yamada** | 2015 | USA |  |  | 2 | 6 | 1 | 55 | 8 | 2 | 1 | 5 | 4 | 2 | 2 | 2 | 2 | 10 | 2 |
| **Yaylaci** | 2021 | Turkey |  |  | 2 | 5 | 1 | 35 | 9 | 2 | 1 | 1 | 2 | 2 | 3 | 2 | 1 | 10 | 1 |
| **Yeo** | 2020 | Singapore |  |  | 4 | 11 | 1 | 23 | 1, 2 | 1 | 2 | 1 | 2 | 2 | 2 | 2 | 1 | 10 | 1 |
| **Yoosoof** | 2022 | Sri Lanka |  |  | 4 | 11 | 1 | 30 | 3 | 2 | 2 | 2, 3 | 2 | 1 | 3 | 2 | 1 | 1 | 1 |

Abbreviations used in this table:

AHA: American Heart Association

ERC: European Resuscitation Council

HBB: Helping Babies Breathe

N/A: not applicable

NLS: Newborn/Neonatal life support

NRP: Neonatal Resuscitation Program

**Table 2: Overview of assessment tool items mapped to the ERC and AHA resuscitation algorithms**

Part 1: preparation and first steps

|  | | **Preparation** | | | | | | | | | | | | | **First steps and temperature management** | | | | | | | | | |
| --- | --- | --- | --- | --- | --- | --- | --- | --- | --- | --- | --- | --- | --- | --- | --- | --- | --- | --- | --- | --- | --- | --- | --- | --- |
| **First author** | **Year of publication** | **Team briefing** | **Put on gloves** | **Turn on heater** | **Collect warm, clean towels** | **Put T-piece and ventilation device at resuciscitation table and check functionality** | **Check if appropiate mask size is available** | **Prepare and test the suction unit** | **Prepare monitor devices (ECG and/or SpO2)** | **Check if stethoscope is present at resuscitation table** | **Check if medication and fluids are available** | **Check if equipment for iv access is availble** | **Check if alternative airway devices are available** | **Equipment/material check not further specified** | **Delayed cord clamping (if possible)** | **Start Apgar clock** | **Dry the baby / When preterm GA <32 weeks: place undried in plastic wrap** | **Remove wet towels** | **Cover baby with dry towels** | **Tactile stimulation** | **Assess heart rate** | **Assess breathing** | **Assess tone** | **Adequate open airway manoeu-vre** |
|  |  | **1 = Yes 2 = No** | **1 = Yes 2 = No** | **1 = Yes 2 = No** | **1 = Yes 2 = No** | **1 = Yes 2 = No** | **1 = Yes 2 = No** | **1 = Yes 2 = No** | **1 = Yes 2 = No** | **1 = Yes 2 = No** | **1 = Yes 2 = No** | **1 = Yes 2 = No** | **1 = Yes 2 = No** | **1 = Yes 2 = No** | **1 = Yes 2 = No** | **1 = Yes 2 = No** | **1 = Yes 2 = No** | **1 = Yes 2 = No** | **1 = Yes 2 = No** | **1= Yes 2= No** | **1 = Yes 2 = No** | **1 = Yes 2 = No** | **1 = Yes 2 = No** | **1 = Yes 2 = No** |
| **ERC 2021** | 2021 | 2 | 2 | 2 | 2 | 2 | 2 | 2 | 2 | 2 | 2 | 2 | 2 | 2 | 2 | 1 | 1 | 1 | 1 | 2 | 1 | 1 | 2 | 1 |
| **HBB 1st edition** | 2010 | 2 | 2 | 2 | 2 | 1 | 1 | 1 | 2 | 2 | 2 | 2 | 2 | 1 | 2 | 2 | 1 | 2 | 2 | 1 | 2 | 2 | 2 | 1 |
| **HBB 2nd edition** | 2016 | 2 | 2 | 2 | 1 | 1 | 2 | 1 | 2 | 2 | 2 | 2 | 2 | 2 | 1 | 2 | 1 | 1 | 1 | 1 | 2 | 1 | 2 | 1 |
| **Athanasopoulou** | 2024 | 2 | 2 | 2 | 2 | 2 | 2 | 2 | 2 | 2 | 2 | 2 | 2 | 2 | 2 | 2 | 1 | 2 | 2 | 1 | 2 | 2 | 2 | 2 |
| **Barbato** | 2020 | 1 | 2 | 1 | 1 | 1 | 2 | 1 | 2 | 2 | 2 | 2 | 1 | 2 | 2 | 1 | 1 | 2 | 2 | 1 | 1 | 1 | 2 | 1 |
| **Barry** | 2012 | 2 | 2 | 1 | 2 | 1 | 1 | 1 | 2 | 2 | 1 | 1 | 1 | 2 | 2 | 1 | 2 | 2 | 2 | 2 | 2 | 2 | 2 | 2 |
| **Bender** | 2014 | 2 | 1 | 1 | 2 | 1 | 2 | 1 | 2 | 2 | 2 | 2 | 1 | 2 | 2 | 2 | 1 | 1 | 1 | 2 | 1 | 1 | 2 | 1 |
| **Bibl** | 2023 | 2 | 1 | 1 | 1 | 1 | 1 | 1 | 1 | 1 | 1 | 1 | 1 | 2 | 2 | 1 | 1 | 1 | 1 | 1 | 1 | 1 | 1 | 1 |
| **Billner-Garcia** | 2022 | 1 | 2 | 1 | 1 | 2 | 2 | 2 | 2 | 2 | 2 | 2 | 2 | 1 | 2 | 2 | 1 | 1 | 2 | 1 | 2 | 1 | 2 | 1 |
| **Binkhorst** | 2020 | 2 | 2 | 2 | 2 | 2 | 2 | 2 | 2 | 2 | 2 | 2 | 2 | 2 | 2 | 2 | 1 | 1 | 1 | 2 | 1 | 2 | 2 | 1 |
| **Brathwaite** | 2020 | 2 | 1 | 1 | 1 | 1 | 1 | 1 | 2 | 2 | 2 | 2 | 2 | 2 | 2 | 2 | 2 | 2 | 2 | 1 | 1 | 1 | 2 | 2 |
| **Brogaard** | 2022 | 2 | 2 | 1 | 1 | 1 | 2 | 1 | 1 | 1 | 2 | 2 | 1 | 2 | 2 | 2 | 1 | 1 | 2 | 2 | 1 | 1 | 1 | 1 |
| **Caldelari** | 2019 | 1 | 2 | 1 | 1 | 1 | 1 | 1 | 1 | 1 | 2 | 2 | 2 | 2 | 2 | 1 | 1 | 1 | 2 | 1 | 1 | 2 | 2 | 1 |
| **Campbell** | 2009 | 2 | 2 | 2 | 2 | 1 | 2 | 1 | 2 | 2 | 2 | 2 | 1 | 2 | 2 | 2 | 2 | 2 | 2 | 2 | 2 | 2 | 2 | 2 |
| **Carbine** | 2000 | 1 | 2 | 2 | 2 | 2 | 1 | 2 | 2 | 2 | 2 | 2 | 2 | 1 | 2 | 2 | 1 | 1 | 2 | 2 | 1 | 2 | 2 | 1 |
| **Carlo** | 2009 | 2 | 2 | 1 | 2 | 1 | 1 | 1 | 2 | 2 | 1 | 2 | 2 | 1 | 2 | 2 | 1 | 1 | 2 | 1 | 2 | 2 | 2 | 1 |
| **Cavallin** | 2021 | 2 | 2 | 2 | 2 | 1 | 2 | 1 | 2 | 2 | 2 | 2 | 2 | 2 | 2 | 2 | 1 | 1 | 2 | 1 | 1 | 2 | 2 | 1 |
| **Chan** | 2019 | 2 | 2 | 2 | 2 | 1 | 1 | 2 | 2 | 2 | 2 | 2 | 2 | 2 | 2 | 2 | 1 | 1 | 2 | 2 | 1 | 1 | 2 | 1 |
| **Clark** | 2022 | 1 | 2 | 2 | 2 | 1 | 2 | 1 | 2 | 2 | 2 | 2 | 2 | 1 | 2 | 2 | 1 | 2 | 2 | 1 | 2 | 1 | 2 | 1 |
| **Cordero** | 2013 | 2 | 2 | 2 | 2 | 2 | 2 | 2 | 2 | 2 | 2 | 2 | 2 | 2 | 2 | 2 | 1 | 2 | 2 | 1 | 1 | 1 | 2 | 2 |
| **Cordero** | 2014 | 2 | 2 | 2 | 2 | 2 | 2 | 2 | 2 | 2 | 2 | 2 | 2 | 2 | 2 | 2 | 1 | 2 | 2 | 1 | 1 | 1 | 2 | 2 |
| **Cronin** | 2001 | 2 | 2 | 2 | 2 | 2 | 2 | 2 | 2 | 2 | 2 | 2 | 2 | 1 | 2 | 2 | 1 | 1 | 2 | 1 | 1 | 1 | 2 | 1 |
| **Curran** | 2014 | 2 | 2 | 2 | 2 | 1 | 2 | 2 | 2 | 2 | 2 | 2 | 2 | 2 | 2 | 2 | 1 | 1 | 2 | 2 | 1 | 1 | 1 | 1 |
| **Cusack** | 2012 | 2 | 2 | 1 | 1 | 1 | 1 | 1 | 2 | 1 | 2 | 2 | 1 | 1 | 2 | 1 | 1 | 1 | 1 | 2 | 1 | 1 | 1 | 1 |
| **Das** | 2018 | 1 | 2 | 1 | 2 | 1 | 1 | 2 | 2 | 2 | 2 | 2 | 2 | 2 | 2 | 2 | 1 | 1 | 1 | 1 | 1 | 1 | 2 | 1 |
| **De Bernardo** | 2016 | 2 | 2 | 2 | 2 | 2 | 1 | 1 | 2 | 2 | 2 | 2 | 2 | 2 | 2 | 2 | 1 | 1 | 2 | 1 | 1 | 2 | 2 | 1 |
| **Delaney** | 2022 | 2 | 2 | 2 | 2 | 1 | 1 | 1 | 2 | 1 | 2 | 2 | 2 | 2 | 2 | 2 | 1 | 2 | 1 | 1 | 2 | 1 | 2 | 1 |
| **Ding** | 2021 | 2 | 2 | 2 | 2 | 1 | 1 | 2 | 2 | 2 | 2 | 2 | 2 | 2 | 2 | 2 | 1 | 1 | 2 | 1 | 1 | 1 | 1 | 1 |
| **Farhadi** | 2023 | 1 | 2 | 2 | 2 | 1 | 2 | 2 | 2 | 2 | 2 | 2 | 2 | 1 | 2 | 2 | 1 | 1 | 2 | 1 | 1 | 1 | 2 | 1 |
| **Ford** | 2023 | 2 | 2 | 1 | 2 | 2 | 2 | 2 | 2 | 2 | 2 | 2 | 2 | 2 | 2 | 2 | 1 | 2 | 2 | 1 | 1 | 1 | 2 | 1 |
| **Gamboa** | 2018 | 2 | 2 | 2 | 2 | 2 | 2 | 2 | 2 | 2 | 2 | 2 | 2 | 2 | 2 | 2 | 1 | 1 | 2 | 1 | 1 | 2 | 2 | 1 |
| **Gelbart** | 2010 | 1 | 2 | 2 | 2 | 2 | 2 | 2 | 2 | 2 | 2 | 2 | 2 | 1 | 2 | 2 | 1 | 1 | 2 | 1 | 1 | 2 | 2 | 1 |
| **Ghoman** | 2020 | 1 | 1 | 2 | 2 | 1 | 2 | 2 | 2 | 2 | 2 | 2 | 2 | 1 | 1 | 2 | 1 | 2 | 2 | 1 | 1 | 1 | 1 | 1 |
| **Greer** | 2023 | 1 | 2 | 1 | 1 | 1 | 2 | 1 | 2 | 2 | 2 | 2 | 1 | 1 | 2 | 2 | 1 | 1 | 2 | 1 | 1 | 1 | 1 | 1 |
| **Gross** | 2020 | 2 | 2 | 1 | 2 | 2 | 2 | 2 | 2 | 2 | 2 | 2 | 2 | 2 | 2 | 2 | 1 | 2 | 2 | 1 | 1 | 1 | 2 | 1 |
| **Holm-Hansen** | 2022 | 2 | 2 | 2 | 2 | 2 | 2 | 2 | 2 | 2 | 2 | 2 | 2 | 2 | 2 | 2 | 1 | 1 | 1 | 1 | 2 | 2 | 2 | 1 |
| **Horiuchi** | 2021 | 2 | 1 | 2 | 2 | 1 | 2 | 1 | 2 | 2 | 2 | 2 | 2 | 1 | 1 | 2 | 1 | 1 | 1 | 2 | 2 | 1 | 2 | 1 |
| **Hundscheid** | 2021 | 2 | 2 | 1 | 2 | 2 | 2 | 2 | 2 | 2 | 2 | 2 | 2 | 2 | 2 | 1 | 1 | 1 | 1 | 2 | 1 | 1 | 2 | 1 |
| **Johnson** | 2020 | 1 | 2 | 2 | 2 | 2 | 2 | 2 | 2 | 2 | 2 | 2 | 2 | 2 | 2 | 2 | 2 | 2 | 2 | 2 | 2 | 2 | 2 | 2 |
| **Kamath-Rayne** | 2019 | 1 | 1 | 2 | 2 | 1 | 2 | 2 | 2 | 2 | 2 | 2 | 2 | 1 | 2 | 2 | 1 | 1 | 2 | 1 | 2 | 1 | 2 | 1 |
| **Kamau** | 2022 | 2 | 2 | 2 | 2 | 2 | 2 | 2 | 2 | 2 | 2 | 2 | 2 | 2 | 2 | 2 | 1 | 1 | 1 | 1 | 1 | 1 | 1 | 1 |
| **Kane** | 2019 | 1 | 2 | 2 | 2 | 2 | 2 | 2 | 2 | 2 | 2 | 2 | 2 | 1 | 1 | 2 | 1 | 2 | 2 | 1 | 1 | 1 | 2 | 2 |
| **Kannan Loganathan** | 2024 | 1 | 2 | 2 | 2 | 2 | 2 | 2 | 2 | 2 | 2 | 2 | 2 | 1 | 2 | 1 | 1 | 1 | 2 | 1 | 1 | 1 | 1 | 1 |
| **Khriesat** | 2017 | 2 | 2 | 1 | 2 | 2 | 2 | 2 | 2 | 2 | 2 | 2 | 2 | 2 | 2 | 2 | 1 | 2 | 1 | 2 | 1 | 1 | 1 | 2 |
| **Kim** | 2013 | 2 | 1 | 2 | 2 | 2 | 2 | 2 | 2 | 2 | 2 | 2 | 2 | 1 | 2 | 2 | 1 | 1 | 1 | 2 | 2 | 1 | 2 | 1 |
| **Lizotte** | 2017 | 1 | 2 | 2 | 2 | 1 | 1 | 2 | 2 | 2 | 2 | 2 | 2 | 2 | 2 | 2 | 1 | 1 | 2 | 2 | 1 | 1 | 2 | 1 |
| **Lockyer** | 2006 | 2 | 2 | 1 | 1 | 1 | 2 | 2 | 2 | 2 | 2 | 2 | 2 | 2 | 2 | 2 | 1 | 1 | 2 | 2 | 1 | 1 | 2 | 1 |
| **Magee** | 2018 | 1 | 1 | 1 | 2 | 1 | 2 | 1 | 1 | 1 | 1 | 1 | 1 | 2 | 2 | 1 | 1 | 1 | 2 | 1 | 1 | 2 | 2 | 2 |
| **Maya-Enero** | 2018 | 2 | 2 | 2 | 2 | 2 | 2 | 2 | 2 | 2 | 2 | 2 | 2 | 2 | 2 | 2 | 1 | 1 | 1 | 1 | 2 | 2 | 2 | 1 |
| **McCaw** | 2023 | 2 | 1 | 2 | 2 | 1 | 2 | 2 | 2 | 2 | 2 | 2 | 2 | 1 | 1 | 2 | 1 | 1 | 1 | 1 | 2 | 1 | 2 | 1 |
| **Meggiolaro** | 2025 | 1 | 2 | 1 | 1 | 1 | 2 | 1 | 1 | 2 | 1 | 1 | 1 | 2 | 2 | 2 | 1 | 2 | 2 | 1 | 1 | 1 | 2 | 1 |
| **Mersha** | 2019 | 2 | 1 | 2 | 2 | 2 | 2 | 2 | 2 | 2 | 2 | 2 | 2 | 1 | 2 | 2 | 1 | 1 | 1 | 2 | 2 | 1 | 2 | 1 |
| **Mileder** | 2023 | 2 | 2 | 1 | 2 | 2 | 2 | 2 | 2 | 2 | 2 | 2 | 2 | 2 | 2 | 2 | 1 | 1 | 1 | 1 | 1 | 2 | 2 | 1 |
| **Nadler** | 2011 | 2 | 2 | 1 | 2 | 2 | 2 | 2 | 2 | 2 | 2 | 2 | 2 | 2 | 2 | 2 | 1 | 2 | 2 | 2 | 2 | 2 | 2 | 1 |
| **Nickerson** | 2019 | 2 | 2 | 2 | 2 | 2 | 1 | 2 | 2 | 2 | 2 | 2 | 2 | 2 | 2 | 2 | 1 | 1 | 2 | 1 | 1 | 1 | 2 | 2 |
| **Nvonako** | 2022 | 2 | 2 | 1 | 1 | 2 | 2 | 2 | 2 | 2 | 2 | 2 | 2 | 2 | 2 | 1 | 1 | 1 | 1 | 1 | 2 | 1 | 2 | 1 |
| **Paliatsiou** | 2021 | 2 | 2 | 2 | 2 | 1 | 1 | 2 | 2 | 2 | 2 | 2 | 2 | 2 | 2 | 2 | 2 | 2 | 2 | 2 | 2 | 2 | 2 | 1 |
| **Reisman** | 2015 | 2 | 1 | 2 | 2 | 2 | 2 | 2 |  | 2 | 2 | 2 | 2 | 1 | 1 | 2 | 1 | 1 | 1 | 1 | 2 | 1 | 2 | 1 |
| **Roitsch** | 2020 | 1 | 2 | 2 | 2 | 2 | 2 | 2 | 2 | 2 | 2 | 2 | 2 | 1 | 2 | 2 | 1 | 1 | 2 | 1 | 1 | 1 | 2 | 1 |
| **Rovamo** | 2013 | 2 | 2 | 1 | 2 | 1 | 1 | 1 | 1 | 2 | 1 | 2 | 1 | 2 | 2 | 2 | 1 | 1 | 2 | 1 | 1 | 2 | 2 | 1 |
| **Rovamo** | 2011 | 2 | 2 | 2 | 2 | 2 | 2 | 1 | 2 | 2 | 2 | 2 | 2 | 2 | 2 | 2 | 1 | 1 | 2 | 1 | 1 | 2 | 2 | 1 |
| **Sarvan** | 2022 | 2 | 2 | 2 | 2 | 2 | 2 | 2 | 2 | 2 | 2 | 2 | 2 | 2 | 2 | 2 | 2 | 2 | 2 | 2 | 2 | 2 | 2 | 1 |
| **Sawyer** | 2011 | 2 | 2 | 2 | 2 | 2 | 2 | 2 | 2 | 2 | 2 | 2 | 2 | 1 | 2 | 2 | 1 | 2 | 2 | 1 | 1 | 2 | 2 | 1 |
| **Shikuku** | 2017 | 2 | 2 | 2 | 2 | 2 | 2 | 2 | 2 | 2 | 2 | 2 | 2 | 1 | 2 | 2 | 1 | 1 | 1 | 2 | 2 | 2 | 2 | 1 |
| **Sinha** | 2024 | 2 | 2 | 2 | 2 | 2 | 2 | 2 | 2 | 2 | 2 | 2 | 2 | 1 | 2 | 2 | 1 | 1 | 2 | 1 | 2 | 1 | 2 | 1 |
| **Sintayehu** | 2020 | 2 | 1 | 2 | 2 | 2 | 2 | 2 | 2 | 2 | 2 | 2 | 2 | 1 | 2 | 2 | 1 | 2 | 1 | 2 | 2 | 1 | 2 | 1 |
| **Skâre** | 2018 | 1 | 2 | 2 | 2 | 2 | 2 | 2 | 2 | 2 | 2 | 2 | 2 | 1 | 2 | 2 | 1 | 1 | 2 | 1 | 1 | 2 | 2 | 1 |
| **Sloane** | 2021 | 1 | 2 | 2 | 2 | 2 | 2 | 2 | 2 | 2 | 2 | 2 | 2 | 1 | 2 | 2 | 1 | 1 | 2 | 1 | 1 | 2 | 2 | 1 |
| **Szyld** | 2021 | 2 | 2 | 1 | 1 | 1 | 2 | 1 | 2 | 1 | 2 | 2 | 2 | 2 | 2 | 2 | 1 | 1 | 1 | 2 | 1 | 1 | 1 | 1 |
| **Tosif** | 2020 | 2 | 1 | 2 | 2 | 1 | 2 | 2 | 2 | 2 | 2 | 2 | 2 | 1 | 2 | 2 | 1 | 1 | 1 | 2 | 2 | 1 | 2 | 1 |
| **Trevisanuto** | 2015 | 2 | 2 | 2 | 2 | 1 | 2 | 1 | 2 | 2 | 2 | 2 | 2 | 2 | 2 | 2 | 1 | 1 | 2 | 1 | 1 | 2 | 2 | 1 |
| **Tsang** | 2022 | 2 | 2 | 1 | 2 | 1 | 2 | 2 | 2 | 2 | 2 | 2 | 2 | 2 | 2 | 1 | 1 | 1 | 1 | 2 | 1 | 1 | 1 | 1 |
| **Vail** | 2018 | 2 | 2 | 2 | 2 | 2 | 2 | 2 | 2 | 2 | 2 | 2 | 2 | 2 | 2 | 2 | 2 | 2 | 2 | 1 | 1 | 2 | 2 | 1 |
| **Vail** | 2017 | 2 | 2 | 2 | 2 | 2 | 2 | 2 | 2 | 2 | 2 | 2 | 2 | 2 | 2 | 2 | 1 | 2 | 2 | 1 | 1 | 1 | 2 | 2 |
| **Van der Heide** | 2006 | 1 | 2 | 2 | 2 | 2 | 2 | 2 | 2 | 2 | 2 | 2 | 2 | 1 | 2 | 2 | 1 | 1 | 2 | 1 | 1 | 2 | 2 | 1 |
| **Woods** | 2015 | 2 | 2 | 2 | 2 | 2 | 1 | 2 | 2 | 2 | 2 | 2 | 2 | 2 | 2 | 2 | 2 | 2 | 2 | 2 | 2 | 2 | 2 | 2 |
| **Xu** | 2023 | 1 | 2 | 1 | 1 | 1 | 2 | 1 | 2 | 2 | 2 | 2 | 2 | 1 | 2 | 2 | 1 | 1 | 2 | 1 | 1 | 1 | 2 | 1 |
| **Yamada** | 2016 | 2 | 2 | 2 | 2 | 1 | 1 | 1 | 2 | 1 | 2 | 2 | 1 | 2 | 2 | 2 | 1 | 1 | 2 | 1 | 1 | 2 | 2 | 2 |
| **Yamada** | 2015 | 2 | 2 | 2 | 2 | 1 | 1 | 1 | 2 | 1 | 2 | 2 | 1 | 2 | 2 | 2 | 1 | 1 | 2 | 1 | 1 | 1 | 2 | 2 |
| **Yaylaci** | 2021 | 1 | 2 | 1 | 1 | 1 | 1 | 1 | 1 | 2 | 1 | 1 | 2 | 2 | 2 | 2 | 1 | 1 | 2 | 1 | 2 | 1 | 2 | 1 |
| **Yeo** | 2020 | 2 | 2 | 2 | 2 | 2 | 2 | 2 | 2 | 2 | 2 | 2 | 2 | 2 | 2 | 1 | 1 | 1 | 2 | 1 | 1 | 1 | 1 | 1 |
| **Yoosoof** | 2022 | 2 | 1 | 2 | 2 | 2 | 2 | 2 | 2 | 2 | 2 | 2 | 2 | 2 | 2 | 2 | 2 | 2 | 2 | 2 | 1 | 1 | 1 | 1 |

Part 2: airway management 1

|  | | **ERC: If gasping/not breathing start with 5 inflations NRP: if gasping/not breathing or HR <100/min start PPV** | | | **ERC** | | | | **NRP** | | |  | | **Reassess: check effect** | | **Chest is not moving --> alternative airway management strategies** | | | | |
| --- | --- | --- | --- | --- | --- | --- | --- | --- | --- | --- | --- | --- | --- | --- | --- | --- | --- | --- | --- | --- |
| **First author** | **Year of publication** | **Correct placement of mask** | **CE-grip** | **O2 21-30% depending on GA** | **Give 5 inflation breaths** | **2-3 seconds/inflation** | **PEEP 5 cm H2O** | **PIP 25 - 30 cm H2O depending on GA** | **Start ventilation breaths for 30 seconds** | **Frequency 40-60 breaths / minute** | **PIP 20 – 30 cm H2O depending on GA** | **Consider using PEEP** | **Consider pulse oximetry measurement** | **Assess: chest movements** | **Assess: heart rate and breathing** | **Reposition: check mask, neutral position, jaw-thrust** | **Consider alternative ariway technique** | **Alternative strategies ^1^** | **Consider suction of the oropharynx under direct sight** | **Consider increasing inflation pressure** |
|  |  | **1 = Yes 2 = No** | **1 = Yes 2 = No** | **1 = Yes 2 = No** | **1 = Yes 2 = No 3 = Not applicable** | **1 = Yes 2 = No 3 = Not applicable** | **1 = Yes 2 = No** | **1 = Yes 2 = No 3 = Not applicable** | **1 = Yes 2 = No 3 = Not applicable** | **1 = Yes 2 = No 3 = Not applicable** | **1 = Yes 2 = No 3 = Not applicable** | **1 = Yes 2 = No 3 = Not applicable** | **1 = Yes 2 = No** | **1 = Yes 2 = No** | **1 = Yes 2 = No** | **1 = Yes 2 = No** | **1 = Yes 2 = No** | **1 = Yes 2 = No** | **1 = Yes 2 = No** | **1 = Yes 2 = No** |
| **ERC 2021** | 2021 | 1 | 2 | 2 | 1 | 1 | 2 | 2 | 3 | 3 | 3 | 3 | 3 | 1 | 1 | 1 | 1 | 1 | 2 | 2 |
| **HBB 1st edition** | 2010 | 1 | 2 | 2 | 3 | 3 | 3 | 3 | 1 | 1 | 2 | 2 | 2 | 2 | 1 | 2 | 2 | 2 | 1 | 2 |
| **HBB 2nd edition** | 2016 | 1 | 2 | 2 | 3 | 3 | 3 | 2 | 1 | 1 | 2 | 2 | 2 | 1 | 1 | 1 | 2 | 2 | 1 | 1 |
| **Athanasopoulou** | 2024 | 1 | 2 | 2 | 3 | 3 | 3 | 3 | 1 | 2 | 2 | 2 | 2 | 2 | 2 | 1 | 1 | 1 | 1 | 1 |
| **Barbato** | 2020 | 2 | 2 | 1 | 3 | 3 | 3 | 3 | 1 | 2 | 2 | 2 | 1 | 2 | 1 | 1 | 1 | 1 | 1 | 1 |
| **Barry** | 2012 | 2 | 2 | 1 | 3 | 3 | 1 | 3 | 1 | 2 | 2 | 2 | 1 | 2 | 2 | 2 | 1 | 1 | 1 | 2 |
| **Bender** | 2014 | 2 | 2 | 2 | 3 | 3 | 3 | 3 | 1 | 1 | 1 | 2 | 2 | 1 | 1 | 1 | 2 | 2 | 2 | 2 |
| **Bibl** | 2023 | 2 | 2 | 2 | 1 | 2 | 2 | 2 | 3 | 3 | 3 | 3 | 2 | 1 | 2 | 1 | 1 | 1 | 2 | 2 |
| **Billner-Garcia** | 2022 | 1 | 2 | 1 | 3 | 3 | 3 | 3 | 1 | 1 | 1 | 1 | 1 | 2 | 1 | 2 | 2 | 2 | 2 | 2 |
| **Binkhorst** | 2020 | 2 | 2 | 2 | 1 | 1 | 2 | 1 | 3 | 3 | 3 | 3 | 1 | 2 | 2 | 2 | 2 | 2 | 2 | 2 |
| **Brathwaite** | 2020 | 2 | 2 | 2 | 3 | 3 | 3 | 3 | 1 | 1 | 2 | 2 | 2 | 1 | 1 | 1 | 2 | 2 | 1 | 2 |
| **Brogaard** | 2022 | 2 | 2 | 2 | 1 | 1 | 2 | 2 | 3 | 3 | 3 | 3 | 2 | 2 | 1 | 1 | 1 | 1 | 1 | 2 |
| **Caldelari** | 2019 | 1 | 2 | 2 | 3 | 3 | 3 | 3 | 1 | 1 | 2 | 2 | 2 | 1 | 1 | 2 | 2 | 2 | 1 | 2 |
| **Campbell** | 2009 | 2 | 2 | 2 | 3 | 3 | 3 | 3 | 1 | 2 | 2 | 2 | 2 | 2 | 2 | 2 | 1 | 1 | 2 | 2 |
| **Carbine** | 2000 | 2 | 2 | 1 | 3 | 3 | 3 | 3 | 1 | 1 | 2 | 2 | 2 | 1 | 1 | 2 | 1 | 1 | 1 | 2 |
| **Carlo** | 2009 | 1 | 2 | 2 | 3 | 3 | 3 | 3 | 1 | 1 | 2 | 2 | 2 | 1 | 1 | 2 | 2 | 2 | 1 | 1 |
| **Cavallin** | 2021 | 1 | 2 | 2 | 3 | 3 | 3 | 3 | 1 | 1 | 2 | 2 | 2 | 1 | 1 | 2 | 2 | 2 | 1 | 2 |
| **Chan** | 2019 | 1 | 2 | 2 | 3 | 3 | 3 | 3 | 1 | 1 | 2 | 2 | 1 | 1 | 1 | 1 | 1 | 1 | 1 | 2 |
| **Clark** | 2022 | 1 | 2 | 2 | 3 | 3 | 3 | 3 | 1 | 1 | 2 | 2 | 2 | 1 | 1 | 2 | 2 | 2 | 2 | 2 |
| **Cordero** | 2013 | 2 | 2 | 2 | 3 | 3 | 3 | 3 | 1 | 2 | 2 | 2 | 2 | 2 | 2 | 2 | 2 | 2 | 1 | 2 |
| **Cordero** | 2014 | 2 | 2 | 2 | 3 | 3 | 3 | 3 | 1 | 2 | 2 | 2 | 2 | 2 | 2 | 2 | 2 | 2 | 1 | 2 |
| **Cronin** | 2001 | 1 | 2 | 2 | 3 | 3 | 3 | 3 | 1 | 1 | 2 | 2 | 2 | 1 | 1 | 1 | 2 | 1 | 1 | 2 |
| **Curran** | 2014 | 2 | 2 | 2 | 3 | 3 | 3 | 3 | 1 | 1 | 2 | 2 | 1 | 2 | 1 | 1 | 1 | 1 | 1 | 1 |
| **Cusack** | 2012 | 2 | 2 | 2 | 1 | 1 | 2 | 2 | 3 | 3 | 3 | 3 | 2 | 1 | 1 | 1 | 1 | 1 | 1 | 2 |
| **Das** | 2018 | 1 | 2 | 2 | 3 | 3 | 3 | 3 | 1 | 1 | 2 | 2 | 2 | 1 | 1 | 1 | 2 | 2 | 1 | 1 |
| **De Bernardo** | 2016 | 1 | 2 | 2 | 3 | 3 | 3 | 3 | 1 | 1 | 2 | 2 | 1 | 1 | 1 | 2 | 2 | 2 | 1 | 2 |
| **Delaney** | 2022 | 1 | 2 | 2 | 3 | 3 | 3 | 3 | 1 | 2 | 2 | 2 | 2 | 1 | 2 | 1 | 2 | 2 | 1 | 1 |
| **Ding** | 2021 | 2 | 2 | 2 | 3 | 3 | 3 | 3 | 1 | 1 | 2 | 2 | 2 | 2 | 1 | 1 | 1 | 1 | 1 | 1 |
| **Farhadi** | 2023 | 2 | 2 | 2 | 3 | 3 | 3 | 3 | 1 | 1 | 2 | 2 | 1 | 1 | 1 | 1 | 1 | 1 | 1 | 1 |
| **Ford** | 2023 | 1 | 1 | 2 | 3 | 3 | 3 | 3 | 2 | 2 | 2 | 2 | 2 | 1 | 2 | 1 | 1 | 1 | 1 | 1 |
| **Gamboa** | 2018 | 1 | 2 | 2 | 3 | 3 | 3 | 3 | 1 | 1 | 2 | 2 | 2 | 1 | 1 | 2 | 2 | 2 | 1 | 2 |
| **Gelbart** | 2010 | 1 | 2 | 2 | 3 | 3 | 3 | 3 | 1 | 1 | 2 | 1 | 1 | 1 | 1 | 2 | 2 | 1 | 1 | 2 |
| **Ghoman** | 2020 | 2 | 2 | 2 | 3 | 3 | 3 | 3 | 1 | 2 | 2 | 2 | 1 | 1 | 1 | 1 | 1 | 1 | 1 | 1 |
| **Greer** | 2023 | 1 | 2 | 2 | 3 | 3 | 3 | 3 | 1 | 1 | 1 | 1 | 1 | 1 | 2 | 1 | 1 | 1 | 1 | 1 |
| **Gross** | 2020 | 2 | 2 | 2 | 3 | 3 | 3 | 3 | 1 | 2 | 2 | 2 | 1 | 2 | 1 | 2 | 1 | 1 | 1 | 2 |
| **Holm-Hansen** | 2022 | 1 | 2 | 2 | 3 | 3 | 3 | 3 | 1 | 1 | 2 | 2 | 2 | 2 | 1 | 2 | 2 | 2 | 1 | 2 |
| **Horiuchi** | 2021 | 1 | 2 | 2 | 3 | 3 | 3 | 3 | 1 | 1 | 2 | 2 | 2 | 1 | 2 | 1 | 1 | 2 | 2 | 2 |
| **Hundscheid** | 2021 | 1 | 1 | 2 | 1 | 1 | 2 | 2 | 3 | 3 | 3 | 3 | 1 | 1 | 1 | 1 | 1 | 1 | 1 | 2 |
| **Johnson** | 2020 | 2 | 2 | 2 | 3 | 3 | 3 | 3 | 1 | 2 | 2 | 2 | 2 | 2 | 2 | 2 | 1 | 1 | 1 | 1 |
| **Kamath-Rayne** | 2019 | 2 | 2 | 1 | 3 | 3 | 3 | 3 | 1 | 1 | 2 | 2 | 2 | 1 | 1 | 1 | 1 | 2 | 1 | 1 |
| **Kamau** | 2022 | 2 | 2 | 2 | 3 | 3 | 3 | 3 | 1 | 1 | 2 | 2 | 2 | 2 | 1 | 2 | 2 | 2 | 2 | 2 |
| **Kane** | 2019 | 2 | 2 | 2 | 3 | 3 | 3 | 3 | 1 | 2 | 2 | 2 | 2 | 2 | 1 | 1 | 1 | 1 | 1 | 1 |
| **Kannan Loganathan** | 2024 | 2 | 2 | 1 | 1 | 1 | 2 | 2 | 3 | 3 | 3 | 3 | 1 | 1 | 1 | 1 | 2 | 2 | 2 | 2 |
| **Khriesat** | 2017 | 2 | 2 | 2 | 3 | 3 | 3 | 3 | 2 | 2 | 2 | 2 | 2 | 2 | 2 | 2 | 2 | 2 | 2 | 2 |
| **Kim** | 2013 | 1 | 2 | 2 | 3 | 3 | 3 | 3 | 1 | 1 | 2 | 2 | 2 | 1 | 2 | 1 | 2 | 2 | 1 | 1 |
| **Lizotte** | 2017 | 2 | 2 | 1 | 3 | 3 | 3 | 3 | 1 | 1 | 2 | 2 | 1 | 1 | 1 | 1 | 1 | 1 | 1 | 1 |
| **Lockyer** | 2006 | 2 | 2 | 2 | 3 | 3 | 3 | 3 | 1 | 1 | 1 | 2 | 2 | 1 | 2 | 1 | 1 | 1 | 1 | 2 |
| **Magee** | 2018 | 1 | 2 | 2 | 3 | 3 | 3 | 3 | 1 | 1 | 1 | 2 | 1 | 1 | 1 | 1 | 1 | 1 | 2 | 2 |
| **Maya-Enero** | 2018 | 1 | 2 | 1 | 3 | 3 | 3 | 3 | 1 | 1 | 2 | 1 | 1 | 2 | 1 | 2 | 1 | 1 | 1 | 2 |
| **McCaw** | 2023 | 1 | 2 | 2 | 3 | 3 | 3 | 3 | 1 | 1 | 2 | 2 | 2 | 1 | 1 | 1 | 2 | 2 | 1 | 1 |
| **Meggiolaro** | 2025 | 2 | 2 | 2 | 3 | 3 | 3 | 3 | 1 | 2 | 2 | 1 | 1 | 2 | 2 | 1 | 1 | 1 | 1 | 1 |
| **Mersha** | 2019 | 1 | 2 | 2 | 3 | 3 | 3 | 3 | 1 | 1 | 2 | 2 | 2 | 1 | 1 | 1 | 2 | 2 | 1 | 1 |
| **Mileder** | 2023 | 2 | 2 | 2 | 1 | 2 | 2 | 2 | 3 | 3 | 3 | 3 | 1 | 2 | 2 | 2 | 2 | 2 | 2 | 2 |
| **Nadler** | 2011 | 2 | 2 | 2 | 3 | 3 | 3 | 3 | 2 | 2 | 2 | 2 | 1 | 2 | 2 | 2 | 2 | 1 | 2 | 2 |
| **Nickerson** | 2019 | 1 | 2 | 2 | 3 | 3 | 3 | 3 | 1 | 1 | 2 | 2 | 2 | 1 | 2 | 2 | 1 | 1 | 2 | 2 |
| **Nvonako** | 2022 | 2 | 2 | 2 | 3 | 3 | 3 | 3 | 1 | 1 | 2 | 2 | 2 | 2 | 1 | 2 | 2 | 2 | 2 | 2 |
| **Paliatsiou** | 2021 | 1 | 1 | 2 | 1 | 1 | 2 | 2 | 3 | 3 | 3 | 3 | 2 | 2 | 2 | 2 | 1 | 1 | 2 | 2 |
| **Reisman** | 2015 | 2 | 2 | 2 | 3 | 3 | 3 | 3 | 1 | 1 | 2 | 2 | 2 | 1 | 2 | 1 | 2 | 2 | 1 | 1 |
| **Roitsch** | 2020 | 2 | 2 | 2 | 3 | 3 | 3 | 3 | 1 | 1 | 2 | 2 | 1 | 1 | 1 | 1 | 1 | 1 | 1 | 1 |
| **Rovamo** | 2013 | 1 | 2 | 2 | 3 | 3 | 3 | 3 | 1 | 1 | 2 | 2 | 1 | 1 | 1 | 2 | 2 | 2 | 1 | 2 |
| **Rovamo** | 2011 | 1 | 2 | 2 | 3 | 3 | 3 | 3 | 1 | 1 | 2 | 2 | 1 | 1 | 1 | 2 | 1 | 1 | 1 | 2 |
| **Sarvan** | 2022 | 1 | 2 | 2 | 2 | 2 | 2 | 2 | 1 | 1 | 2 | 2 | 2 | 2 | 1 | 2 | 2 | 2 | 2 | 2 |
| **Sawyer** | 2011 | 2 | 2 | 2 | 3 | 3 | 3 | 3 | 1 | 1 | 2 | 2 | 2 | 1 | 1 | 2 | 2 | 2 | 1 | 2 |
| **Shikuku** | 2017 | 2 | 2 | 2 | 3 | 3 | 3 | 3 | 1 | 1 | 2 | 2 | 2 | 1 | 1 | 2 | 2 | 2 | 1 | 2 |
| **Sinha** | 2024 | 1 | 2 | 2 | 3 | 3 | 3 | 3 | 1 | 1 | 2 | 2 | 2 | 1 | 1 | 1 | 2 | 2 | 1 | 1 |
| **Sintayehu** | 2020 | 1 | 2 | 2 | 3 | 3 | 3 | 3 | 1 | 1 | 2 | 2 | 2 | 1 | 2 | 1 | 2 | 2 | 1 | 2 |
| **Skâre** | 2018 | 1 | 2 | 2 | 3 | 3 | 3 | 3 | 1 | 1 | 2 | 2 | 2 | 1 | 1 | 2 | 1 | 1 | 1 | 2 |
| **Sloane** | 2021 | 1 | 2 | 1 | 3 | 3 | 3 | 3 | 1 | 1 | 2 | 2 | 1 | 1 | 1 | 2 | 1 | 1 | 1 | 2 |
| **Szyld** | 2021 | 2 | 2 | 2 | 3 | 3 | 3 | 3 | 1 | 1 | 2 | 2 | 2 | 1 | 1 | 1 | 1 | 1 | 1 | 1 |
| **Tosif** | 2020 | 1 | 2 | 2 | 3 | 3 | 3 | 3 | 1 | 1 | 2 | 2 | 2 | 1 | 2 | 1 | 1 | 1 | 1 | 1 |
| **Trevisanuto** | 2015 | 1 | 2 | 2 | 3 | 3 | 3 | 3 | 1 | 1 | 2 | 2 | 2 | 1 | 1 | 2 | 2 | 2 | 2 | 2 |
| **Tsang** | 2022 | 2 | 2 | 2 | 1 | 1 | 2 | 2 | 3 | 3 | 3 | 3 | 2 | 1 | 1 | 2 | 2 | 2 | 2 | 2 |
| **Vail** | 2018 | 2 | 2 | 2 | 1 | 2 | 2 | 2 | 1 | 1 | 2 | 2 | 2 | 1 | 2 | 2 | 2 | 2 | 2 | 2 |
| **Vail** | 2017 | 2 | 2 | 2 | 3 | 3 | 3 | 3 | 1 | 2 | 2 | 2 | 2 | 2 | 2 | 2 | 2 | 2 | 2 | 2 |
| **Van der Heide** | 2006 | 1 | 2 | 2 | 3 | 3 | 3 | 3 | 1 | 1 | 2 | 2 | 1 | 1 | 1 | 2 | 1 | 1 | 1 | 2 |
| **Woods** | 2015 | 1 | 1 | 2 | 3 | 3 | 3 | 3 | 1 | 1 | 2 | 2 | 2 | 1 | 1 | 2 | 2 | 2 | 2 | 2 |
| **Xu** | 2023 | 2 | 2 | 2 | 3 | 3 | 3 | 3 | 1 | 2 | 2 | 2 | 1 | 1 | 1 | 1 | 1 | 1 | 1 | 1 |
| **Yamada** | 2016 | 1 | 2 | 1 | 3 | 3 | 3 | 3 | 1 | 1 | 1 | 1 | 1 | 1 | 1 | 1 | 1 | 1 | 1 | 1 |
| **Yamada** | 2015 | 1 | 2 | 1 | 3 | 3 | 3 | 3 | 1 | 1 | 1 | 1 | 1 | 1 | 1 | 1 | 1 | 1 | 1 | 1 |
| **Yaylaci** | 2021 | 2 | 2 | 2 | 3 | 3 | 3 | 3 | 1 | 1 | 2 | 2 | 1 | 1 | 1 | 2 | 2 | 2 | 1 | 2 |
| **Yeo** | 2020 | 1 | 2 | 2 | 3 | 3 | 3 | 3 | 1 | 1 | 2 | 2 | 1 | 1 | 2 | 1 | 1 | 1 | 1 | 1 |
| **Yoosoof** | 2022 | 1 | 1 | 2 | 3 | 3 | 3 | 3 | 1 | 2 | 2 | 2 | 1 | 1 | 2 | 2 | 2 | 2 | 2 | 2 |

Part 3: Airway management 2 and circulation

|  | | **ERC** | **NRP** | **Reassess: check effect** | | **ERC: Chest is moving 🡪 continue ventilation breaths NRP: directly proceed to chest compressions** | | **Reassess: check effect** | | **Heart rate not detectable or <60/min after 30 seconds of ventilation: indication chest compressions (CC)** | | | | | | |  |  |  |  |  |
| --- | --- | --- | --- | --- | --- | --- | --- | --- | --- | --- | --- | --- | --- | --- | --- | --- | --- | --- | --- | --- | --- |
| **First author** | **Year of publication** | **Repeat 5 insufflation breaths** | **Continue PPV** | **Assess: chest movements** | **Assess: heart rate, breathing** | **Start ventilation breaths for 30 seconds** | **Frequency 30-60 breaths / minute** | **Assess: chest movements** | **Assess: heart rate, breathing** | **Start chest compressions and continue ventilation** | **Increase oxygen to 100%** | **Uses correct algorithm of CPR 3:1** | **Two-thumb technique** | **Frequency of CC +- 100-120/min** | **Depth of CC +- 1/3 of the AP diameter of the chest** | **Full chest recoil** | **Reassess hear rate every 30 seconds** | **Consider intubation if not already done or laryngeal mask if not possible** | **Stop CPR for heart rate reassessment** | **Assess: CC still needed?** | **Reassess breathing after chest compression was ceased** |
|  |  | **1 = Yes 2 = No 3 = Not applicable** | **1 = Yes 2 = No 3 = Not applicable** | **1 = Yes 2 = No** | **1 = Yes 2 = No** | **1 = Yes 2 = No 3 = Not applicable** | **1 = Yes 2 = No 3 = Not applicable** | **1 = Yes 2 = No 3 = Not applicable** | **1 = Yes 2 = No 3 = Not applicable** | **1 = Yes 2 = No** | **1 = Yes 2 = No** | **1 = Yes 2 = No** | **1 = Yes 2 = No** | **1 = Yes 2 = No** | **1 = Yes 2 = No** | **1 = Yes 2 = No** | **1 = Yes 2 = No** | **1 = Yes 2 = No** | **1 = Yes 2 = No** | **1 = Yes 2 = No** | **1 = Yes 2 = No** |
| **ERC 2021** | 2021 | 1 | 3 | 1 | 1 | 1 | 2 | 1 | 1 | 1 | 1 | 1 | 2 | 2 | 2 | 2 | 2 | 2 | 2 | 2 | 2 |
| **HBB 1st edition** | 2010 | 3 | 1 | 2 | 2 | 3 | 3 | 3 | 3 | 2 | 2 | 2 | 2 | 2 | 2 | 2 | 2 | 2 | 2 | 2 | 2 |
| **HBB 2nd edition** | 2016 | 3 | 1 | 1 | 1 | 3 | 3 | 3 | 3 | 2 | 2 | 2 | 2 | 2 | 2 | 2 | 2 | 2 | 2 | 2 | 2 |
| **Athanasopoulou** | 2024 | 3 | 2 | 2 | 2 | 3 | 3 | 3 | 3 | 1 | 2 | 2 | 2 | 2 | 2 | 2 | 2 | 2 | 2 | 2 | 2 |
| **Barbato** | 2020 | 3 | 2 | 2 | 2 | 3 | 3 | 3 | 3 | 2 | 2 | 2 | 2 | 2 | 2 | 2 | 2 | 2 | 2 | 2 | 2 |
| **Barry** | 2012 | 3 | 2 | 2 | 2 | 3 | 3 | 3 | 3 | 1 | 2 | 2 | 2 | 2 | 2 | 2 | 2 | 2 | 2 | 2 | 2 |
| **Bender** | 2014 | 3 | 1 | 1 | 1 | 3 | 3 | 3 | 3 | 1 | 2 | 1 | 1 | 1 | 1 | 2 | 1 | 1 | 2 | 1 | 1 |
| **Bibl** | 2023 | 1 | 3 | 1 | 1 | 1 | 2 | 1 | 1 | 1 | 2 | 1 | 2 | 2 | 2 | 2 | 1 | 2 | 1 | 1 | 1 |
| **Billner-Garcia** | 2022 | 3 | 2 | 2 | 2 | 3 | 3 | 3 | 3 | 2 | 2 | 2 | 2 | 2 | 2 | 2 | 2 | 2 | 2 | 2 | 2 |
| **Binkhorst** | 2020 | 2 | 3 | 2 | 2 | 2 | 2 | 2 | 2 | 1 | 1 | 2 | 2 | 1 | 2 | 2 | 2 | 2 | 2 | 2 | 2 |
| **Brathwaite** | 2020 | 3 | 2 | 2 | 2 | 3 | 3 | 3 | 3 | 1 | 2 | 1 | 1 | 2 | 2 | 2 | 1 | 2 | 2 | 2 | 2 |
| **Brogaard** | 2022 | 2 | 3 | 2 | 1 | 1 | 1 | 2 | 1 | 1 | 2 | 1 | 2 | 2 | 2 | 2 | 1 | 2 | 2 | 2 | 2 |
| **Caldelari** | 2019 | 3 | 1 | 2 | 1 | 3 | 3 | 3 | 3 | 1 | 1 | 1 | 1 | 1 | 1 | 1 | 1 | 2 | 2 | 2 | 2 |
| **Campbell** | 2009 | 3 | 2 | 2 | 2 | 3 | 3 | 3 | 3 | 1 | 2 | 2 | 2 | 2 | 2 | 2 | 2 | 2 | 2 | 2 | 2 |
| **Carbine** | 2000 | 3 | 2 | 2 | 2 | 3 | 3 | 3 | 3 | 1 | 2 | 1 | 1 | 1 | 1 | 2 | 1 | 1 | 2 | 2 | 2 |
| **Carlo** | 2009 | 3 | 1 | 1 | 1 | 3 | 3 | 3 | 3 | 1 | 2 | 1 | 1 | 2 | 1 | 2 | 2 | 2 | 2 | 2 | 2 |
| **Cavallin** | 2021 | 3 | 2 | 2 | 2 | 3 | 3 | 3 | 3 | 1 | 2 | 1 | 1 | 1 | 1 | 2 | 1 | 2 | 2 | 2 | 2 |
| **Chan** | 2019 | 3 | 1 | 2 | 1 | 3 | 3 | 3 | 3 | 1 | 1 | 1 | 1 | 2 | 1 | 2 | 1 | 2 | 2 | 2 | 2 |
| **Clark** | 2022 | 3 | 1 | 2 | 2 | 3 | 3 | 3 | 3 | 2 | 2 | 2 | 2 | 2 | 2 | 2 | 2 | 2 | 2 | 2 | 2 |
| **Cordero** | 2013 | 3 | 2 | 2 | 2 | 3 | 3 | 3 | 3 | 1 | 2 | 1 | 1 | 2 | 2 | 2 | 1 | 1 | 2 | 2 | 2 |
| **Cordero** | 2014 | 3 | 2 | 2 | 2 | 3 | 3 | 3 | 3 | 1 | 2 | 1 | 1 | 2 | 2 | 2 | 1 | 1 | 2 | 2 | 2 |
| **Cronin** | 2001 | 3 | 1 | 2 | 2 | 3 | 3 | 3 | 3 | 1 | 2 | 1 | 2 | 2 | 2 | 2 | 1 | 1 | 1 | 1 | 1 |
| **Curran** | 2014 | 3 | 1 | 1 | 1 | 3 | 3 | 3 | 3 | 1 | 1 | 1 | 1 | 1 | 1 | 2 | 2 | 2 | 2 | 2 | 2 |
| **Cusack** | 2012 | 1 | 3 | 1 | 1 | 1 | 2 | 2 | 2 | 1 | 2 | 2 | 2 | 2 | 2 | 2 | 2 | 2 | 2 | 2 | 2 |
| **Das** | 2018 | 3 | 1 | 1 | 1 | 3 | 3 | 3 | 2 | 2 | 2 | 2 | 2 | 2 | 2 | 2 | 2 | 2 | 2 | 2 | 2 |
| **De Bernardo** | 2016 | 2 | 2 | 2 | 2 | 2 | 2 | 2 | 2 | 2 | 2 | 2 | 2 | 2 | 2 | 2 | 2 | 2 | 2 | 2 | 2 |
| **Delaney** | 2022 | 3 | 1 | 1 | 1 | 3 | 3 | 3 | 3 | 2 | 2 | 2 | 2 | 2 | 2 | 2 | 2 | 2 | 2 | 2 | 2 |
| **Ding** | 2021 | 3 | 1 | 2 | 1 | 3 | 3 | 3 | 3 | 1 | 2 | 1 | 1 | 1 | 2 | 2 | 2 | 2 | 2 | 2 | 2 |
| **Farhadi** | 2023 | 3 | 1 | 2 | 1 | 3 | 3 | 3 | 3 | 1 | 1 | 1 | 1 | 2 | 1 | 1 | 2 | 1 | 2 | 2 | 2 |
| **Ford** | 2023 | 3 | 2 | 2 | 1 | 3 | 3 | 3 | 3 | 2 | 2 | 2 | 2 | 2 | 2 | 2 | 2 | 2 | 2 | 2 | 2 |
| **Gamboa** | 2018 | 3 | 2 | 2 | 2 | 3 | 3 | 3 | 3 | 1 | 2 | 1 | 1 | 1 | 2 | 2 | 1 | 1 | 2 | 1 | 2 |
| **Gelbart** | 2010 | 3 | 1 | 1 | 1 | 3 | 3 | 1 | 1 | 1 | 2 | 1 | 1 | 1 | 1 | 2 | 1 | 2 | 2 | 2 | 2 |
| **Ghoman** | 2020 | 3 | 1 | 1 | 1 | 3 | 3 | 3 | 3 | 1 | 1 | 2 | 2 | 2 | 2 | 2 | 1 | 2 | 2 | 1 | 1 |
| **Greer** | 2023 | 3 | 1 | 2 | 1 | 3 | 3 | 3 | 3 | 1 | 2 | 2 | 2 | 2 | 2 | 2 | 1 | 1 | 2 | 1 | 2 |
| **Gross** | 2020 | 3 | 2 | 2 | 1 | 3 | 3 | 3 | 3 | 1 | 1 | 1 | 2 | 2 | 2 | 2 | 2 | 2 | 2 | 2 | 2 |
| **Holm-Hansen** | 2022 | 3 | 2 | 2 | 2 | 3 | 3 | 3 | 3 | 2 | 2 | 2 | 2 | 2 | 2 | 2 | 2 | 2 | 2 | 2 | 2 |
| **Horiuchi** | 2021 | 3 | 1 | 1 | 1 | 3 | 3 | 3 | 3 | 2 | 2 | 2 | 2 | 2 | 2 | 2 | 2 | 2 | 2 | 2 | 2 |
| **Hundscheid** | 2021 | 1 | 3 | 1 | 1 | 1 | 2 | 1 | 1 | 1 | 1 | 1 | 2 | 2 | 2 | 2 | 1 | 2 | 2 | 2 | 2 |
| **Johnson** | 2020 | 3 | 2 | 2 | 2 | 3 | 3 | 3 | 3 | 1 | 2 | 2 | 2 | 2 | 2 | 2 | 2 | 2 | 2 | 2 | 2 |
| **Kamath-Rayne** | 2019 | 3 | 1 | 1 | 1 | 3 | 3 | 3 | 3 | 2 | 2 | 2 | 2 | 2 | 2 | 2 | 2 | 2 | 2 | 2 | 2 |
| **Kamau** | 2022 | 3 | 2 | 2 | 2 | 3 | 3 | 3 | 3 | 2 | 2 | 2 | 2 | 2 | 2 | 2 | 2 | 2 | 2 | 2 | 2 |
| **Kane** | 2019 | 3 | 2 | 2 | 2 | 3 | 3 | 3 | 3 | 2 | 2 | 2 | 2 | 2 | 2 | 2 | 2 | 2 | 2 | 2 | 2 |
| **Kannan Loganathan** | 2024 | 1 | 3 | 1 | 1 | 1 | 1 | 1 | 1 | 1 | 1 | 1 | 1 | 2 | 1 | 1 | 2 | 1 | 2 | 2 | 2 |
| **Khriesat** | 2017 | 3 | 2 | 2 | 2 | 3 | 3 | 3 | 3 | 2 | 2 | 2 | 2 | 2 | 2 | 2 | 2 | 2 | 2 | 2 | 2 |
| **Kim** | 2013 | 3 | 1 | 1 | 1 | 3 | 3 | 3 | 3 | 2 | 2 | 2 | 2 | 2 | 2 | 2 | 2 | 2 | 2 | 2 | 2 |
| **Lizotte** | 2017 | 3 | 1 | 2 | 1 | 3 | 3 | 3 | 3 | 1 | 1 | 1 | 1 | 1 | 1 | 2 | 2 | 1 | 2 | 2 | 2 |
| **Lockyer** | 2006 | 3 | 1 | 2 | 1 | 3 | 3 | 3 | 3 | 1 | 2 | 1 | 1 | 1 | 1 | 2 | 2 | 1 | 2 | 1 | 1 |
| **Magee** | 2018 | 3 | 1 | 1 | 1 | 3 | 3 | 3 | 3 | 1 | 1 | 1 | 1 | 2 | 2 | 2 | 2 | 1 | 2 | 1 | 1 |
| **Maya-Enero** | 2018 | 3 | 2 | 2 | 2 | 3 | 3 | 3 | 3 | 1 | 2 | 1 | 1 | 1 | 1 | 2 | 1 | 2 | 2 | 2 | 2 |
| **McCaw** | 2023 | 3 | 1 | 1 | 1 | 3 | 3 | 3 | 3 | 2 | 2 | 2 | 2 | 2 | 2 | 2 | 2 | 2 | 2 | 2 | 2 |
| **Meggiolaro** | 2025 | 3 | 1 | 2 | 2 | 3 | 3 | 3 | 3 | 1 | 1 | 2 | 2 | 2 | 2 | 2 | 2 | 2 | 2 | 2 | 2 |
| **Mersha** | 2019 | 3 | 1 | 2 | 1 | 3 | 3 | 3 | 3 | 2 | 2 | 2 | 2 | 2 | 2 | 2 | 2 | 2 | 2 | 2 | 2 |
| **Mileder** | 2023 | 2 | 3 | 3 | 3 | 2 | 2 | 2 | 2 | 2 | 2 | 2 | 2 | 2 | 2 | 2 | 2 | 2 | 2 | 2 | 2 |
| **Nadler** | 2011 | 3 | 2 | 2 | 2 | 3 | 3 | 3 | 3 | 2 | 2 | 2 | 2 | 2 | 2 | 2 | 2 | 2 | 2 | 2 | 2 |
| **Nickerson** | 2019 | 3 | 2 | 2 | 2 | 3 | 3 | 3 | 3 | 1 | 2 | 2 | 2 | 1 | 1 | 2 | 2 | 2 | 2 | 2 | 2 |
| **Nvonako** | 2022 | 3 | 2 | 2 | 2 | 3 | 3 | 3 | 3 | 2 | 2 | 2 | 2 | 2 | 2 | 2 | 2 | 2 | 2 | 2 | 2 |
| **Paliatsiou** | 2021 | 2 | 3 | 2 | 2 | 1 | 1 | 2 | 2 | 1 | 2 | 1 | 2 | 1 | 1 | 2 | 2 | 2 | 2 | 2 | 2 |
| **Reisman** | 2015 | 3 | 1 | 2 | 1 | 3 | 3 | 3 | 3 | 2 | 2 | 2 | 2 | 2 | 2 | 2 | 2 | 2 | 2 | 2 | 2 |
| **Roitsch** | 2020 | 3 | 1 | 1 | 1 | 3 | 3 | 3 | 3 | 1 | 1 | 1 | 1 | 2 | 1 | 1 | 2 | 2 | 2 | 1 | 2 |
| **Rovamo** | 2013 | 3 | 2 | 2 | 2 | 3 | 3 | 3 | 3 | 2 | 2 | 2 | 2 | 2 | 2 | 2 | 2 | 2 | 2 | 2 | 2 |
| **Rovamo** | 2011 | 3 | 2 | 2 | 2 | 3 | 3 | 3 | 3 | 1 | 2 | 2 | 2 | 2 | 2 | 2 | 2 | 1 | 2 | 2 | 2 |
| **Sarvan** | 2022 | 3 | 2 | 2 | 2 | 3 | 3 | 3 | 3 | 1 | 2 | 2 | 1 | 1 | 1 | 2 | 1 | 2 | 2 | 1 | 2 |
| **Sawyer** | 2011 | 3 | 2 | 2 | 2 | 3 | 3 | 3 | 3 | 1 | 2 | 1 | 1 | 1 | 2 | 2 | 1 | 1 | 2 | 2 | 2 |
| **Shikuku** | 2017 | 3 | 2 | 2 | 2 | 3 | 3 | 3 | 3 | 2 | 2 | 2 | 2 | 2 | 2 | 2 | 2 | 2 | 2 | 2 | 2 |
| **Sinha** | 2024 | 3 | 1 | 1 | 1 | 3 | 3 | 3 | 3 | 2 | 2 | 2 | 2 | 2 | 2 | 2 | 2 | 2 | 2 | 2 | 2 |
| **Sintayehu** | 2020 | 3 | 1 | 1 | 1 | 3 | 3 | 3 | 3 | 2 | 2 | 2 | 2 | 2 | 2 | 2 | 2 | 2 | 2 | 2 | 2 |
| **Skâre** | 2018 | 3 | 2 | 2 | 2 | 3 | 3 | 3 | 3 | 1 | 2 | 2 | 2 | 2 | 2 | 2 | 2 | 2 | 2 | 2 | 2 |
| **Sloane** | 2021 | 3 | 2 | 2 | 2 | 3 | 3 | 3 | 3 | 1 | 2 | 1 | 1 | 1 | 1 | 2 | 1 | 1 | 2 | 2 | 2 |
| **Szyld** | 2021 | 3 | 1 | 1 | 2 | 3 | 3 | 3 | 3 | 2 | 2 | 2 | 2 | 2 | 2 | 2 | 2 | 2 | 2 | 2 | 2 |
| **Tosif** | 2020 | 3 | 2 | 2 | 2 | 3 | 3 | 3 | 3 | 2 | 2 | 2 | 2 | 2 | 2 | 2 | 2 | 2 | 2 | 2 | 2 |
| **Trevisanuto** | 2015 | 3 | 2 | 2 | 2 | 3 | 3 | 3 | 3 | 1 | 2 | 1 | 1 | 1 | 1 | 2 | 1 | 2 | 2 | 2 | 2 |
| **Tsang** | 2022 | 2 | 3 | 2 | 2 | 1 | 1 | 1 | 1 | 1 | 1 | 1 | 2 | 2 | 2 | 2 | 1 | 2 | 2 | 1 | 1 |
| **Vail** | 2018 | 3 | 2 | 2 | 2 | 3 | 3 | 3 | 3 | 2 | 2 | 2 | 2 | 2 | 2 | 2 | 2 | 2 | 2 | 2 | 2 |
| **Vail** | 2017 | 2 | 2 | 2 | 2 | 2 | 2 | 2 | 2 | 2 | 2 | 2 | 2 | 2 | 2 | 2 | 2 | 2 | 2 | 2 | 2 |
| **Van der Heide** | 2006 | 3 | 2 | 2 | 1 | 3 | 3 | 3 | 3 | 1 | 2 | 1 | 1 | 1 | 1 | 2 | 1 | 2 | 2 | 2 | 2 |
| **Woods** | 2015 | 3 | 1 | 2 | 2 | 3 | 3 | 3 | 3 | 2 | 2 | 2 | 2 | 2 | 2 | 2 | 2 | 2 | 2 | 2 | 2 |
| **Xu** | 2023 | 3 | 1 | 2 | 1 | 3 | 3 | 3 | 3 | 1 | 1 | 1 | 1 | 1 | 1 | 1 | 2 | 1 | 2 | 2 | 2 |
| **Yamada** | 2016 | 3 | 1 | 1 | 2 | 3 | 3 | 3 | 3 | 1 | 1 | 1 | 1 | 2 | 1 | 2 | 1 | 1 | 2 | 2 | 2 |
| **Yamada** | 2015 | 3 | 1 | 1 | 1 | 3 | 3 | 3 | 3 | 1 | 1 | 1 | 1 | 2 | 1 | 2 | 1 | 1 | 2 | 2 | 2 |
| **Yaylaci** | 2021 | 3 | 1 | 1 | 1 | 3 | 3 | 3 | 3 | 1 | 1 | 1 | 1 | 2 | 2 | 2 | 1 | 2 | 2 | 2 | 2 |
| **Yeo** | 2020 | 3 | 1 | 2 | 1 | 3 | 3 | 3 | 3 | 1 | 1 | 1 | 1 | 2 | 1 | 2 | 1 | 2 | 2 | 2 | 2 |
| **Yoosoof** | 2022 | 3 | 2 | 2 | 2 | 3 | 3 | 3 | 3 | 1 | 2 | 1 | 1 | 2 | 1 | 2 | 1 | 1 | 2 | 2 | 2 |

Part 4: Final steps and debriefing

|  | | **Heart rate remains not detectable or <60/min** | | | | | | **Debriefing** | | | **During the whole algorithm** | | |
| --- | --- | --- | --- | --- | --- | --- | --- | --- | --- | --- | --- | --- | --- |
| **First author** | **Year of publication** | **Vascular access: umbilical vein catheterization. If not possible: intra-ossal access.** | **Epinephrine 10-30 mcg/kg** | **Repeat epinephrine every 3-5 minutes** | **Consider bolus of glucose** | **Consider other factors: pneumothorax, hypovoleamia, congenital abnormalities** | **Decision on discontinuation of resuscitation** | **Update parents** | **Debrief with team** | **Ensure adequate documentation** | **Call for help when needed** | **Titrate FiO2 to achieve target saturations** | **Titrate inspiration pressure** |
|  |  | **1 = Yes 2 = No** | **1 = Yes 2 = No** | **1 = Yes 2 = No** | **1 = Yes 2 = No** | **1 = Yes 2 = No** | **1 = Yes 2 = No** | **1 = Yes 2 = No** | **1 = Yes 2 = No** | **1 = Yes 2 = No** | **1 = Yes 2 = No** | **1 = Yes 2 = No** | **1 = Yes 2 = No** |
| **ERC 2021** | 2021 | 2 | 2 | 2 | 2 | 2 | 2 | 2 | 2 | 2 | 1 | 2 | 2 |
| **HBB 1st edition** | 2010 | 2 | 2 | 2 | 2 | 2 | 2 | 1 | 2 | 2 | 1 | 2 | 2 |
| **HBB 2nd edition** | 2016 | 2 | 2 | 2 | 2 | 2 | 2 | 1 | 2 | 2 | 1 | 2 | 2 |
| **Athanasopoulou** | 2024 | 2 | 2 | 2 | 1 | 2 | 2 | 2 | 2 | 2 | 2 | 2 | 2 |
| **Barbato** | 2020 | 2 | 2 | 2 | 2 | 2 | 2 | 2 | 2 | 2 | 2 | 2 | 2 |
| **Barry** | 2012 | 1 | 1 | 2 | 2 | 1 | 2 | 2 | 2 | 2 | 2 | 1 | 2 |
| **Bender** | 2014 | 1 | 1 | 1 | 2 | 1 | 2 | 1 | 2 | 2 | 1 | 2 | 2 |
| **Bibl** | 2023 | 1 | 2 | 2 | 2 | 2 | 2 | 2 | 2 | 2 | 1 | 2 | 2 |
| **Billner-Garcia** | 2022 | 2 | 2 | 2 | 2 | 2 | 2 | 2 | 2 | 2 | 1 | 2 | 2 |
| **Binkhorst** | 2020 | 2 | 1 | 2 | 2 | 2 | 2 | 2 | 2 | 2 | 2 | 2 | 2 |
| **Brathwaite** | 2020 | 2 | 2 | 2 | 2 | 2 | 2 | 2 | 2 | 2 | 2 | 2 | 2 |
| **Brogaard** | 2022 | 1 | 2 | 2 | 2 | 1 | 2 | 2 | 2 | 2 | 2 | 2 | 2 |
| **Caldelari** | 2019 | 2 | 2 | 2 | 2 | 2 | 2 | 2 | 2 | 2 | 1 | 2 | 2 |
| **Campbell** | 2009 | 1 | 1 | 2 | 2 | 1 | 2 | 2 | 2 | 2 | 1 | 2 | 2 |
| **Carbine** | 2000 | 1 | 1 | 2 | 2 | 1 | 2 | 2 | 2 | 2 | 2 | 2 | 2 |
| **Carlo** | 2009 | 2 | 2 | 2 | 2 | 2 | 2 | 2 | 2 | 2 | 1 | 2 | 2 |
| **Cavallin** | 2021 | 2 | 2 | 2 | 2 | 2 | 2 | 2 | 2 | 2 | 2 | 2 | 2 |
| **Chan** | 2019 | 2 | 2 | 2 | 2 | 2 | 2 | 2 | 2 | 2 | 1 | 2 | 2 |
| **Clark** | 2022 | 2 | 2 | 2 | 2 | 2 | 2 | 2 | 2 | 2 | 2 | 2 | 2 |
| **Cordero** | 2013 | 1 | 1 | 2 | 2 | 2 | 2 | 2 | 2 | 2 | 2 | 2 | 2 |
| **Cordero** | 2014 | 1 | 1 | 2 | 2 | 2 | 2 | 2 | 2 | 2 | 2 | 2 | 2 |
| **Cronin** | 2001 | 2 | 2 | 2 | 2 | 2 | 2 | 2 | 2 | 2 | 2 | 2 | 2 |
| **Curran** | 2014 | 2 | 2 | 2 | 2 | 2 | 2 | 2 | 2 | 2 | 1 | 1 | 2 |
| **Cusack** | 2012 | 1 | 1 | 2 | 2 | 1 | 2 | 2 | 2 | 2 | 1 | 2 | 2 |
| **Das** | 2018 | 2 | 2 | 2 | 2 | 2 | 2 | 2 | 2 | 2 | 2 | 2 | 2 |
| **De Bernardo** | 2016 | 2 | 2 | 2 | 2 | 2 | 2 | 2 | 2 | 2 | 2 | 1 | 2 |
| **Delaney** | 2022 | 2 | 2 | 2 | 2 | 2 | 2 | 2 | 2 | 2 | 2 | 1 | 2 |
| **Ding** | 2021 | 1 | 1 | 2 | 2 | 1 | 2 | 2 | 2 | 2 | 2 | 1 | 2 |
| **Farhadi** | 2023 | 1 | 1 | 2 | 2 | 1 | 2 | 2 | 2 | 2 | 2 | 1 | 2 |
| **Ford** | 2023 | 2 | 2 | 2 | 2 | 2 | 2 | 2 | 2 | 2 | 2 | 2 | 2 |
| **Gamboa** | 2018 | 1 | 1 | 2 | 2 | 1 | 2 | 2 | 2 | 2 | 1 | 2 | 2 |
| **Gelbart** | 2010 | 1 | 1 | 2 | 2 | 1 | 2 | 2 | 2 | 2 | 2 | 2 | 2 |
| **Ghoman** | 2020 | 2 | 2 | 2 | 2 | 2 | 2 | 1 | 2 | 2 | 1 | 2 | 2 |
| **Greer** | 2023 | 1 | 2 | 2 | 2 | 2 | 2 | 2 | 2 | 2 | 1 | 1 | 2 |
| **Gross** | 2020 | 2 | 1 | 2 | 2 | 2 | 2 | 2 | 2 | 2 | 2 | 2 | 2 |
| **Holm-Hansen** | 2022 | 2 | 2 | 2 | 2 | 2 | 2 | 2 | 2 | 2 | 2 | 2 | 2 |
| **Horiuchi** | 2021 | 2 | 2 | 2 | 2 | 2 | 2 | 1 | 2 | 2 | 1 | 2 | 2 |
| **Hundscheid** | 2021 | 2 | 2 | 2 | 2 | 2 | 2 | 2 | 2 | 2 | 1 | 2 | 2 |
| **Johnson** | 2020 | 1 | 1 | 2 | 2 | 1 | 2 | 2 | 2 | 2 | 2 | 2 | 2 |
| **Kamath-Rayne** | 2019 | 2 | 2 | 2 | 2 | 2 | 2 | 2 | 2 | 2 | 1 | 2 | 2 |
| **Kamau** | 2022 | 2 | 2 | 2 | 2 | 2 | 2 | 2 | 2 | 2 | 1 | 2 | 2 |
| **Kane** | 2019 | 2 | 2 | 2 | 2 | 2 | 2 | 2 | 2 | 2 | 1 | 2 | 2 |
| **Kannan Loganathan** | 2024 | 1 | 1 | 2 | 2 | 1 | 2 | 2 | 2 | 2 | 1 | 2 | 2 |
| **Khriesat** | 2017 | 2 | 2 | 2 | 2 | 2 | 2 | 2 | 2 | 2 | 2 | 2 | 2 |
| **Kim** | 2013 | 2 | 2 | 2 | 2 | 2 | 2 | 2 | 2 | 2 | 2 | 2 | 2 |
| **Lizotte** | 2017 | 1 | 1 | 2 | 2 | 1 | 2 | 2 | 2 | 2 | 1 | 1 | 2 |
| **Lockyer** | 2006 | 1 | 1 | 2 | 2 | 1 | 2 | 2 | 2 | 2 | 2 | 2 | 2 |
| **Magee** | 2018 | 1 | 1 | 2 | 2 | 2 | 1 | 2 | 2 | 2 | 1 | 1 | 2 |
| **Maya-Enero** | 2018 | 1 | 1 | 2 | 2 | 2 | 2 | 2 | 2 | 2 | 2 | 2 | 2 |
| **McCaw** | 2023 | 2 | 2 | 2 | 2 | 2 | 2 | 1 | 2 | 2 | 1 | 2 | 2 |
| **Meggiolaro** | 2025 | 2 | 1 | 2 | 2 | 1 | 2 | 2 | 2 | 2 | 1 | 1 | 2 |
| **Mersha** | 2019 | 2 | 2 | 2 | 2 | 2 | 2 | 1 | 2 | 2 | 1 | 2 | 2 |
| **Mileder** | 2023 | 2 | 2 | 2 | 2 | 2 | 2 | 2 | 2 | 2 | 2 | 2 | 2 |
| **Nadler** | 2011 | 2 | 2 | 2 | 2 | 2 | 2 | 2 | 2 | 2 | 2 | 2 | 2 |
| **Nickerson** | 2019 | 2 | 2 | 2 | 2 | 2 | 2 | 2 | 2 | 2 | 2 | 2 | 2 |
| **Nvonako** | 2022 | 2 | 2 | 2 | 2 | 2 | 2 | 2 | 2 | 2 | 2 | 2 | 2 |
| **Paliatsiou** | 2021 | 2 | 2 | 2 | 2 | 2 | 2 | 2 | 2 | 2 | 2 | 2 | 2 |
| **Reisman** | 2015 | 2 | 2 | 2 | 2 | 2 | 2 | 2 | 2 | 2 | 2 | 2 | 2 |
| **Roitsch** | 2020 | 1 | 1 | 1 | 2 | 1 | 2 | 2 | 2 | 2 | 1 | 1 | 2 |
| **Rovamo** | 2013 | 2 | 2 | 2 | 2 | 2 | 2 | 2 | 2 | 2 | 1 | 1 | 2 |
| **Rovamo** | 2011 | 2 | 1 | 2 | 2 | 1 | 2 | 2 | 2 | 1 | 2 | 1 | 2 |
| **Sarvan** | 2022 | 2 | 2 | 2 | 2 | 2 | 2 | 2 | 2 | 2 | 2 | 2 | 2 |
| **Sawyer** | 2011 | 1 | 1 | 2 | 2 | 1 | 2 | 2 | 2 | 2 | 2 | 2 | 2 |
| **Shikuku** | 2017 | 2 | 2 | 2 | 2 | 2 | 2 | 2 | 2 | 2 | 1 | 1 | 2 |
| **Sinha** | 2024 | 2 | 2 | 2 | 2 | 2 | 2 | 2 | 2 | 2 | 1 | 2 | 2 |
| **Sintayehu** | 2020 | 2 | 2 | 2 | 2 | 2 | 2 | 1 | 2 | 2 | 2 | 2 | 2 |
| **Skâre** | 2018 | 1 | 2 | 2 | 2 | 2 | 2 | 2 | 2 | 2 | 1 | 2 | 2 |
| **Sloane** | 2021 | 1 | 1 | 2 | 2 | 1 | 2 | 2 | 2 | 2 | 2 | 2 | 2 |
| **Szyld** | 2021 | 2 | 2 | 2 | 2 | 2 | 2 | 2 | 2 | 2 | 1 | 2 | 2 |
| **Tosif** | 2020 | 2 | 2 | 2 | 2 | 2 | 2 | 2 | 2 | 2 | 1 | 2 | 2 |
| **Trevisanuto** | 2015 | 2 | 2 | 2 | 2 | 2 | 2 | 2 | 2 | 2 | 2 | 2 | 2 |
| **Tsang** | 2022 | 1 | 1 | 2 | 2 | 1 | 2 | 2 | 2 | 2 | 2 | 2 | 2 |
| **Vail** | 2018 | 2 | 2 | 2 | 2 | 2 | 2 | 2 | 2 | 2 | 2 | 2 | 2 |
| **Vail** | 2017 | 2 | 2 | 2 | 2 | 2 | 2 | 2 | 2 | 2 | 2 | 2 | 2 |
| **Van der Heide** | 2006 | 1 | 1 | 2 | 2 | 1 | 2 | 2 | 2 | 2 | 2 | 2 | 2 |
| **Woods** | 2015 | 2 | 2 | 2 | 2 | 2 | 2 | 2 | 2 | 2 | 2 | 2 | 2 |
| **Xu** | 2023 | 1 | 1 | 2 | 2 | 1 | 2 | 2 | 2 | 2 | 1 | 1 | 2 |
| **Yamada** | 2016 | 1 | 1 | 2 | 2 | 1 | 2 | 2 | 2 | 2 | 1 | 2 | 2 |
| **Yamada** | 2015 | 1 | 1 | 2 | 2 | 1 | 2 | 2 | 2 | 2 | 1 | 2 | 2 |
| **Yaylaci** | 2021 | 1 | 1 | 2 | 2 | 2 | 2 | 2 | 2 | 2 | 2 | 1 | 2 |
| **Yeo** | 2020 | 2 | 2 | 2 | 2 | 2 | 2 | 2 | 2 | 2 | 1 | 2 | 2 |
| **Yoosoof** | 2022 | 2 | 2 | 2 | 2 | 2 | 2 | 2 | 2 | 2 | 1 | 2 | 2 |

^1^ Two person technique, Oropharyngeal airway (Mayo/Guedel), Laryngeal mask airway, Nasopharyngeal tube or Endotracheal tube

Abbreviations used in this tables:

AHA: American Heart Association

ECG: electrocardiogram

ERC: European Resuscitation Council

CC: chest compressions

CPR: cardiopulmonary resuscitation

GA: gestational age

HBB: Helping Babies Breathe

HR: heart rate

N/A: not applicable

NLS: Newborn/Neonatal life support

NRP: Neonatal Resuscitation Program

PEEP: postive end expiratory pressure

PPV: positive pressure ventilation

SpO2: peripheral oxygen saturation

**Table 3: Summary of the validity evidence of all included studies**

|  |  |  |  |  | **Classical frameworks** | | | | | | **Framework of Messick et al.** | | | | | **Framework of Kane et al.** | | | |
| --- | --- | --- | --- | --- | --- | --- | --- | --- | --- | --- | --- | --- | --- | --- | --- | --- | --- | --- | --- |
| **First author** | **Year of publication** | **Extra validity evidence gained by** | **Validity evidence available** | **Cronbach's alpha** | **Face** | **Content** | **Construct** | **Criterion** | **Interrater agreement** | **Intrarater agreement** | **Content** | **Internal structure** | **Relationships with other variables** | **Response process** | **Consequences** | **Scoring** | **Generalization** | **Extrapolation** | **Implications**  **/decisions** |
|  |  |  | 1 = Yes 2 = No / not retrievable | 1 = Yes 2 = No | 1 = Yes 2 = No | 1 = Yes 2 = No | 1 = Yes 2 = No | 1 = Yes 2 = No | 1 = Yes 2 = No | 1 = Yes 2 = No | 1 = Yes 2 = No | 1 = Yes 2 = No | 1 = Yes 2 = No | 1 = Yes 2 = No | 1 = Yes 2 = No | 1 = Yes 2 = No | 1 = Yes 2 = No | 1 = Yes 2 = No | 1 = Yes 2 = No |
| **HBB 1st edition** | 2010 | Arabi et al. 2016, Mistry et al. 2018, Seto et al. 2017 | 1 | 1 | 2 | 2 | 2 | 2 | 1 | 2 | 1 | 1 | 1 | 1 | 2 | 2 | 2 | 2 | 2 |
| **HBB 2nd edition** | 2016 | Hatamleh et al. 2021, Odongkara et al. 2020 | 1 | 2 | 2 | 1 | 2 | 2 | 1 | 2 | 2 | 2 | 2 | 2 | 2 | 2 | 2 | 2 | 2 |
| **ERC 2021** | 2021 |  | 2 | 2 | 2 | 2 | 2 | 2 | 2 | 2 | 2 | 2 | 2 | 2 | 2 | 2 | 2 | 2 | 2 |
| **Athanasopoulou,** | 2024 |  | 2 | 2 | 2 | 2 | 2 | 2 | 2 | 2 | 2 | 2 | 2 | 2 | 2 | 2 | 2 | 2 | 2 |
| **Barbato** | 2020 |  | 1 | 2 | 2 | 1 | 2 | 2 | 2 | 2 | 2 | 2 | 2 | 2 | 2 | 2 | 2 | 2 | 2 |
| **Barry** | 2012 |  | 1 | 2 | 2 | 1 | 1 | 2 | 1 | 2 | 2 | 2 | 2 | 2 | 2 | 2 | 2 | 2 | 2 |
| **Bender** | 2014 |  | 1 | 2 | 2 | 2 | 2 | 2 | 1 | 2 | 2 | 2 | 2 | 2 | 2 | 2 | 2 | 2 | 2 |
| **Bibl** | 2023 |  | 1 | 2 | 2 | 1 | 1 | 2 | 1 | 2 | 2 | 2 | 2 | 2 | 2 | 2 | 2 | 2 | 2 |
| **Billner-Garcia** | 2022 |  | 1 | 2 | 2 | 1 | 2 | 2 | 2 | 2 | 2 | 2 | 2 | 2 | 2 | 2 | 2 | 2 | 2 |
| **Binkhorst** | 2020 |  | 1 | 2 | 2 | 1 | 2 | 2 | 2 | 2 | 2 | 2 | 2 | 2 | 2 | 2 | 2 | 2 | 2 |
| **Brathwaite** | 2020 |  | 2 | 2 | 2 | 2 | 2 | 2 | 2 | 2 | 2 | 2 | 2 | 2 | 2 | 2 | 2 | 2 | 2 |
| **Brogaard** | 2022 |  | 1 | 2 | 2 | 1 | 2 | 2 | 1 | 2 | 2 | 2 | 2 | 2 | 2 | 2 | 2 | 2 | 2 |
| **Caldelari** | 2019 |  | 2 | 2 | 2 | 2 | 2 | 2 | 2 | 2 | 2 | 2 | 2 | 2 | 2 | 2 | 2 | 2 | 2 |
| **Campbell** | 2009 |  | 1 | 2 | 2 | 1 | 2 | 2 | 2 | 2 | 2 | 2 | 2 | 2 | 2 | 2 | 2 | 2 | 2 |
| **Carbine** | 2000 |  | 1 | 2 | 2 | 1 | 2 | 2 | 2 | 2 | 2 | 2 | 2 | 2 | 2 | 2 | 2 | 2 | 2 |
| **Carlo** | 2009 |  | 1 | 1 | 2 | 1 | 2 | 2 | 2 | 2 | 2 | 2 | 2 | 2 | 2 | 2 | 2 | 2 | 2 |
| **Cavallin** | 2021 |  | 2 | 2 | 2 | 2 | 2 | 2 | 2 | 2 | 2 | 2 | 2 | 2 | 2 | 2 | 2 | 2 | 2 |
| **Chan** | 2019 |  | 1 | 2 | 2 | 2 | 2 | 2 | 1 | 2 | 2 | 2 | 2 | 2 | 2 | 2 | 2 | 2 | 2 |
| **Clark** | 2022 |  | 2 | 2 | 2 | 2 | 2 | 2 | 2 | 2 | 2 | 2 | 2 | 2 | 2 | 2 | 2 | 2 | 2 |
| **Cordero** | 2013 |  | 1 | 2 | 2 | 2 | 2 | 2 | 1 | 2 | 2 | 2 | 2 | 2 | 2 | 2 | 2 | 2 | 2 |
| **Cordero** | 2014 |  | 1 | 2 | 2 | 2 | 2 | 2 | 1 | 2 | 2 | 2 | 2 | 2 | 2 | 2 | 2 | 2 | 2 |
| **Cronin** | 2001 |  | 1 | 2 | 2 | 1 | 2 | 2 | 2 | 2 | 2 | 2 | 2 | 2 | 2 | 2 | 2 | 2 | 2 |
| **Curran** | 2014 |  | 1 | 1 | 1 | 1 | 1 | 1 | 2 | 2 | 2 | 2 | 2 | 2 | 2 | 2 | 2 | 2 | 2 |
| **Cusack** | 2012 |  | 2 | 2 | 2 | 2 | 2 | 2 | 2 | 2 | 2 | 2 | 2 | 2 | 2 | 2 | 2 | 2 | 2 |
| **Das** | 2018 |  | 2 | 2 | 2 | 2 | 2 | 2 | 2 | 2 | 2 | 2 | 2 | 2 | 2 | 2 | 2 | 2 | 2 |
| **De Bernardo** | 2016 |  | 2 | 2 | 2 | 2 | 2 | 2 | 2 | 2 | 2 | 2 | 2 | 2 | 2 | 2 | 2 | 2 | 2 |
| **Delaney** | 2022 |  | 2 | 2 | 2 | 2 | 2 | 2 | 2 | 2 | 2 | 2 | 2 | 2 | 2 | 2 | 2 | 2 | 2 |
| **Ding** | 2021 |  | 2 | 2 | 2 | 2 | 2 | 2 | 2 | 2 | 2 | 2 | 2 | 2 | 2 | 2 | 2 | 2 | 2 |
| **Farhadi** | 2023 |  | 1 | 2 | 2 | 1 | 2 | 2 | 2 | 2 | 2 | 2 | 2 | 2 | 2 | 2 | 2 | 2 | 2 |
| **Ford** | 2023 |  | 1 | 2 | 2 | 1 | 2 | 2 | 2 | 2 | 2 | 2 | 2 | 2 | 2 | 2 | 2 | 2 | 2 |
| **Gamboa** | 2018 |  | 2 | 2 | 2 | 2 | 2 | 2 | 2 | 2 | 2 | 2 | 2 | 2 | 2 | 2 | 2 | 2 | 2 |
| **Gelbart** | 2010 |  | 1 | 2 | 2 | 1 | 2 | 2 | 2 | 2 | 2 | 2 | 2 | 2 | 2 | 2 | 2 | 2 | 2 |
| **Ghoman** | 2020 |  | 1 | 2 | 2 | 1 | 2 | 2 | 2 | 2 | 2 | 2 | 2 | 2 | 2 | 2 | 2 | 2 | 2 |
| **Greer** | 2023 |  | 2 | 2 | 2 | 2 | 2 | 2 | 2 | 2 | 2 | 2 | 2 | 2 | 2 | 2 | 2 | 2 | 2 |
| **Gross** | 2020 |  | 1 | 2 | 2 | 1 | 2 | 2 | 1 | 2 | 2 | 2 | 2 | 2 | 2 | 2 | 2 | 2 | 2 |
| **Holm-Hansen** | 2022 |  | 1 | 2 | 2 | 1 | 2 | 2 | 2 | 2 | 2 | 2 | 2 | 2 | 2 | 2 | 2 | 2 | 2 |
| **Horiuchi** | 2021 |  | 2 | 2 | 2 | 2 | 2 | 2 | 2 | 2 | 2 | 2 | 2 | 2 | 2 | 2 | 2 | 2 | 2 |
| **Hundscheid** | 2021 |  | 1 | 2 | 2 | 1 | 2 | 2 | 2 | 2 | 2 | 2 | 2 | 2 | 2 | 2 | 2 | 2 | 2 |
| **Johnson** | 2020 |  | 1 | 2 | 2 | 1 | 2 | 2 | 2 | 2 | 2 | 2 | 2 | 2 | 2 | 2 | 2 | 2 | 2 |
| **Kamath-Rayne** | 2019 |  | 2 | 2 | 2 | 1 | 2 | 2 | 2 | 2 | 2 | 2 | 2 | 2 | 2 | 2 | 2 | 2 | 2 |
| **Kamau** | 2022 |  | 1 | 2 | 2 | 1 | 2 | 2 | 2 | 2 | 2 | 2 | 2 | 2 | 2 | 2 | 2 | 2 | 2 |
| **Kannan Loganathan** | 2024 |  | 1 | 2 | 2 | 2 | 2 | 2 | 1 | 2 | 2 | 2 | 2 | 2 | 2 | 2 | 2 | 2 | 2 |
| **Kane** | 2019 |  | 1 | 2 | 2 | 1 | 1 | 2 | 1 | 2 | 1 | 1 | 2 | 1 | 2 | 2 | 1 | 2 | 2 |
| **Khriesat** | 2017 |  | 1 | 1 | 2 | 1 | 2 | 2 | 2 | 2 | 2 | 2 | 2 | 2 | 2 | 2 | 2 | 2 | 2 |
| **Kim** | 2013 |  | 2 | 2 | 2 | 2 | 2 | 2 | 2 | 2 | 2 | 2 | 2 | 2 | 2 | 2 | 2 | 2 | 2 |
| **Lizotte** | 2017 |  | 1 | 2 | 2 | 1 | 2 | 2 | 1 | 2 | 2 | 2 | 2 | 2 | 2 | 2 | 2 | 2 | 2 |
| **Lockyer** | 2006 | Bould et al. 2009, Katakam et al. 2012 | 1 | 1 | 1 | 1 | 1 | 1 | 1 | 2 | 2 | 2 | 2 | 2 | 2 | 2 | 2 | 2 | 2 |
| **Magee** | 2018 |  | 1 | 2 | 2 | 1 | 2 | 2 | 1 | 2 | 2 | 2 | 2 | 2 | 2 | 2 | 2 | 2 | 2 |
| **Maya-Enero** | 2018 |  | 1 | 2 | 2 | 1 | 2 | 2 | 2 | 2 | 2 | 2 | 2 | 2 | 2 | 2 | 2 | 2 | 2 |
| **McCaw** | 2023 |  | 1 | 2 | 2 | 1 | 2 | 2 | 2 | 2 | 2 | 2 | 2 | 2 | 2 | 2 | 2 | 2 | 2 |
| **Meggiolaro** | 2025 |  | 1 | 2 | 2 | 1 | 1 | 2 | 1 | 2 | 2 | 2 | 2 | 2 | 2 | 2 | 2 | 2 | 2 |
| **Mersha** | 2019 |  | 1 | 2 | 2 | 1 | 2 | 2 | 2 | 2 | 2 | 2 | 2 | 2 | 2 | 2 | 2 | 2 | 2 |
| **Mileder** | 2023 |  | 2 | 2 | 2 | 2 | 2 | 2 | 2 | 2 | 2 | 2 | 2 | 2 | 2 | 2 | 2 | 2 | 2 |
| **Nadler** | 2011 |  | 1 | 1 | 2 | 2 | 2 | 2 | 2 | 2 | 2 | 2 | 2 | 2 | 2 | 2 | 2 | 2 | 2 |
| **Nickerson** | 2019 |  | 1 | 2 | 2 | 1 | 2 | 2 | 1 | 2 | 2 | 2 | 2 | 2 | 2 | 2 | 2 | 2 | 2 |
| **Nvonako** | 2022 |  | 1 | 2 | 2 | 1 | 2 | 2 | 2 | 2 | 2 | 2 | 2 | 2 | 2 | 2 | 2 | 2 | 2 |
| **Paliatsiou** | 2021 |  | 1 | 1 | 2 | 1 | 2 | 2 | 2 | 2 | 2 | 2 | 2 | 2 | 2 | 2 | 2 | 2 | 2 |
| **Reisman** | 2015 |  | 1 | 2 | 2 | 2 | 2 | 2 | 1 | 2 | 2 | 2 | 2 | 2 | 2 | 2 | 2 | 2 | 2 |
| **Roitsch** | 2020 |  | 1 | 2 | 2 | 1 | 2 | 2 | 1 | 2 | 2 | 2 | 2 | 2 | 2 | 2 | 2 | 2 | 2 |
| **Rovamo** | 2013 |  | 1 | 1 | 2 | 2 | 2 | 2 | 2 | 2 | 2 | 2 | 2 | 2 | 2 | 2 | 2 | 2 | 2 |
| **Rovamo** | 2011 |  | 1 | 1 | 2 | 2 | 2 | 2 | 2 | 2 | 2 | 2 | 2 | 2 | 2 | 2 | 2 | 2 | 2 |
| **Sarvan** | 2022 |  | 1 | 2 | 2 | 1 | 2 | 2 | 2 | 2 | 2 | 2 | 2 | 2 | 2 | 2 | 2 | 2 | 2 |
| **Sawyer** | 2011 | Matterson et al. 2018 | 1 | 2 | 2 | 2 | 2 | 2 | 1 | 2 | 1 | 1 | 1 | 1 | 1 | 2 | 2 | 2 | 2 |
| **Shikuku** | 2017 |  | 1 | 2 | 2 | 1 | 2 | 2 | 2 | 2 | 2 | 2 | 2 | 2 | 2 | 2 | 2 | 2 | 2 |
| **Sinha** | 2024 |  | 2 | 2 | 2 | 2 | 2 | 2 | 2 | 2 | 2 | 2 | 2 | 2 | 2 | 2 | 2 | 2 | 2 |
| **Sintayehu** | 2020 |  | 2 | 2 | 2 | 2 | 2 | 2 | 2 | 2 | 2 | 2 | 2 | 2 | 2 | 2 | 2 | 2 | 2 |
| **Skâre** | 2018 |  | 1 | 2 | 2 | 2 | 2 | 2 | 1 | 1 | 2 | 2 | 2 | 2 | 2 | 2 | 2 | 2 | 2 |
| **Sloane** | 2021 |  | 1 | 1 | 2 | 2 | 2 | 2 | 1 | 2 | 2 | 2 | 2 | 2 | 2 | 2 | 2 | 2 | 2 |
| **Szyld** | 2021 |  | 1 | 2 | 2 | 1 | 2 | 2 | 1 | 2 | 2 | 2 | 2 | 2 | 2 | 2 | 2 | 2 | 2 |
| **Tosif** | 2020 |  | 2 | 2 | 2 | 2 | 2 | 2 | 2 | 2 | 2 | 2 | 2 | 2 | 2 | 2 | 2 | 2 | 2 |
| **Trevisanuto** | 2015 |  | 1 | 2 | 2 | 1 | 2 | 2 | 2 | 2 | 2 | 2 | 2 | 2 | 2 | 2 | 2 | 2 | 2 |
| **Tsang** | 2022 |  | 1 | 2 | 2 | 1 | 2 | 2 | 1 | 2 | 2 | 2 | 2 | 2 | 2 | 2 | 2 | 2 | 2 |
| **Vail** | 2018 |  | 2 | 2 | 2 | 2 | 2 | 2 | 2 | 2 | 2 | 2 | 2 | 2 | 2 | 2 | 2 | 2 | 2 |
| **Vail** | 2017 |  | 1 | 2 | 2 | 2 | 2 | 2 | 1 | 2 | 2 | 2 | 2 | 2 | 2 | 2 | 2 | 2 | 2 |
| **Van der Heide** | 2006 | Lee et al. 2012 | 1 | 2 | 1 | 1 | 1 | 2 | 1 | 1 | 2 | 2 | 2 | 2 | 2 | 2 | 2 | 2 | 2 |
| **Woods** | 2015 |  | 1 | 1 | 2 | 2 | 2 | 2 | 2 | 2 | 2 | 2 | 2 | 2 | 2 | 2 | 2 | 2 | 2 |
| **Xu** | 2023 |  | 1 | 2 | 2 | 1 | 2 | 2 | 2 | 2 | 2 | 2 | 2 | 2 | 2 | 2 | 2 | 2 | 2 |
| **Yamada** | 2016 |  | 1 | 2 | 2 | 1 | 2 | 2 | 2 | 2 | 2 | 2 | 2 | 2 | 2 | 2 | 2 | 2 | 2 |
| **Yamada** | 2015 |  | 1 | 2 | 2 | 1 | 2 | 2 | 2 | 2 | 2 | 2 | 2 | 2 | 2 | 2 | 2 | 2 | 2 |
| **Yaylaci** | 2021 |  | 1 | 2 | 2 | 1 | 2 | 2 | 1 | 2 | 2 | 2 | 2 | 2 | 2 | 2 | 2 | 2 | 2 |
| **Yeo** | 2020 |  | 2 | 2 | 2 | 2 | 2 | 2 | 2 | 2 | 2 | 2 | 2 | 2 | 2 | 2 | 2 | 2 | 2 |
| **Yoosoof** | 2022 |  | 1 | 2 | 2 | 1 | 2 | 2 | 2 | 2 | 2 | 2 | 2 | 2 | 2 | 2 | 2 | 2 | 2 |

Abbreviations used in this table:

ERC: European Resuscitation Council

HBB: Helping Babies Breathe
